# Arabidopsis RNA processing body components LSM1 and DCP5 aid in the evasion of translational repression during *Cauliflower mosaic virus* infection

**DOI:** 10.1093/plcell/koac132

**Published:** 2022-05-02

**Authors:** Gesa Hoffmann, Amir Mahboubi, Heinrich Bente, Damien Garcia, Johannes Hanson, Anders Hafrén

**Affiliations:** Department of Plant Biology, Uppsala BioCenter, Swedish University of Agricultural Sciences, Uppsala 75007, Sweden; Linnean Center for Plant Biology, Uppsala 75007, Sweden; Department of Plant Physiology, Umeå Plant Science Centre, Umeå University, Umeå, Sweden; Department of Plant Biology, Uppsala BioCenter, Swedish University of Agricultural Sciences, Uppsala 75007, Sweden; Linnean Center for Plant Biology, Uppsala 75007, Sweden; Institut de Biologie Moléculaire des Plantes, CNRS, Université de Strasbourg, Strasbourg, France; Department of Plant Physiology, Umeå Plant Science Centre, Umeå University, Umeå, Sweden; Department of Plant Biology, Uppsala BioCenter, Swedish University of Agricultural Sciences, Uppsala 75007, Sweden; Linnean Center for Plant Biology, Uppsala 75007, Sweden

## Abstract

Viral infections impose extraordinary RNA stress, triggering cellular RNA surveillance pathways such as RNA decapping, nonsense-mediated decay, and RNA silencing. Viruses need to maneuver among these pathways to establish infection and succeed in producing high amounts of viral proteins. Processing bodies (PBs) are integral to RNA triage in eukaryotic cells, with several distinct RNA quality control pathways converging for selective RNA regulation. In this study, we investigated the role of *Arabidopsis thaliana* PBs during *Cauliflower mosaic virus* (CaMV) infection. We found that several PB components are co-opted into viral factories that support virus multiplication. This pro-viral role was not associated with RNA decay pathways but instead, we established that PB components are helpers in viral RNA translation. While CaMV is normally resilient to RNA silencing, dysfunctions in PB components expose the virus to this pathway, which is similar to previous observations for transgenes. Transgenes, however, undergo RNA quality control-dependent RNA degradation and transcriptional silencing, whereas CaMV RNA remains stable but becomes translationally repressed through decreased ribosome association, revealing a unique dependence among PBs, RNA silencing, and translational repression. Together, our study shows that PB components are co-opted by the virus to maintain efficient translation, a mechanism not associated with canonical PB functions.

## Teaser

Arabidopsis RNA processing body components LSM1 and DCP5 are co-opted by *Cauliflower mosaic virus* to maintain efficient virus translation in the presence of RNA DEPENDENT POLYMERASE6-governed silencing.

IN A NUTSHELL **Background:** Viruses are unique in their ability to reuse and recycle host proteins and other components for their own benefit. *Cauliflower mosaic virus* (CaMV) forms special structures inside the host cells known as viral factories (VFs) to facilitate efficient replication and escape defense. VFs consist of viral proteins, as well as particles and nucleic acids, but also numerous host proteins and ribosomes that are co-opted into these structures. Building on knowledge from the animal field, RNA granules, including stress granules and processing bodies (PBs), are at the forefront of viral disease regulation. Several granule-localized proteins directly interact and influence virus replication.

**Question:** We investigated the role of PB components in CaMV infection. We wanted to elucidate the interplay from two sides: What is the effect of CaMV infection on the localization and abundance of PB components, but also how do these proteins influence CaMV replication and especially viral protein production?

**Findings:** Decapping proteins DCP5 and LSM1 localize to VFs during CaMV infection. CaMV DNA and protein accumulation, but not RNA levels, are reduced in Arabidopsis *dcp5* and *lsm1* mutants. We found that viral RNA is not a target of LSM1-mediated decapping and that RNA stability is not affected in either mutant. We examined *dcp5* and *lsm1* single mutants as well as double mutants with *RNA-dependent RNA polymerase 6* (*rdr6*), finding that less viral RNA was associated with ribosomes in the single but not double mutants. Thus, PB proteins help the virus evade translational repression by RDR6.

**Next steps:** We do not yet know how RDR6 mediates translational repression of viral RNA in the absence of DCP5 or LSM1. Elucidating the exact mechanism and which roles the VF and viral proteins play in this interaction will help further our understanding of plant virus infections.

## Introduction

Eukaryotic gene expression is tightly regulated from RNA transcription to translation and decay. The importance of posttranscriptional control, especially during stress-induced cellular reprogramming, is becoming increasingly evident, as several studies have revealed extensive uncoupling between transcriptomes and translatomes (Branco-Price et al., 2005; Tebaldi et al., 2012; Liu et al., 2013; Zid and O'Shea, 2014; Xu et al., 2017).

Due to the high energy cost and possible detrimental effects of uncontrolled protein translation, eukaryotic cells have evolved a network of pathways to govern and regulate mRNA translation, including the “mRNA cycle” (Buchan and Parker, 2009). Here, cytoplasmic mRNAs are channeled between ribosomes and phase-separated cytoplasmic ribonucleoprotein (RNP) complexes, the RNA granules, in a triage between translation, nontranslating storage, and degradation. Several types of RNA granules have been identified and defined by their core protein constituents (Chantarachot and Bailey-Serres, 2018; Xing et al., 2020). The mRNA cycle involves two major types of RNA granules, processing bodies (PBs) and stress granules (SGs). RNAs are thought to shuffle between active translation at ribosomes and translationally repressed states at SGs (Buchan and Parker, 2009). In contrast, the localization of RNAs to PBs is mainly associated with RNA degradation owing to the absence of translation initiation factors and the highly conserved PB core components involved in RNA nonsense-mediated decay (NMD), miRNA-targeted gene silencing, deadenylation, and decapping (Anderson and Kedersha, 2009). Yet, while PB proteins can facilitate translational repression (Xu and Chua, 2009), recent studies have shown that PB-associated mRNAs can be stabilized and return to translation, expanding the multifunctionality of these RNA granules (Hubstenberger et al., 2017; Wang et al., 2018; Jang et al., 2019).

One hallmark of PBs is the accumulation of proteins required for mRNA decapping. This process involves the removal of the 7-methyl-guanosine 5′-diphosphate (cap) and is essential for subsequent 5′- to 3′-end mRNA degradation. In *Arabidopsis thaliana* (Arabidopsis), decapping is carried out by the nudix hydrolase DECAPPING2 (DCP2) and its co-factors DCP1 and VARICOSE (VCS; Xu et al., 2006). Several proteins function in decapping activation and PB assembly, including DCP5 and the SM-like (LSM) 1–7 complex (Xu and Chua, 2009; Perea-Resa et al., 2012). Uncapped RNAs are degraded by the cytoplasmic EXORIBONUCLEASE 4 (XRN4), which was also shown to accumulate in PBs (Souret et al., 2004; Yu et al., 2019). The decapping machinery is one part of the extensive RNA surveillance network present in PBs and is tightly connected to NMD (Chicois et al., 2018). NMD is governed by the surveillance protein UP FRAMESHIFT1 (UPF1), which in combination with other factors monitors RNAs for insufficient translation termination or the presence of exon junction complexes in the 3′-untranslated region (UTR) and subsequently induces their degradation. Interestingly, UPF1 not only associates with PBs but was also found to co-localize and shuffle between another class of cytoplasmic RNP granules, the small interfering (si)RNA bodies (Moreno et al., 2013). siRNA bodies are condensates of RNA-DEPENDENT POLYMERASE6 (RDR6), SUPRESSOR OF GENE SILENCING3 (SGS3), and ARGONAUTE7, as well as other posttranscriptional gene silencing (PTGS) factors (Jouannet et al., 2012). These bodies can localize adjacent to PBs and are proposed to store translationally repressed RNAs to triage them between PBs and RDR6-dependent PTGS, potentially through their interactions with UPF1 (Jouannet et al., 2012; Moreno et al., 2013).

Apart from their physical association, several connections and a tight inter-dependence of the RNA quality control (RQC) machinery and PTGS have been discovered in plants (Liu and Chen, 2016). An initial observation was the susceptibility of transgenes to suppression by RNA silencing in Arabidopsis *dcp2* mutants (Thran et al., 2012). Subsequently, decapping mutants were found to accumulate novel classes of endogenous siRNAs that arose through the cytoplasmic RDR6 pathway (Martinez de Alba et al., 2015). In line with the central role of RDR6 in this process, its knockout rescued the seedling lethality in the severe decapping mutants *vcs6* and *dcp2* (Martinez de Alba et al., 2015). The fact that major cytoplasmic RQC pathways and PTGS converge in PBs (Chantarachot and Bailey-Serres, 2018) makes these RNA granules prime targets for virus resistance and manipulation by viruses.

Viruses challenge the RQC and PTGS machineries through their massive production of RNAs during replication, and the targeting of viral RNAs by RNA silencing is one of the major defense pathway plants employ against viruses. In turn, viruses have frequently evolved RNA silencing suppressors to overcome this silencing (Csorba et al., 2015). The roles of PBs during plant viral infections are currently not well understood, but initial findings suggest that some viruses may benefit from PBs or their components via reduced targeting by antiviral RNA silencing (Hafrén et al., 2015; Ye et al., 2015).

In this study, we investigated the roles of PBs and decapping components in viral infection using the pararetrovirus *Cauliflower mosaic virus* (CaMV; family *Caulimoviridae*) and the model plant Arabidopsis. CaMV is a double-stranded DNA virus that harbors seven open-reading frames in two mRNAs transcribed from two promotors (*19s* and *35s*). While *35s* RNA encodes all viral proteins, *19s* RNA only encodes the viral transactivator protein P6. P6 is a highly abundant, essential protein that assembles in large cytoplasmic aggregates termed viral factories (VFs) that are the site of viral translation, reverse transcription, and particle packaging (Schoelz and Leisner, 2017). We show that at least three hallmark proteins of PBs are targeted to the VFs of CaMV and that these proteins are important for virus accumulation. We demonstrate that PBs serve a pro-viral role during CaMV infection by alleviating translational repression through RNA silencing.

## Results

### PB components re-localize during CaMV infection

**Figure 1:**
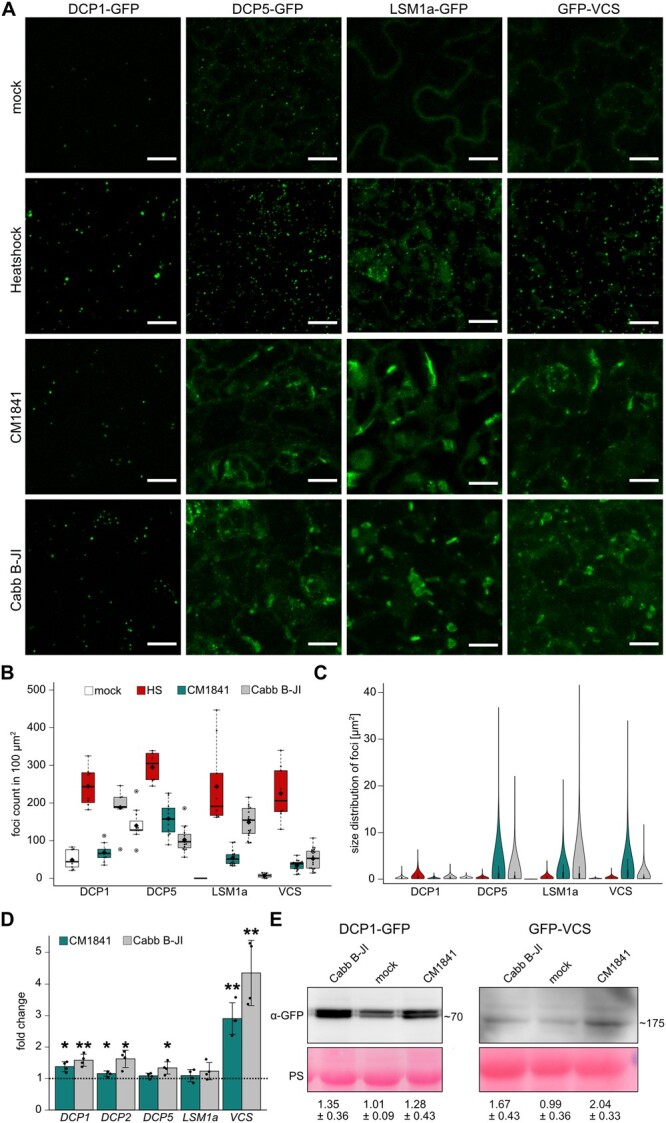
CaMV infection induces PB protein re-localization. A, Localization of four canonical PB markers under control conditions, after HS, and 21 dpi with CaMV strains CM1841 and Cabb B-JI. The representative images are composed of confocal Z-stacks (Scale bars = 10 µm). B, Count of fluorescent foci in 100 µm^2^ corresponding to the treatments in (A). Counts were performed from randomly chosen areas using ImageJ and a custom pipeline. The box represents the interquartile range (IQR), the solid lines represent the median, diamonds the average. The whiskers extend to a maximum of 1.5× IQR beyond the box. C, Size distribution of detected foci corresponding to (B). B and D, Values calculated from nine z-stacks of three plants per replicate. All experiments were replicated at least 3 times independently. D, Relative expression (fold change) of PB components 21 dpi compared to mock (dashed line). Values represent means ± standard deviation (sd; *n* = 4) relative to Col-0 plants and normalized to *PP2a* as the internal reference. The experiment was repeated 3 times independently. Error bars represent sd. Statistical significance was determined by Student *t* test (*P ≤ 0.05; **P ≤ 0.01). E, Immunoblot analysis of DCP1-GFP and GFP-VCS in systemic leaves of infected marker lines. Total proteins were extracted at 21 dpi and probed with GFP-antibodies. Ponceau S (PS) staining served as a loading control. Numbers indicate average (±sd) of protein abundance from three independent blots from independent infections quantified with ImageJ. Numbers on the side of the blot indicate the molecular weights of fusion proteins (kDa).

To visualize PB dynamics during CaMV infection, we used marker lines expressing GFP-tagged canonical PB proteins (DCP1pro:DCP1-GFP, UBQ10pro:DCP5-GFP, UBQ10pro:LSM1a-GFP, and VCSpro:GFP-VCS) (Motomura et al., 2012; Roux et al., 2015; Chicois et al., 2018). Under mock conditions, the markers showed a cytoplasmic distribution with varying degrees of condensation into droplet-like foci (Figure 1A). LSM1a–GFP fusion protein accumulated evenly in the cytoplasm, with no visible PB assembly, while GFP-VCS and DCP5-GFP were both present in foci and soluble, and DCP1-GFP mainly assembled in foci. These localization patterns were similar to those described previously (Motomura et al., 2015; Roux et al., 2015; Perea-Resa et al., 2016; Chicois et al., 2018). Before analyzing infection dynamics, we established how the markers behaved after heat shock (HS) application (Motomura et al., 2015). The number of detectable foci after HS increased drastically and was comparable for all markers, pointing toward directed co-assembly during stress (Figure 1, A and B). This is consistent with earlier findings that some PB proteins, including LSM1a, associate with PBs only upon stress (Perea-Resa et al., 2016; Guzikowski et al., 2019). Importantly, this analysis confirmed the functionality of the marker lines under our conditions.

Upon infection with two CaMV strains (CM1841 and Cabb B-JI), the PB marker proteins formed two morphologically distinct classes of visible structures in systemic leaves 21-day postinfection (dpi; Figure 1A), while free GFP localization remained unchanged (Supplemental Figure S1B). The number of DCP1-marked foci especially increased during Cabb B-JI infection, without any apparent change in morphology (Figure 1, A–C; Supplemental Figure S1A). The markers LSM1a, VCS, and DCP5 also accumulated in small DCP1-like foci upon CaMV infection, but most striking was their prominent assembly into large, irregularly shaped structures not seen with DCP1 (Figure 1, A–C; Supplemental Figure S1A). The large structures were less abundant than the droplets for the three markers and had a distorted circularity, which was not seen after HS or in the DCP1 marker (Figure 1B; Supplemental Figure S1A). We never detected comparable structures under either control conditions or after HS with any of the markers, while they were found abundantly with both CaMV strains, with slight variations in number and size. These structures grew in size and decreased in number during the infection time course, indicating their fusion in infected cells (Supplemental Figure S1B). To validate the findings and confirm that the same structures were indeed marked by different PB markers, we established two double marker lines with GFP-VCS/DCP1-RFP and GFP-VCS/LSM1a-RFP. DCP1 and LSM1a showed the same localization pattern regardless of which fluorescent marker was used. Interestingly, only a fraction of DCP1 and VCS co-localized under mock conditions, while co-assembly after HS again confirmed the stress-dependent co-accumulation of PB markers (Supplemental Figure S2). During CaMV infection, LSM1a-RFP and GFP-VCS both marked the same large, irregular structures, while DCP1-RFP localized to smaller foci adjacent to VCS structures (Supplemental Figure S2).

The localization of PB components to virus-induced structures led us to test whether the transcription of these components was altered during infection. The transcript levels were consistently elevated for *DCP1*, *DCP2*, and more strongly for *VCS* with both CaMV strains, while *DCP5* expression was only induced during Cabb B-JI infection, and *LSM1a* expression was not responsive to either strain (Figure 1D). Accordingly, immunoblot analysis confirmed that DCP1-GFP and GFP-VCS protein levels increased during infection (Figure 1E). In conclusion, CaMV infection causes condensation and a drastic re-localization of several PB proteins into large virus-induced structures.

### CaMV sequesters PB components into VFs

**Figure 2:**
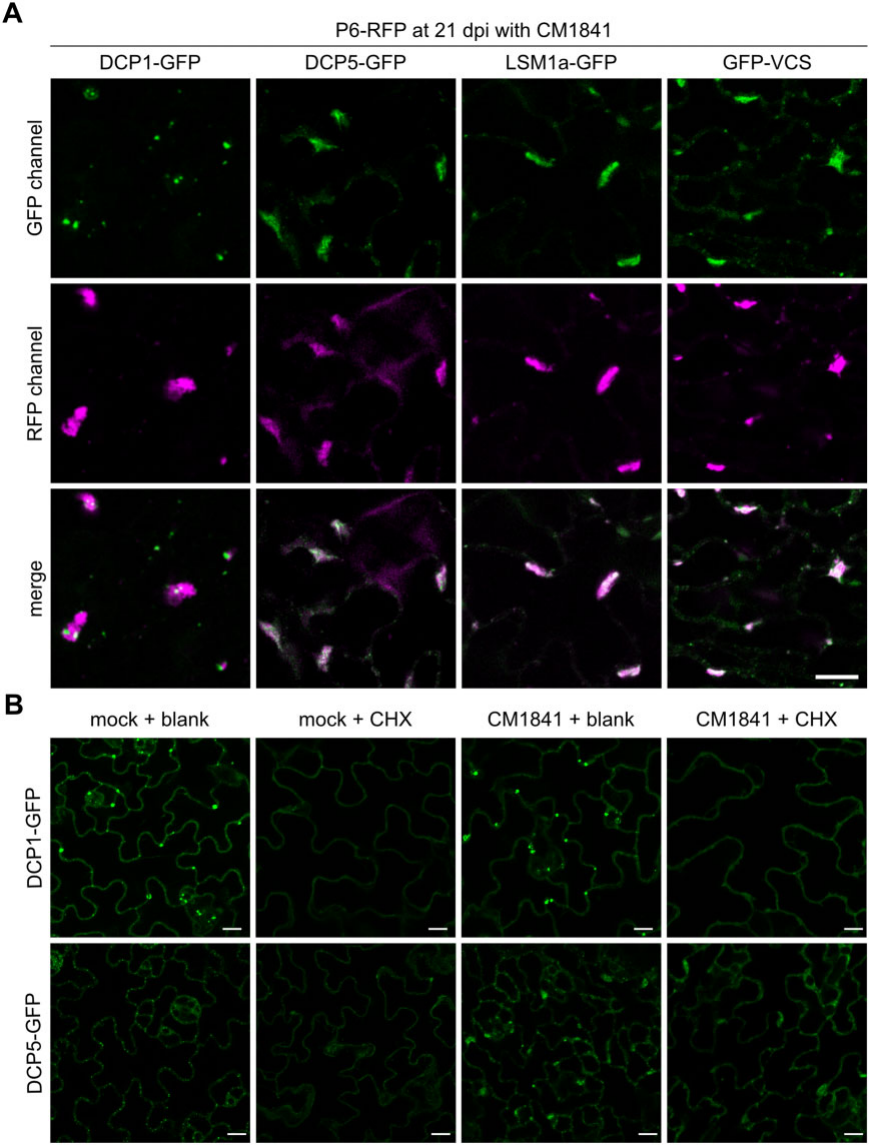
Virus-induced PB protein localization in viral factories. A, Co-localization of P6-RFP with GFP-tagged PB markers in transgenic Arabidopsis 21 dpi with CaMV strain CM1841. Representative single plane images are shown (Scale bars = 10 µm). The experiments were replicated in independent transformants. B, Distribution of DCP1-GFP and DCP5-GFP marker 21 days after mock or CaMV infection and 1 h after 200 µM CHX or blank infiltration. Images represent single plane micrographs (Scale bars = 10 µm). DCP1-GFP was imaged with a higher exposure to ensure visualization of the soluble fraction.

The re-localization of LSM1, VCS, and DCP5 into novel structures during CaMV infection, suggested that these structures could be virus-induced inclusions. CaMV assembles two types of cytoplasmic inclusions: the spherical transmission bodies that are mainly formed by the viral protein P2, and the more irregularly shaped VFs that are mainly formed by the viral protein P6 (Martelli and Castellano, 1971; Espinoza et al., 1991). Heterologous co-expression of six CaMV proteins with PB proteins in *Nicotiana benthamiana* showed that viral P6 protein co-localized with DCP1, DCP5, and VCS (Supplemental Figure S3). This prompted us to investigate co-localization of PB markers with VFs during CaMV infection. We used transgenic P6-mRFP expressing PB marker lines to investigate the association of DCP1, DCP5, LSM1, and VCS with VFs. Under control conditions, P6-mRFP was mostly soluble in the cytoplasm, with occasional foci formation (Supplemental Figure S4). Some, but not all these foci co-localized with DCP1, DCP5, and VCS, indicating that these proteins already associated in the absence of infection (Supplemental Figure S4, white arrows). During infection, the P6-mRFP protein assembled to mark the characteristic large VFs, which also accumulated DCP5, LSM1a, and VCS (Figure 2A). DCP1 foci accumulated around, but not within the VFs.

Translation inhibition through the trapping of ribosomes on mRNA by Cycloheximide (CHX) leads to the disassembly of canonical PBs (Teixeira et al., 2005; Motomura et al., 2015). Under our conditions, CHX treatment of the DCP1-GFP and DCP5-GFP marker line after mock or CaMV infection confirmed the dissociation of canonical PBs after CHX treatment. However, the irregular VFs were still marked by DCP5 in CHX-treated samples, albeit at lower signal intensity (Figure 2B). DCP1 bodies disappeared after treatment regardless of viral infection (Figure 2B). These results indicate that DCP5 in VFs is dynamically less responsive to depletion of the RNA supply from ribosomes than canonical PBs, possibly owing to VF size or other distinct physicochemical properties, including interactions with the VF matrix.

### Disruption of PB functions attenuates CaMV infection

**Figure 3:**
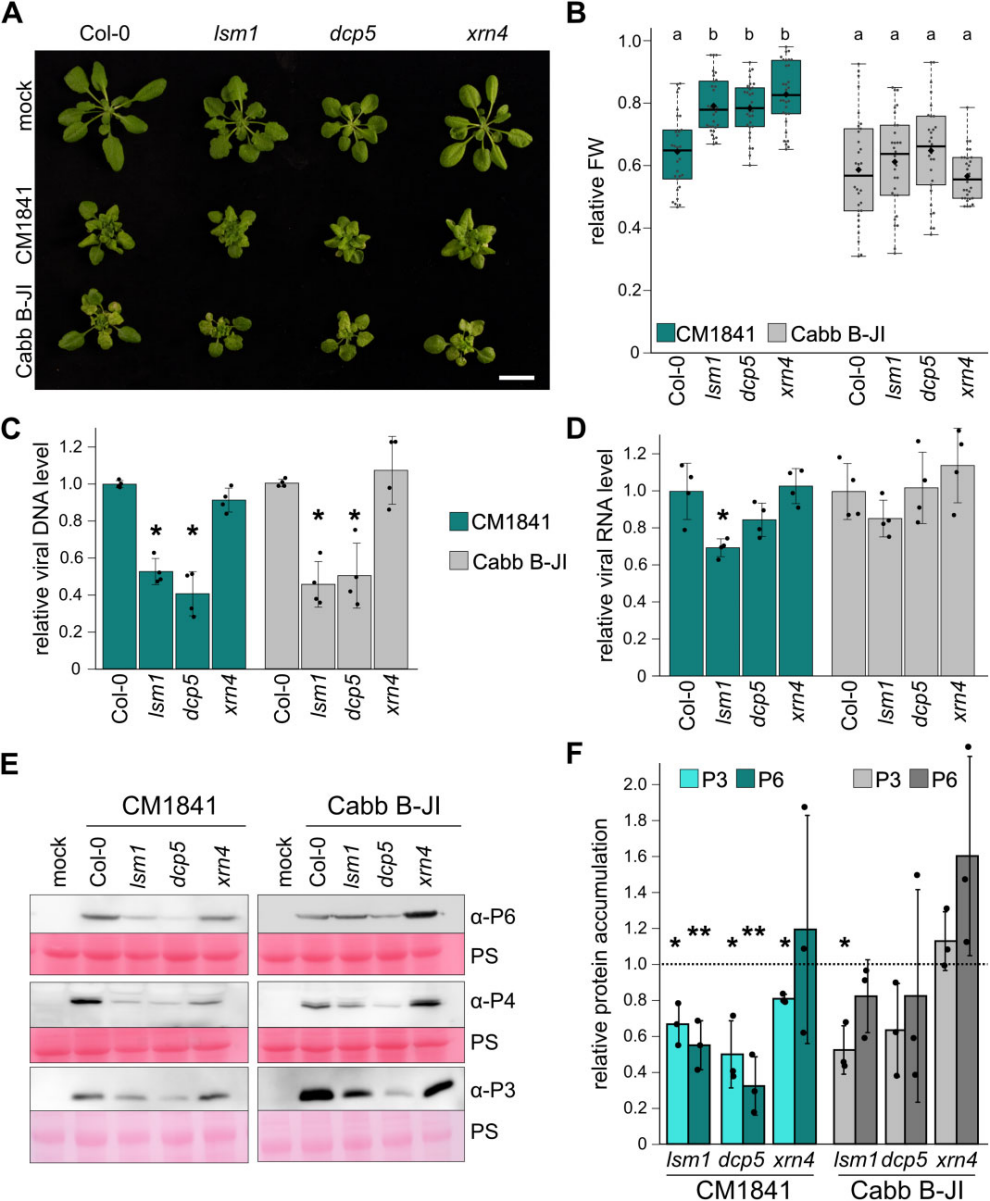
CaMV disease is attenuated in *lsm1* and *dcp5* mutants. A, Virus-induced symptoms in Col-0, *lsm1*, *dcp5*, and *xrn4* plants at 21 dpi. CM1841 and Cabb B-JI-infected plants are compared to mock-infected plants (Scale bar = 2 cm). B, Relative fresh weight of CaMV-infected compared to mock plants at 21 dpi (*n* = 30). The box represents the IQR, the solid lines represent the median, diamonds the average. The whiskers extend to a maximum of 1.5× IQR beyond the box. Statistical significance was determined by one-way ANOVA coupled with Tukey’s HSD test (*α* = 0.05), letters indicate statistical groups. C, Viral DNA accumulation in systemic leaves of Col-0 and mutant plants at 21 dpi, determined by qRT-PCR. Values represent means ± sd (*n* = 4) relative to Col-0 plants and normalized to *18S* ribosomal DNA as the internal reference. D, *35s* RNA levels of CaMV were determined by qRT-PCR in systemic leaves at 21 dpi. Values represent means ± sd (*n* = 4) relative to Col-0 plants and normalized to *PP2a*. E, Immunoblot analysis of CaMV P3, P4, and P6 proteins in the systemic leaves of Col-0, *lsm1*, *dcp5*, and *xrn4* plants Total proteins were extracted at 21 dpi and probed with specific antibodies. Mock-infected plants were used as a control for signal background. Ponceau S (PS) staining served as a loading control. F, Accumulation of CaMV P3 and P6 proteins in all genotypes in systemic leaves at 21 dpi quantified by direct ELISA. Values represent means ± SD (*n* = 3) in arbitrary units relative to Col-0 plants (dashed line). Statistical significance was determined by Student’s *t* test for (C, D, and F) (*P ≤ 0.05; **P ≤ 0.01). All experiments (A–F) were repeated at least 3 times from independent infections.

The VFs formed by CaMV P6 protein are electron dense, RNA-, and protein-rich structures with essential roles in the viral lifecycle (Martelli and Castellano, 1971; Schoelz and Leisner, 2017). VFs are proposed to be sites of active viral RNA translation, reverse transcription, and packaging of viral genomic DNA in particles. Considering the re-localization of PB components to viral replication sites, we next investigated the role of PB components in CaMV disease by analyzing infection phenotypes in mutants affected in PB formation. The null mutant *lsm1a/b* (hereafter referred to as *lsm1*) and knockdown mutant *dcp5* were chosen for this study, because both mutations cause a reduction in PB formation and PB size, as well as an over-accumulation of capped mRNAs (Xu and Chua, 2009; Perea-Resa et al., 2012, 2016). Importantly, these mutants are not postembryonic lethal, in contrast to null mutants of *DCP1*, *DCP2*, and *VCS* (Xu et al., 2006), and grow well enough for virus infection experiments. The *lsm1* and *dcp5* plants showed developmental phenotypes, including slightly delayed germination, mild dwarfism, and leaf serrations (Figure 3A). Additionally, the null-mutant of the cytoplasmic exonuclease *xrn4* was used; this mutant is not impaired in PB biogenesis and mRNA decapping, but it over-accumulates uncapped RNAs (Nagarajan et al., 2019). The *xrn4* plants were morphologically not distinguishable from Col-0 plants under short-day conditions but showed the typical serrations under long-day conditions.

Upon infection with CaMV, all mutants showed similar levels of stunting, vein bleaching, rosette distortion, and leaf wrinkling compared to Col-0 (Figure 3A), with prominent symptoms appearing at 12 (Cabb B-JI) or 14 (CM1841) dpi. The relative fresh weight of CaMV-infected compared to mock-inoculated plants was taken as a measure of disease severity. The fresh weight loss was less severe in all three mutants compared to Col-0 for the milder CM1841 strain and unaltered for Cabb B-JI (Figure 3B). In general, Cabb B-JI infection caused stronger but also more variable infection phenotypes, possibly masking potential effects of PB disruption on fresh weight loss.

To establish viral load in the mutants compared to Col-0, we measured viral DNA, RNA, and protein levels. Viral DNA accumulation was attenuated for both CaMV strains in *lsm1* and *dcp5*, but not in the *xrn4* mutant (Figure 3C). In addition, we analyzed CM1841 DNA levels in heterozygous plants of the embryo lethal *dcp1-1*, *dcp2-1*, and *vcs6* mutants as well as the homozygous knockdown mutant *dcp1-3* (Martinez de Alba et al., 2015), but we did not detect a defect in viral titer in any of these lines (Supplemental Figure S5). This suggests that *dcp1-3* and the heterozygous lines are weaker mutants compared to *dcp5* and *lsm1*, as supported by the absence of morphological defects. Alternatively, there may be a specific involvement of LSM1 and DCP5, independent of decapping, but the localization of VCS along with these components to VFs would argue against this. Viral DNA is produced through reverse transcription of the viral *35s* RNA. Interestingly, the levels of *35s* RNA were only mildly reduced for CM1841 and remained unaffected for Cabb B-JI in *lsm1* and *dcp5* (Figure 3D), suggesting that reduced DNA levels could be caused by defects in viral RNA usage in translation or reverse transcription rather than RNA production.

Immunoblot analysis showed that less of the viral inclusion protein P6, the coat protein P4, and the virion-associated protein P3 accumulated in both *lsm1* and *dcp5* compared to Col-0 (Figure 3E). Viral protein accumulation in *xrn4* differed between the two strains, with CM1841 showing a mild reduction in P6 and P4 levels, while Cabb B-JI showed higher levels of P6 and P4. A direct enzyme-linked immunosorbent assay (ELISA) confirmed reduced P6 and P3 accumulation in *lsm1* and *dcp5* for CM1841 and also Cabb B-JI, albeit the effect was weaker (Figure 3F). In combination, the impairment of CaMV disease in these mutants indicates that PB components play a pro-viral role during CaMV infection. Virus accumulation was impaired in mutants defective in PB biogenesis and decapping (*lsm1* and *dcp5*), but not in exonucleolytic RNA decay (*xrn4*). Owing to the similarities between the two strains, we continued our subsequent analysis with the milder CM1841 strain.

### LSM1 has no major role in viral RNA stability or decapping

**Figure 4:**
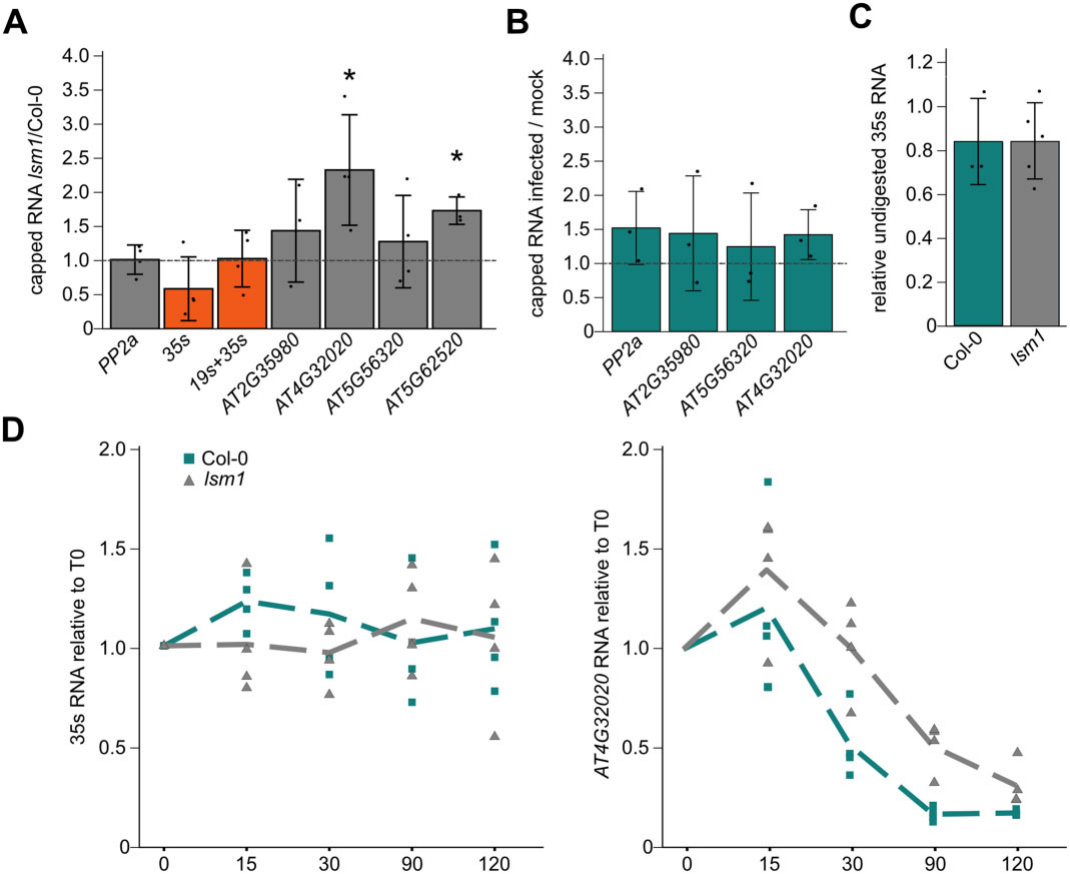
LSM1 does not regulate viral RNA stability. A, RNA levels detected after cap-dependent pulldown in infected *lsm1* compared to Col-0 plants for the housekeeping gene *PP2a*, viral RNA, and four previously described LSM1 targets. Bars represent mean from independent pulldowns from independent infections (*n* = 4). B, RNA levels detected after cap-dependent pulldown on endogenous RNAs from CaMV infected tissue compared to mock infected. Bars represent mean from independent pulldowns from independent infections (*n* = 3). C, Amount of viral *35s* RNA in Col-0 and *lsm1* mutant detected after 1h of XRN1 treatment. Bars represent the mean from independent digestions from independent infections (*n* = 3 for Col-0; *n* = 5 for *lsm1*). Statistical significance was determined by Student’s *t* test for (A–C) (*P ≤ 0.05). D, Transcript decay profiles for viral *35s* and *AT4G32020* RNA after transcriptional arrest using cordycepin. Dotted line represents the average of four independent experiments, single experiments are shown by circles (Col-0) and triangles (*lsm1*). Sampling timepoints are indicated on the *x*-axis (0- to 120-min past treatment).

The established role of LSM1 and DCP5 in RNA decapping and degradation led us to test whether these PB-associated factors were acting on viral RNA during infection, as the seemingly unaltered viral RNA levels in *lsm1* and *dcp5* mutants (Figure 3D) could still be explained by a combination of reduced transcription and a defect in RNA decay. To determine the capping levels of viral RNAs in Col-0 and *lsm1* plants, we performed an RNA-pulldown experiment with cap-specific antibodies (Golisz et al., 2013). We found known targets of LSM1-mediated decapping to be more abundant in their capped form in the *lsm1* mutant, as expected from previous studies (Perea-Resa et al., 2012; Golisz et al., 2013), while the capping levels of CaMV *35s* and *19s* RNA did not differ between Col-0 and *lsm1* (Figure 4A). Furthermore, a comparison of known LSM1 targets between the control and CaMV-infected samples showed that viral infection does not influence decapping of those endogenous targets, although we cannot exclude the possibility that other targets might be affected (Figure 4B).

Unaltered capping of viral RNA was further supported by a cap-sensitive exonuclease digestion of total RNA from infected plants, showing identical susceptibility of viral *35s* RNA isolated from the *lsm1* mutant compared to Col-0 (Figure 4C). Considering the possibility of decapping-independent RNA decay, we also tested whether the decay rate of viral *35s* RNA was altered in *lsm1* mutants by quantifying RNA from infected rosettes in a time course after inducing transcriptional arrest using Cordycepin (Sorenson et al., 2018). CaMV RNA was remarkably stable and showed no sign of degradation after 120 min of transcriptional inhibition in Col-0, *lsm1* (Figure 4D), *dcp5*, and *xrn4* (Supplemental Figure S6A). A longer treatment time of 8 h still showed no evident degradation of viral RNA (Supplemental Figure S6B), indicating that the viral RNA is strongly protected. The degradation profile of *AT4G32020*, a known target of LSM1-dependent decapping (Golisz et al., 2013), confirmed the transcriptional inhibition and LSM1-dependent effects (Figure 4D). Our results support that CaMV RNAs are not major targets of LSM1-dependent decapping or decay and thus, these dysfunctions in *lsm1* and *dcp5* are not likely to cause the reduced virus accumulation observed in these mutants.

### Defects in LSM1/DCP5 expose CAMV to RNA silencing but not NMD

**Figure 5:**
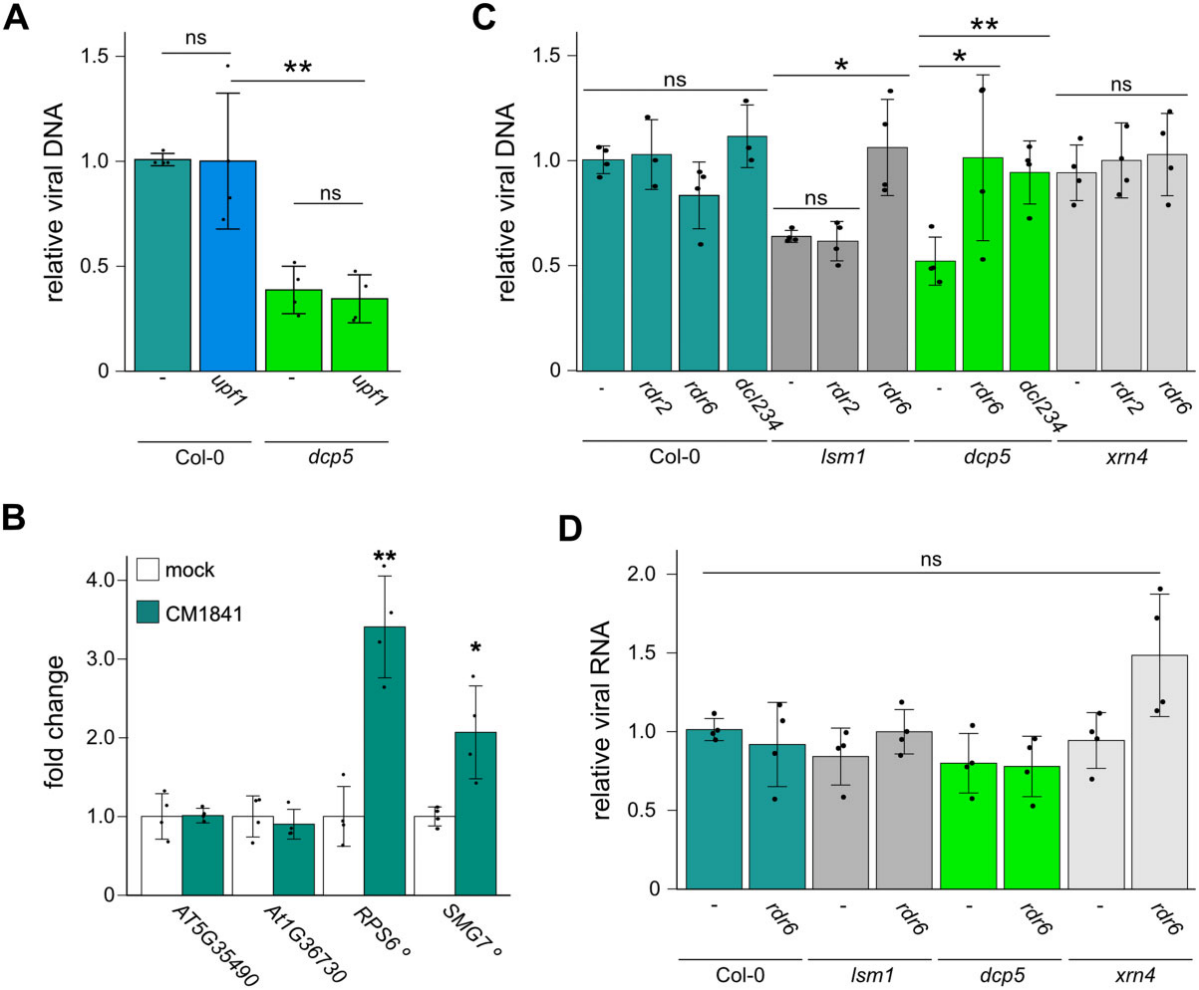
CaMV disease is rescued in combinatorial mutants with RNA silencing, but not NMD. A, Viral DNA accumulation in systemic leaves at 21 dpi in the indicated genotypes, determined by qRT-PCR. Values represent means ± sd (*n* = 4) relative to Col-0 plants and normalized to *18S* ribosomal DNA as the internal reference. B, Relative expression of NMD targets in mock and CM1841-infected rosettes 21 dpi determined by qRT-PCR. Values represent means ± sd (*n* = 4) relative to Col-0 plants and normalized to *PP2a* as the internal reference. Open circles indicate the two PTC-containing transcripts *RPS6* and *SMG7*. C, Viral DNA accumulation in systemic leaves at 21 dpi in the indicated genotypes, determined by qRT-PCR. Values represent means ± sd (*n* = 4) relative to Col-0 plants and normalized to *18S* ribosomal DNA as the internal reference. D, Viral *35s* RNA accumulation in systemically infected rosettes of the indicated genotypes at 21 dpi relative to Col-0, determined by qRT-PCR. Values represent means ± sd (*n* = 4) relative to Col-0 plants and normalized to *PP2a* as the internal reference. Statistical significance was calculated by two-sided Student’s *t* tests (*P ≤ 0.05; **P ≤ 0.01; ns, no significant difference.) for (A–D). All infection experiments were replicated at least 3 times independently.

Since PBs are at the heart of RNA triage and a hub for major RNA surveillance mechanisms, we examined whether the reduced CaMV accumulation is dependent on NMD surveillance or mediated through the RNA silencing machinery. To this end, we characterized viral infections in combinatorial mutants. CaMV titers were not affected in the previously described NMD-regulator mutant *upf1-5* (Chicois et al., 2018; Figure 5A), although the plants showed a higher relative fresh weight compared to Col-0, which is similar to *dcp5* (Supplemental Figure S7, A and B). The double mutant *dcp5 upf1* showed the same titer defect as the *dcp5* single mutant, showing that this reduction is independent of UPF1-triggered NMD (Figure 5A). A previous study found that overexpression of CaMV P6 protein relieved the suppression of several NMD targets containing different NMD marks, including premature termination codons (PTCs) and long upstream open reading frames (uORFs) (Lukhovitskaya and Ryabova, 2019). During CaMV infection, however, we only detected de-repression of PTC-carrying targets *SMG7* and *RPS6*, but not uORF-containing genes, suggesting that CaMV specifically represses PTC-triggered NMD (Figure 5B), possibly to protect against the numerous PTCs present in polycistronic viral RNA. A comparison of transcript levels in infected tissues between Col-0, *dcp5*, and *upf1* revealed that the transcription profiles of NMD targets in *dcp5* are more similar to those of Col-0 than *upf1*, uncoupling NMD regulation during CaMV infection from DCP5 functions (Supplemental Figure S8A).

Because RQC mutants are generally prone to initiate RNA silencing against highly expressed RNAs such as transgenes and viral RNAs as well as endogenous genes (Liu and Chen, 2016), we tested whether the observed viral repression in mutants affected in PB formation was mediated by the RNA silencing machinery by establishing higher-order mutants of *lsm1*, *dcp5*, and *xrn4* with *rdr2*, *rdr6* and *dcl2 dcl3 dcl4 (dcl234*; Allen et al., 2004; Xie et al., 2004; Deleris et al., 2006). These mutants, as well as their parental lines, were infected with CM1841, and virus disease was analyzed at 21 dpi. The *rdr2*, *rdr6* and *dcl234* alleles exhibited comparable fresh weight loss to Col-0 during CM1841 infection (Supplemental Figure S7D), and it is noteworthy that the RNA silencing mutants did not reverse the developmental phenotypes of *lsm1* and *dcp5* (Supplemental Figure S7C). Nonetheless, *rdr6* and *dcl234*, but not *rdr2*, rescued viral DNA accumulation in the *lsm1* and *dcp5* backgrounds while remaining at Col-0 levels in the *rdr2*, *rdr6*, and *dcl234* as well as *xrn4 rdr6* and *xrn4 rdr2* mutants (Figure 5C). The finding that comparable levels of CaMV RNA accumulated in all single and combinatorial mutants excludes the possibility that overcompensation via increased RNA content is the source of viral DNA rescue (Figure 5D). Importantly, it strengthens the notion that lower accumulation of CaMV DNA in *lsm1* and *dcp5* is a posttranscriptional effect.

### sRNA accumulation against CaMV, tasiRNA suppression, and TRV-induced gene silencing remain intact in *dcp5* and *lsm1*

**Figure 6:**
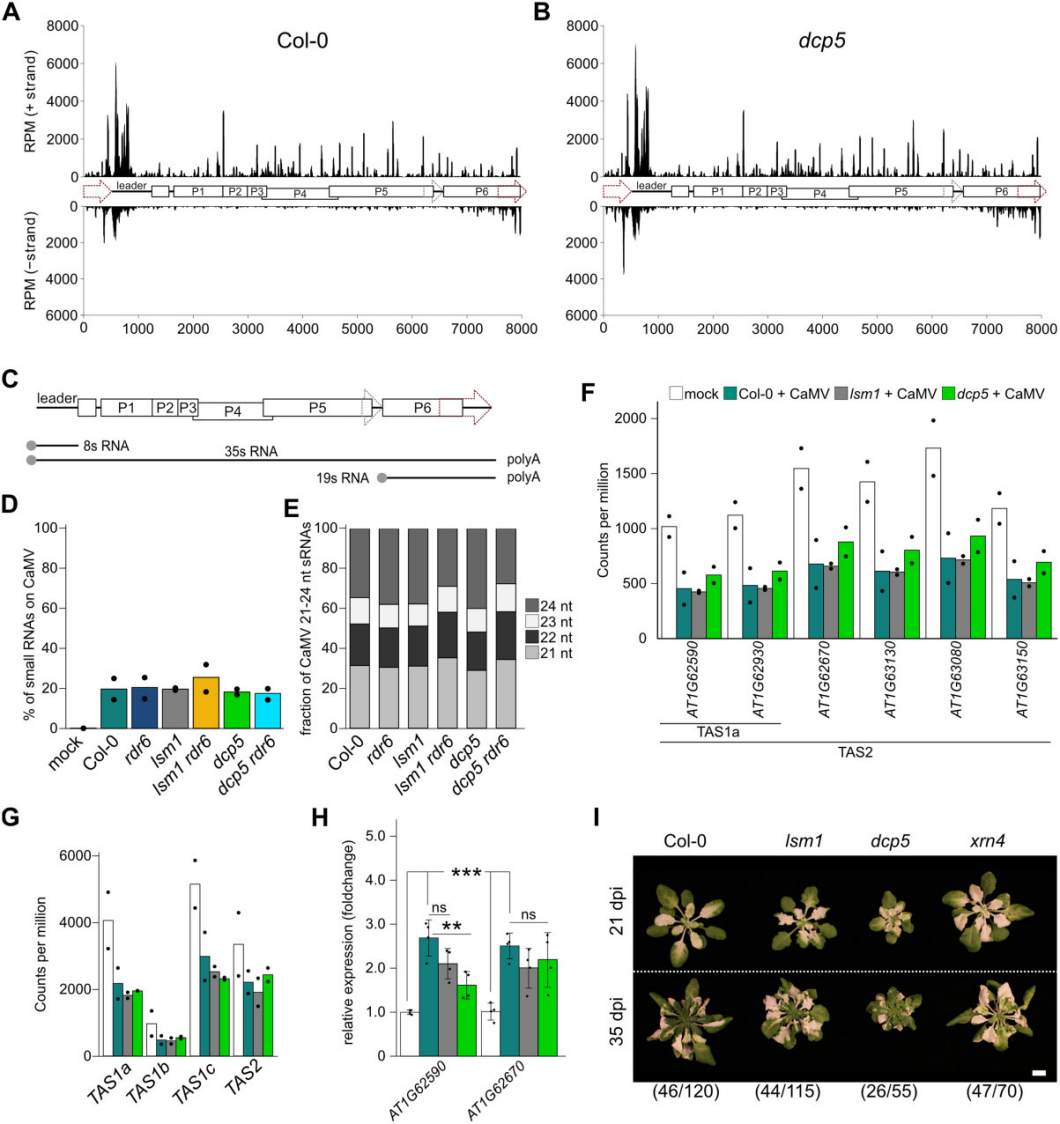
sRNA profiles on CaMV are not altered in PB and combinatorial mutants. A, Coverage plot of 24–25 nt sRNA profiles along the 8,031-bp viral genome in Col-0 21 dpi with CMI1841. The starting position was set to the beginning of the *35s* promoter (genomic position 7090). Genomic features are annotated as depicted in (C). B, Coverage plot of 24- to 5-nt sRNAs along the viral genome in *dcp5* 21 dpi with CMI1841. C, Schematic depiction of the CaMV genome. ORFs are indicated by boxes, the 19s and *35s* promotors by dashed arrows. Viral RNAs resulting from PolII transcription are depicted below the genome. D, Percent of viral sRNAs found in samples sequenced from rosette tissue 21 dpi with CMI1841 in the indicated genotypes. Bars represent the average of two biological replicates. Dots indicate single replicates. E, Fractions of 21- to 24-nt viral sRNAs in the indicated genotypes 21 dpi with CMI1841. Bars represent the average of two biological replicates. F, Normalized sRNA counts on tasiRNA target loci in the indicated genotypes. tasiRNA generating loci are depicted below the graph. Bars represent the average of two biological replicates. Dots indicate single replicates. G, Normalized sRNA counts on *TAS1a,b,c* and *TAS2* loci in the indicated genotypes. Bars represent the average of two biological replicates. Dots indicate single replicates. H, Expression of two tasiRNA targets at 21 dpi in Col-0, *lsm1*, and *dcp5* relative to mock-infected Col-0 (*n* = 4). Statistical significance was calculated by two-sided Student’s *t* tests (**P ≤ 0.01; ***P ≤ 0.001). The experiment was repeated 3 times from independent infections. I, Representative images of VIGS phenotype in the indicated genotypes at 21 and 35 dpi with TRV-PDS (scale bar = 1 cm). Numbers indicate plants showing the phenotype/total number of plants scored.

RNA silencing is frequently activated in RQC mutants and involves the biogenesis of small RNAs (sRNAs) against endogenous targets (Martinez de Alba et al., 2015). To determine whether viral sRNAs profiles and amounts were altered in *lsm1* and *dcp5* in an RDR6-dependent manner, we analyzed sRNAs in infected Col-0, *rdr6*, *lsm1, lsm1 rdr6, dcp5*, and *dcp5 rdr6* using sRNA-sequencing*.* We produced libraries from rosette samples at 21 dpi in duplicates and mapped 18–26 nucleotide (nt) reads to the TAIR10 Arabidopsis reference genome and against the CaMV genome (GenBank V00140.1). In agreement with previous observations, most viral sRNAs mapped against the highly abundant noncoding *8s* RNA (Figure 6, A and C). The percentage of sRNAs mapping to the CaMV sequence compared to sRNAs mapping against the TAIR10 genome was consistently at ∼20% (Figure 6D), with a similar size distribution (Figure 6E) as well as position and abundance across the viral genome in all genotypes and replicates (Figure 6, A–C; Supplemental Figure S9; Supplemental Data Set 1). This confirms that sRNAs mapping against the viral genome are generated independently of RDR6 and without synergistic effects in the double mutants. Hence, impairing LSM1 or DCP5 function does not have any major effects on the quantity, quality, or position of CaMV-related sRNAs.

Pathogenic plant viruses have commonly evolved viral suppressors of RNA silencing (VSRs) to counteract RNA silencing. For CaMV, the VSR protein P6 inhibits the generation of secondary RDR6-dependent trans-acting siRNAs (tasiRNA; Shivaprasad et al., 2008). To assess whether CaMV-dependent tasiRNA suppression is compromised in *lsm1* and *dcp5* mutants as a sign of a dysfunctional VSR, we counted the reads generated from the three *TAS1* and the *TAS2* loci, as well as selected tasiRNA target genes (minimal average count in Col-0 mock >1,000 reads per million [RPM]) in noninfected Col-0 and infected Col-0, *lsm1*, and *dcp5*. CaMV infection led to a decrease in sRNA counts on *TAS*-loci and tasiRNA targets (Figure 6, F and G). The reduction in sRNA occupancy was consistent in *lsm1* and *dcp5*, suggesting that P6-mediated repression of RDR6-dependent tasiRNA generation is functional in these backgrounds. Furthermore, equal increases in the transcript levels of two tested tasiRNA target genes occurred during CaMV infection (Figure 6H), further supporting that CaMV-dependent *TAS* suppression and de-repression of tasiRNA target genes are functional in *lsm1* and *dcp5*. A recent study found little difference in the sRNA profiles of *lsm1* compared to Col-0 during undisturbed growth (Krzyszton and Kufel, 2022). Whether certain endogenous sRNAs apart from tasiRNAs are misregulated in *lsm1* and *dcp5* during CaMV infection will be studied in detail in the future.

To test whether *lsm1* and *dcp5* have a general activation of virus-induced gene silencing (VIGS), as reported for other RQC mutants, we used the tobacco rattle virus-*Phytoene Desaturase* (TRV-PDS) system, which leads to leaf whitening through VIGS of *PDS* (Liu et al., 2002). We did not detect increased whitening, delayed recovery, or a higher number of symptomatic plants for *lsm1* and *dcp5*, whereas *xrn4* showed a clearly enhanced VIGS phenotype (Figure 6I). Previously, both *xrn4* and the hypomorphic *DCP2* mutant *increased transgene silencing 1* were shown to have enhanced TRV-PDS-induced VIGS (Ma et al., 2019), which was linked to higher silencing activity in these RQC-impaired backgrounds. Our results suggest that *lsm1* and *dcp5* plants do not have the same level of hyper-activated RNA silencing as the other mutants.

Taken together, *lsm1* and *dcp5* mutants (1) do not show altered viral sRNA quantities or profiles (2), do not show elevated VIGS, and (3) do not compromise the capacity of the viral silencing suppressor P6 to target the RDR6-dependent tasiRNA pathway, despite full rescue of virus DNA and protein accumulation by *rdr6* in double mutants. Importantly, based on the unaltered viral RNA levels and decay rates in *lsm1*, we propose that the RDR6-dependent suppression in the *lsm1* and *dcp5* backgrounds does not involve viral RNA degradation or transcriptional silencing.

### LSM1 and DCP5 counter RDR6-dependent translational repression of viral RNA

**Figure 7:**
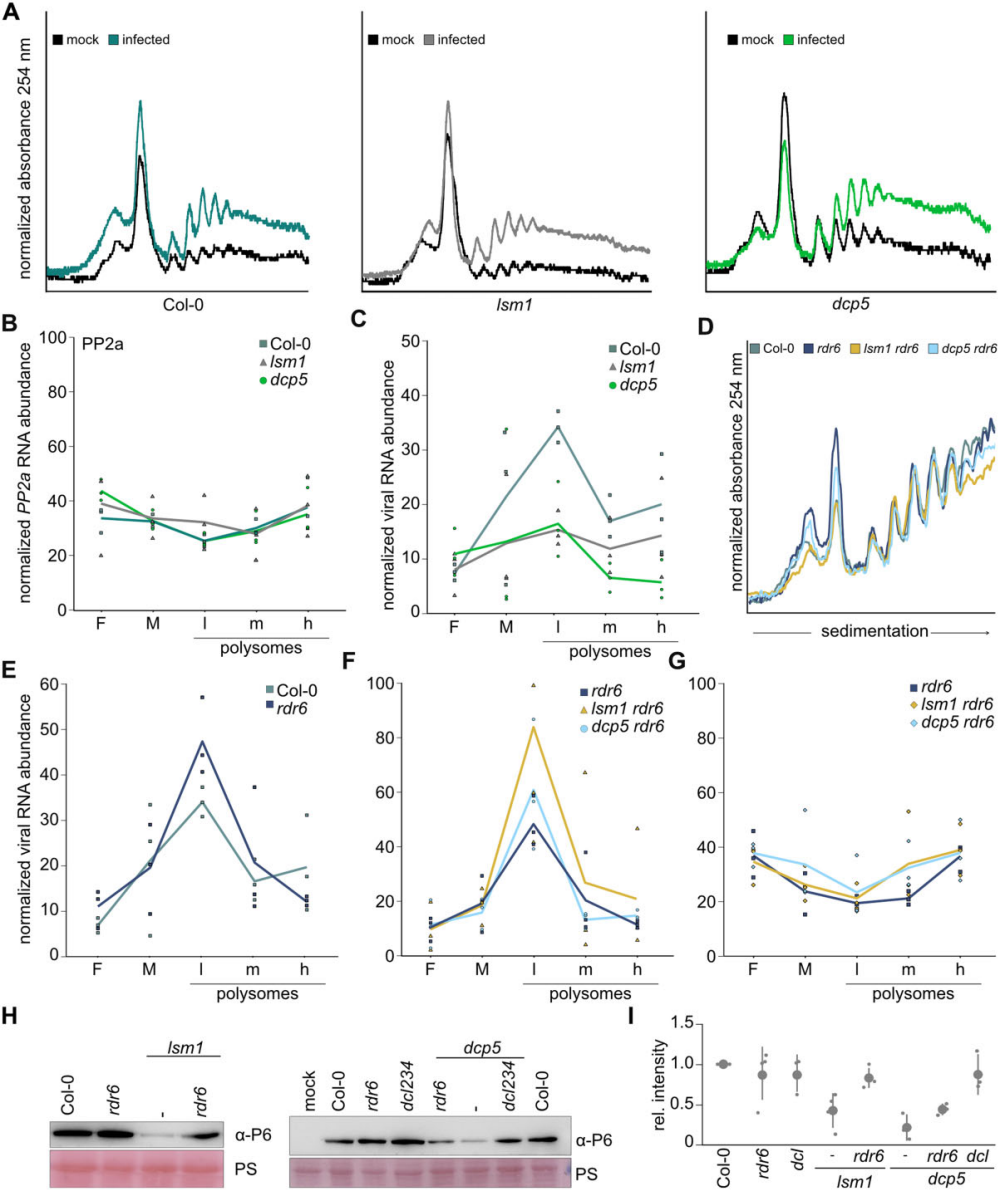
Ribosome association of viral RNA is reduced in *lsm1* and *dcp5*. A, Polysome Profiles of Col-0, *lsm1* and *dcp5* at 21-dpi CaMV (strain CM1841) infection. RNA samples were collected from unbound RNA, as well as along the gradient of ribosome-bound RNA. B, *PP2a* RNA abundance in collected samples in the indicated genotypes. The experiment was performed 3 times using material from independent infections. Fractionated RNA was normalized to *SAND* and depicted as fractions of total ribosome-bound RNA. Solid lines represent the average of biological replicates, characters represent single experiments of the indicated genotypes. Measured fractions represent free (F), monosome (M), light (l), medium (m), and heavy (h) polysome-associated RNA. C, *35s* RNA abundance in collected samples in the indicated genotypes. The experiment was performed as described in (B). D, Polysome Profiles of Col-0, *rdr6*, *lsm1 rdr6*, and *dcp5 rdr6* at 21 dpi CaMV infection. RNA samples were collected from unbound RNA, as well as along the gradient of ribosome-bound RNA. E and F, RNA abundance in collected samples measured for viral *35s* RNA in the indicated genotypes. The experiment was performed as described in (B). G, *PP2a* RNA abundance in collected samples of the indicated genotypes. The experiment was performed as described in (B). H, Immunoblot analysis of CaMV P6 protein in systemic leaves of the indicated genotypes. Total protein samples were extracted at 21 dpi and probed with anti-P6. Ponceau S (PS) staining served as a loading control. I, Quantification of signal intensity of the immunoblots in (H). Values indicate average (±sd) of protein abundance from three independent blots (for *dcp5* combinatorial mutants) or four independent blots (for *lsm1* combinatorial mutants) from independent infections quantified with ImageJ. Points represent single experiments.

The common modes of action of RNA silencing include transcriptional silencing, transcript degradation, and translational repression. After establishing the former two to be unlikely, we determined whether viral RNA translation was impaired in *lsm1* and *dcp5*. First, we performed polysomal fractionation of CaMV-infected Col-0, *lsm1* and *dcp5* samples from three independent infection experiments. Notably, CaMV-infected tissue showed markedly increased polysome abundance compared to the mock controls for both Col-0 and the mutants (Figure 7A), ruling out any global defect in translation. Fractions were collected from free and monosome-bound RNA, as well as from light, moderate, and heavy polysomes. In a first step, we confirmed the robustness of RNA content in the fractions by examining the housekeeping genes *SAND* and *PP2a* (Supplemental Figure S10A). SAND showed a stable distribution among all ribosome fractions and a decrease in abundance in the ribosome-free fraction. As control, we normalized *PP2a* expression to *SAND* expression in each fraction. PP2a abundance was comparable in the input (Supplemental Figure S10B), as well as along the gradient (Figure 7B), and had the same distribution as *SAND*. Additionally, we tested the polysome association of the four abovementioned NMD targets (Figure 5B; Supplemental Figure S8A) in two replicates of Col-0 and *dcp5*. The translation profiles for *AT5G35490* and *AT1G36730* did not differ between the genotypes, similar to their expression levels. Yet, the two PTC-carrying RNAs had a higher abundance in *dcp5* polysome fractions, strengthening the role of DCP5 as a translational repressor for endogenous targets (Supplemental Figure S10C). Importantly, these profiles validated our methodology.

We measured viral *35s* RNA in fractions from Col-0, *lsm1*, and *dcp5*. This RNA was mostly present in ribosome-bound fractions compared to free RNA, and in contrast to the tested endogenous RNAs, specifically enriched in the light polysome fraction (Figure 7C). Strikingly, the viral RNA content in ribosome-bound fractions was reduced in *lsm1* and *dcp5*, despite comparable RNA content in the input samples (Figures 7C and 5D). In accordance with the ELISA and immunoblotting results (Figure 3, E and F), the reduced ribosome association of viral RNA in the *lsm1* and *dcp5* mutants indicates that lower translation levels and not protein degradation are responsible for the decreased amounts of viral protein in these genotypes.

Finally, to confirm the notion that the rescue of viral DNA by *rdr6* is directly linked to translational efficiency, we performed polysome fractionations for the *rdr6*, *lsm1 rdr6*, and *dcp5 rdr6* mutants. The global polysome profiles were comparable among genotypes during infection (Figure 7D), and *rdr6* alone did not show an altered polysome distribution of viral RNA compared to Col-0 (Figure 7E). Intriguingly, the viral RNA in the *lsm1 rdr6* and *dcp5 dr6* double mutants showed fully restored polysome associations compared to their respective single mutants (Figure 7F), while *PP2a* remained unaffected in all tested genotypes (Figure 7G; Supplemental Figure S10B). Immunoblot analysis against viral P6 protein confirmed restoration of viral protein accumulation in the combinatorial mutants (Figure 7, H and I). Together, our results indicate that the defect in viral protein production in the *lsm1* and *dcp5* mutants is mediated through the cytoplasmic PTGS pathway governed by RDR6. In the *lsm1* or *dcp5* background, RDR6 promotes translational repression of viral RNA independently of sRNA abundance. This establishes the PB components LSM1 and DCP5 as antagonists to RNA silencing during CaMV infection and a shield to help the virus circumvent translational repression by the antiviral RNA silencing machinery.

## Discussion

Animal viruses are commonly challenged with a global shut-down of translation as part of an antiviral defense response (Walsh et al., 2013). In plants, this has so far only been observed for geminiviruses (Zorzatto et al., 2015), and in general, plant virus infections do not induce evident effects on global translation levels (Ma et al., 2015; Meteignier et al., 2016; Li et al., 2019). CaMV is exceptional, as it causes a substantial increase in polysome levels indicative of hyperactivated translation in turnips (*Brassica rapa* ssp. *rapa*) (Park et al., 2001) and Arabidopsis (this study). Translation of CaMV’s polycistronic *35s* RNA is a complex process, including mechanisms of leaky scanning and transactivation (Pooggin and Ryabova, 2018). The viral transactivation factor P6 is essential for the translation of downstream ORFs in *35s* RNA (Bonneville et al., 1989) through its interaction with a multitude of translation-associated proteins, including the translation initiation factor eIF3g, components of the large ribosomal subunit, the reinitiation supporting protein complex, and the TOR kinase (Park et al., 2004; Schepetilnikov et al., 2011).

In this study, we identified PB components as important factors that support CaMV infection via viral RNA translation, being in sharp contrast to their established function as selective repressors of endogenous mRNA translation (Brodersen et al., 2008; Xu and Chua, 2009; Jang et al., 2019). There are only a few reports identifying canonical PBs and their components as regulators of plant viral infections. Carbon Catabolite Repression 4 facilitates *Barley yellow striate mosaic virus* replication in barley (Zhang et al., 2020), *Cabbage leaf curl virus* induces RNA decay rates in PBs to reduce antiviral silencing (Ye et al., 2015), VCS supports *Potato virus A* (PVA) infection (Hafrén et al., 2015; De et al., 2020), and LSM1 strengthens *Turnip mosaic virus* infection (Zuo et al., 2022), which is in turn compromised by the overexpression of several PB components (Li and Wang, 2018). The similarities between CaMV and the fundamentally different positive-stranded RNA virus PVA are striking, as VCS promotes PVA translation in a manner closely associated with the RNA silencing pathway (Hafrén et al., 2015; De et al., 2020). Thus, it seems that plant viruses could more commonly exploit this pathway for translational targeting of their RNAs. However, as PB components were also found to limit plant viruses (Li and Wang, 2018), this interaction is more complex, and plant viruses probably evolved individually to cope with the many PB-associated functions, including more general plant innate immune responses (Chantarachot et al., 2020).

**Figure 8:**
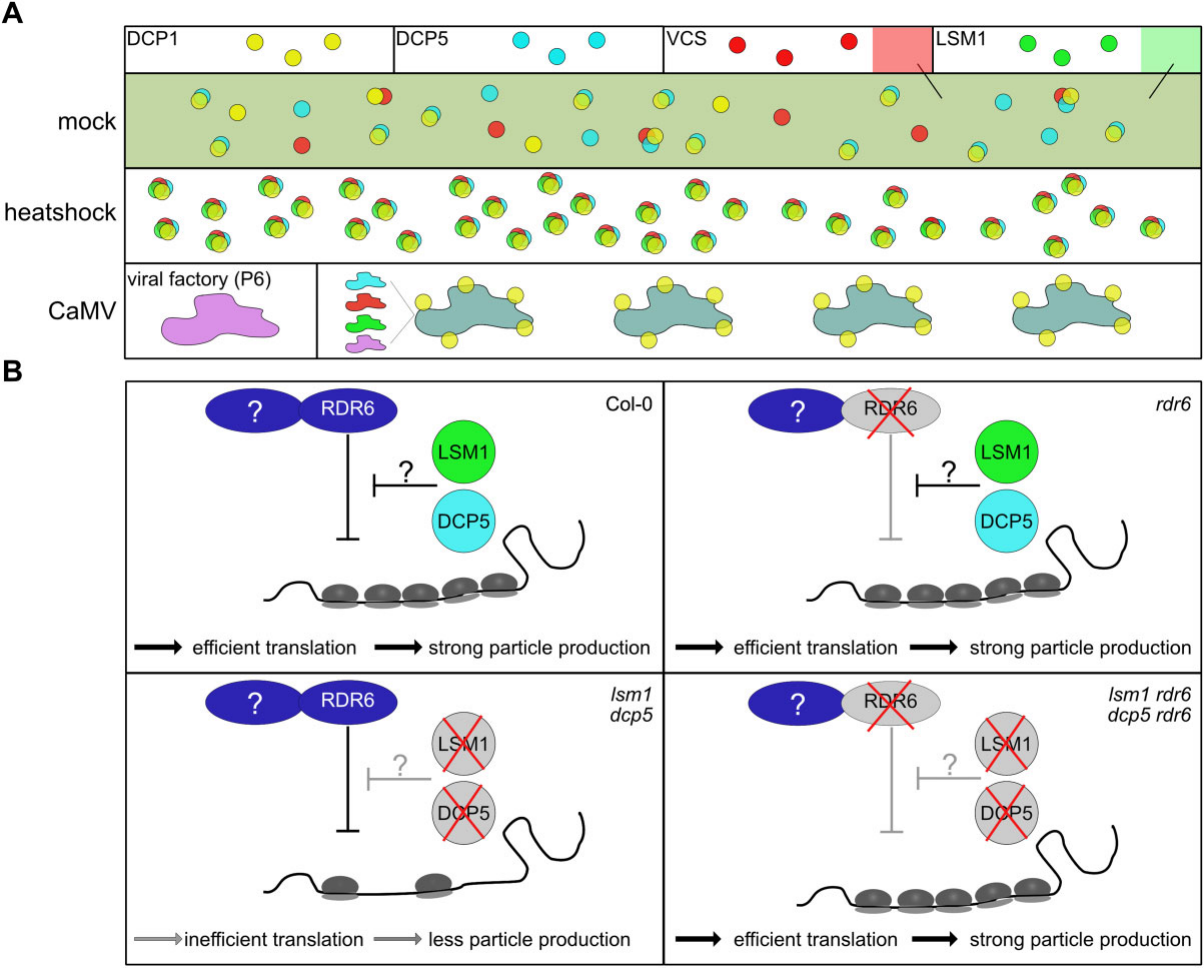
The role of LSM1 and DCP5 during CaMV infection. A, During undisturbed plant growth, PB components DCP1, DCP5, and VCS form foci that can be distinct for each protein or contain higher-order complexes of two or all three proteins, while LSM1 remains soluble in the cytoplasm. Upon heat stress, all four components assemble into higher-order complexes. CaMV produces viral factories in the cytoplasm that are sites of viral translation and replication. DCP5, VCS, and LSM1 are localized to these viral factories throughout the infection, while DCP1-marked foci assemble around, but not within viral factories. B, LSM1 and DCP5 aid viral translation by shielding the viral RNA from the repressive functions of RDR6 and possibly other proteins. Upon deletion of either DCP5 or LSM1, viral translation is impaired, leading to reduced particle production. While viral translation is not altered in the single *rdr6* mutant, it is rescued in *lsm1* and *dcp5* upon the additional deletion of RDR6, restoring the production of viral articles.

PB components are involved in several different RNA surveillance processes, including decapping, NMD, and RNA silencing, which all play major roles in translational regulation through direct degradation but also translational repression of endogenous mRNA targets (Brodersen et al., 2008; Isken et al., 2008; Lanet et al., 2009; Xu and Chua, 2009; Jang et al., 2019; Wu et al., 2020; Hung and Slotkin, 2021; Iwakawa et al., 2021). When using four established PB marker proteins, we found distinct localization patterns under nonstress conditions, and the co-assembly of VCS, LSM1a, DCP5 and DCP1 into granules after HS (Figure 8A). Our results thus support the notion that stress-induced PBs contain the higher-order decapping complex, in accordance with previous findings (Xu and Chua, 2012; Motomura et al., 2015; Perea-Resa et al., 2016), while the constitutive microscopic foci of DCP1, DCP5, and VCS are unlikely to have prominent decapping activity and may instead serve other functions, including the storage of translationally repressed RNAs (Hubstenberger et al., 2017; Courel et al., 2019). Three out of four known PB decapping components localized to VFs (Figure 8A), giving rise to the hypothesis that the mRNA decapping machinery localized here to promote viral RNA decay. However, we found that both viral RNA stability and its capping levels where unaltered in the *lsm1* knockout mutant, unlike the situation for the previously established endogenous decapping target *AT4G32020*. Indeed, mRNA degradation and translational repression are selective (Xu and Chua, 2009; Tani et al., 2012; Hubstenberger et al., 2017; Sorenson et al., 2018; Jang et al., 2019), and together with our finding that VFs lack the essential decapping activator DCP1, this function is unlikely to be associated with VFs.

The polycistronic viral *35s* RNA contains several potential triggers for RQC mechanisms, including PTCs, a large stem–loop, and extremely high expression levels. PTCs trigger degradation through NMD (Peltz et al., 1993) and in plants, this pathway was shown to suppress infections of PTC-carrying RNA viruses (Garcia et al., 2014). The primary reasons for addressing the NMD regulator UPF1 in this study is its largely shared protein interactome with DCP5 (Chicois et al., 2018), the general coupling of NMD with PBs (Lejeune et al., 2003; Raxwal et al., 2020), and the proposed capacity of P6 to suppress NMD via a direct interaction with VCS (Lukhovitskaya and Ryabova, 2019). However, CaMV showed UPF1-independent accumulation in both the *upf1* and *dcp5 upf1* mutants, disconnecting the NMD pathway from the pro-viral function of DCP5. Furthermore, endogenous targets of NMD decay were stabilized during infection in a DCP5-independent manner, suggesting that CaMV suppresses the NMD pathway irrespective of this PB component. Intriguingly, while transcript accumulation of the selected endogenous NMD targets occurred irrespective of DCP5 during CaMV infection, two targets had increased polysome association in *dcp5*, suggesting that these targets are under PB translational repression, unlike CaMV.

Our results suggest that the RNA silencing component RDR6, likely in conjunction with DCLs, mediates translational repression of the viral RNA in *dcp5* and *lsm1*. A link between RDR6-dependent RNA silencing and PBs was initially established in plants from forward genetic screens of induced transgene silencing, identifying both *xrn4* (Gazzani et al., 2004) and *dcp2* (Thran et al., 2012). Subsequently, endogenous genes were also shown to become targets of RDR6-dependent sRNA biogenesis in more severe seedling-lethal decapping mutants (Martinez de Alba et al., 2015). Based on these and other findings, the current model postulates that when the capacity of mRNA decay is exceeded, for example, overloaded with substrate or functionally compromised, decay substrates leak into the RDR6/DCL2/DCL4 pathway for sRNA biogenesis and subsequent RNA silencing processes (Liu and Chen, 2016). Even though CaMV infection is analogously compromised by RDR6 in *lsm1* and *dcp5*, we obtained numerous lines of evidence that this phenomenon differs from the above-described canonical model: (1) There were no evident changes in viral sRNA quantity or profiles in the mutants; (2) viral RNA levels remained largely unaffected, unlike transgenes, which are degraded and transcriptionally silenced; (3) *xrn4* did not weaken CaMV infection; and (4) viral RNA does not qualify as a substrate, as it showed no detectable levels of LSM1-dependent decapping and decay.

Plant viruses have frequently evolved means to suppress antiviral RNA silencing (Morel et al., 2002; Garcia-Ruiz et al., 2010; Garcia et al., 2014; Csorba et al., 2015). This includes CaMV, which is normally insensitive to DCL- and RDR6-dependent RNA silencing (Blevins et al., 2011), relying on at least two different strategies (Hohn, 2015). First, viral P6 suppresses the DRB4/DCL4 node of PTGS (Haas et al., 2008; Shivaprasad et al., 2008), a process that seems to also function in *lsm1* and *dcp5* judging from the comparably reduced levels of tasiRNAs along de-repression of their targets during infection. Second, these mutants show similar massive accumulation of viral sRNAs derived from *8s*, which are thought to constitute an important part of suppression by saturating and decoying the RNA silencing machinery with ineffective sRNAs (Blevins et al., 2011). Thus, both RNA silencing suppression strategies of CaMV appear to operate normally in the *lsm1* and *dcp5* mutants, prompting us to propose that PB dysfunction exposes the virus to a new, otherwise avoided RNA silencing-based translational repression mechanism (Figure 8B).

Having established a fundamentally novel framework around the balance between PB components and the RNA silencing machinery in CaMV RNA translation, the detailed mechanism becomes intriguing and requires further attention. RNA silencing involving RDR6, SGS3, and specifically DCL2-dependent 22-nt sRNAs were recently proposed to act together in translational repression during stress adaption and defense against transposons (Wu et al., 2020; Iwakawa et al., 2021; Kim et al., 2021). These studies identified abundant sRNA accumulation as part of the process, while CaMV sRNAs levels and profiles remained unaltered during translational repression in *lsm1* and *dcp5*. This is not necessarily a discrepancy, because all major size classes of viral sRNAs are already highly abundant in wild-type plants and likely sufficient to drive the response with increased efficiency. Intriguingly, both RDR6 and SGS3 are well-established components of siRNA bodies (Jouannet et al., 2012; Kim et al., 2021), and the concept of substrate channeling and competition between PBs and siRNA bodies has been proposed (Jouannet et al., 2012), along with the general connection between RQC mutants and RDR6 (Liu and Chen, 2016). In summary, we propose that the association of PB components with CaMV VFs reduces viral RNA exposure, thereby evading translational repression by the RDR6 pathway (Figure 8B).

## Material and methods

### Plant material and growth conditions

All mutants used in this study were in the *Arabidopsis thaliana* accession Columbia-0 (Col-0) background, which was used as a control for all experiments (Supplemental Data Set 2). Mutants were checked for homozygosity using the primers described in Supplemental Data Set 3. Arabidopsis and *N.* *benthamiana* plants were grown in walk-in chambers under standard long-day conditions (120 mE,16-h light/8-h dark cycle) at 22°C day temperature (20°C night temperature) and 65% relative humidity for crossing, propagation, and transient expression assays. For infection experiments, plants were grown under short-day conditions (120 mE, 10-h light/14-h dark cycle) at 22°C day temperature (19°C night temperature) and 65% relative humidity. Light spectra in both conditions ranged from 400 to 720 nm.

### Plasmid construction, generation of transgenic lines, and transient expression

The pENTRY clone containing the full-length Cabb B-JI P6 coding sequence (Hafren et al., 2017) was cloned into the pGWB654 or pGWB554 vector under the control of the *35s* promoter (Nakagawa et al., 2007). Expressor lines were generated for this study by the floral dip method (Clough and Bent, 1998); all lines and constructs are listed in Supplemental Data Set 2. The coding sequences of *DCP1, DCP5, VCS*, and *LSM1a* were amplified from Col-0 plants (primers listed in Supplemental Data Set 4), cloned into pENTR/D-TOPO, and recombined in the pUBN/C-dest vector system for GFP fusions (Grefen et al., 2010). To establish PB double marker lines, *DCP1* and *LSM1* were cloned into the pUBC-mRFP vector and introduced into the GFP-VCS background by the floral dip method (Grefen et al., 2010). For transient expression, all coding sequences were cloned into pUBC for GFP fusions and pGWB654 for mRFP fusions. *Nicotiana benthamiana* leaves were infiltrated with resuspended Agrobacterium strain C58C1 cells (optical density (OD) 0.2, 10-mM MgCl_2_, 10-mM MES pH 5.6, 150-µM acetosyringone) and the constructs analyzed after 48 h.

### Virus inoculation and quantification

Arabidopsis plants were infected with CaMV or TRV 18 days after germination. The first true leaves were infiltrated with *Agrobacterium tumefaciens* strain C58C1 carrying CaMV strain CM1841 or TRV RNA1 and 2 (Liu et al., 2002) (OD 0.15) or mechanically rubbed with Cabb B-JI particles that were purified from turnip leaves and resuspended in carborundum-supplemented phosphate buffer (Martinière et al., 2009). Rosettes were harvested 21 dpi in four biological replicates, each containing two to three individual plants from which inoculated leaves were removed. All experiments were repeated from independent infections, each containing three to four biological replicates. For CaMV DNA quantification, 100 mg pulverized frozen leaf material was resuspended in 300 µL 100-mM Tris buffer (pH 7.5), supplemented with 2% sodium dodecyl sulfate (SDS) and treated with 0.2-mg/mL Proteinase K. Total DNA was precipitated with isopropanol 1:1 (v:v), and viral DNA levels were determined by quantitative Real-Time polymerase chain reaction (qRT-PCR) and normalized to *18S* ribosomal DNA (Hafren et al., 2017). RNA extraction from rosette tissue was performed with a Qiagen RNeasy kit and on-column DNase I digestion according to the manufacturer’s protocol. About 500 ng of total RNA was used for first-strand cDNA synthesis with a Maxima First Strand cDNA Synthesis Kit (Thermo Fisher Scientific Waltham, MA, USA]). qRT-PCR analysis was performed with Maxima SYBR Green/Fluorescein qRT-PCR Master Mix (Thermo Fisher Scientific) using the CFX Connect Real-Time PCR detection system (Bio-Rad, Hercules, CA, USA) with gene-specific primers (Supplemental Data Set 5). Viral transcripts were normalized to *PP2a* (*AT1G69960*) and expression levels determined as described by Livak and Schmittgen (2001).

#### Immunoblot analysis

Proteins were extracted from frozen rosette tissue in 100-mM Tris buffer (pH 7.5) supplemented with 2% SDS. Samples were incubated at 95°C for 5 min in 1× Laemmli sample buffer and cleared by centrifugation. The protein extracts were separated by SDS–PAGE, transferred to polyvinylidene difluoride (PVDF) membranes (Amersham, GE Healthcare, Amersham, UK), and blocked with 8% (w:v) skimmed milk in 1× PBS, supplemented with 0.05% Tween-20. Blots were incubated with 1:2,000 diluted primary antibodies α-P3 (Drucker et al., 2002), α-P4 (Champagne et al., 2004), α-P6 (Schoelz et al., 1991), or α-GFP (Santa Cruz Biotechnology, Dallas, TX, USA; sc-9996) before the subsequent addition of secondary horseradish peroxidase-conjugated antibodies (1:20,000; NA934 and NA931, Amersham, GE Healthcare). The immunoreaction was developed using an ECL Prime kit (Amersham, GE Healthcare) and was detected in the LAS-3000 Luminescent Image Analyzer (Fujifilm, Tokyo, Japan). Quantification of band intensities was performed on blots using ImageJ 1.48v (Schneider et al., 2012). Band intensities were normalized to Ponceau S stain. An ELISA was performed for three independent experiments, with 100-mg infected plant material in 1 mL (w/v) 8M Urea buffer. Samples were incubated on high-binding ELISA plates for 6 h at 37°C before blocking in 5% skimmed milk. Primary antibodies were added at 1:500 dilution overnight and secondary antibodies at 1:1,000 dilution for 3 h at 37°C. Absorbance was measured at 405 nm from 30 to 120 min after the addition of Substrate buffer (PNPP; Thermo Fisher).

#### Cap-dependent immunoprecipitation and XRN1 digestion

Immunoprecipitation of 7-methylguanosine (m^7^G)-capped RNA was performed as described by Golisz et al. (2013). Anti-m^7^G-Cap mAb (clone 150-15) was purchased from MBL International Corporation. For exonucleolytic digestion, total RNA was extracted from rosettes 21 dpi and incubated at 37°C with 1U XRN1 enzyme (Thermo Fischer) or in reaction buffer (mock) (Roux et al., 2015). cDNA synthesis and qRT-PCR were performed as described in the previous section. Transcript levels were normalized to *eIF4a* (*AT3G13920*; Perea-Resa et al., 2012; Roux et al., 2015).

#### RNA half-life measurement

Rosettes of CaMV-infected plants (21 dpi) were vacuum infiltrated with 1-mM Cordycepin (Sigma-Aldrich, St. Louis, MO, USA) in buffer (1-mM PIPES, pH 6.25, 1-mM sodium citrate, 1-mM KCl, 15-mM sucrose) and placed in a damp chamber. Two plants were harvested per sample corresponding to 0, 15, 30, 60, and 120 min after transcriptional inhibition. Total RNA was extracted using TRIzol reagent, followed by cDNA synthesis and qRT-PCR as described in the previous section. RNA levels were normalized to *eIF4a* (*AT3G13920*).

#### Preparation, sequencing, and analysis of sRNA libraries

sRNA libraries were prepared from 500-ng total RNA with a NEBNext Multiplex Small RNA Library Prep Set for Illumina (E7300; New England Biolabs, Ipswich, MA, USA) according to the manufacturer’s protocol. The amplification step was set to 12 cycles, and amplified libraries were cleaned using SPRIselect beads (Beckman Coulter, Brea, CA, USA). Size selection was performed on a 6% polyacrylamide gel according to the manufacturer’s protocol and eluted in 10-µL TE buffer. Size range and library concentrations were confirmed using the Bioanalyzer 2100 (Agilent Systems, St Clara, CA, USA). Libraries were sequenced on an Illumina Nextseq2000 system in paired-end 50-bp mode at the SciLifeLab facility, Solna, Sweden.

For sRNA-seq analysis, reads were obtained in fastq file format from the facility. Adapters were trimmed using flexbar with the -ap ON option (version 3.5.0; (Roehr et al., 2017)) and the corresponding adaptor sequences as indicated for the NEBnext E7300 sRNA preparation kit (read1 AGATCGGAAGAGCACACGTCTGAACTCCAGTCAC, read 2 GATCGTCGGACTGTAGAACTCTGAACGTGTAGATCTCGGTGGTCGCCGTATCATT). Afterward, corresponding forward and reverse reads were combined using fastq-join (version 1.3.1; https://github.com/ExpressionAnalysis/ea-utils/blob/wiki/FastqJoin.md) and the option-v for illumina reads. Joint fastq files were size trimmed to obtain only sizes of 18–26 nt by utilizing cutadapt (version 1.9.1) (Martin, 2011) with the parameters -m 18 -M 26. Read size and quality were checked using FastQC in default mode (version 0.11.9; http://www.bioinformatics.babraham.ac.uk/projects/fastqc/). Size trimmed fastq files were further processed as described in Bente et al. (2021) using publicly available scripts (https://github.com/AlexSaraz1/paramut_bot). To create sRNA profiles along the CaMV sequence, the hygromycin phosphotransferase transgenic sequence from Bente et al. 2021 was replaced by the genomic CaMV sequence (GenBank V00140.1) by setting the start position to the beginning of the *35s* promoter. Reads were aligned to the TAIR10 genome including an extra contig containing the above-mentioned CaMV sequence. Alignment was done using Bowtie2 (version 2.3.5.1; Langmead and Salzberg, 2012) with the options -k 500 -no-unal. For read counts in the TAS genes and its targets, featureCounts (version v1.6.3; Liao et al., 2014) from the Subread package (http://subread.sourceforge.net/) was used with the options -t gene -s 1 -M on the aligned bam files (Liao et al., 2014). Graphical representation was achieved using R. For profile comparisons along the CaMV sequence, the axes were adjusted to 8,000 and 6,000 RPM on the positive and negative strands, respectively. An overview of the processed sRNA libraries is shown in Supplemental Data Set 1.

### Polysome isolation

Polysome extraction was performed based on Mustroph et al. (2009) with some modifications. Briefly, 1-mL frozen leaf powder was thawed in 8 mL of polysome extraction buffer (200-mM Tris–HCl (pH 8.0), 200-mM KCl, 35-mM MgCl_2_, 25-mM EGTA, 1-mM DTT, 1-mM phenylmethanesulfonylfluoride, 100-μg/mL CHX, 1% (vol/vol) detergent mix (20% (w/v) Brij-35, 20% (v/v) Triton X-100, 20% (v/v) Igepal CA630, and 20% Tween-20), 1% (v/v) polyoxyethylene 10 tridecyl ether), resuspended, and kept on ice for 10 min. The plant debris was removed by centrifuging at 16,000 *g* for 15 min at 4°C in a JA-25.50 rotor and Avanti

J-20 XP centrifuge (Beckman Coulter). The clear supernatant was gently poured on top of an 8-mL sucrose cushion (100-mM Tris–HCl (pH 8.0), 40-mM KCl, 20-mM MgCl_2_, 5-mM EGTA, 1-mM DTT, 100-μg/mL CHX in 60% sucrose) in a 26-mL polycarbonate tube (Beckman Coulter). After proper balancing, the samples were centrifuged at 35,000 RPM for 18 h at 4°C in a 70Ti rotor and L8-M ultracentrifuge (Beckman Coulter). The ribosome pellets were gently washed with RNase-free water and resuspended in 300 μL of resuspension buffer (100-mM Tris–HCl (pH 8.0), 40-mM KCl, 20-mM MgCl_2_, 100-μg/mL CHX). The resuspended samples were kept on ice for 30 min, followed by centrifugation at 16,000*g* at 4°C to remove any debris. The RNA content was measured for each sample using a Qubit BR RNA assay kit (Thermo Fisher Scientific). The resuspended ribosome samples were loaded on 15%–60% sucrose gradients and centrifuged at 50,000 RPM in a SW55.1 rotor and L8-M ultracentrifuge (Beckman Coulter). The gradient samples were fractionated using an ISCO absorbance detector (model # UA-5, ISCO, Lincoln, NE) to obtain fractions of ∼250 µL. The fractions were pooled before RNA extraction with TRIzol to obtain samples from free RNA as well as monosome-bound RNA and three pools of light, medium, and heavy polysome-bound RNA. RNA levels were normalized to *SAND* (*AT2G28390*) in each fraction and depicted as fractions of the sum of ribosome-bound RNA in Col-0 (for *dcp5*, *lsm1*, and *rdr6*) or *rdr6* (for *lsm1 rdr6* and *dcp5 rdr6*) to enable the comparison of total abundance on ribosomes, as well as relative abundance along the gradient, after testing for comparable RNA input.

### Confocal microscopy and treatments

Micrographs from leaf abaxial epidermal cells were taken under a Zeiss LSM 780 microscope. GFP and RFP signals were detected at 488 nm/490–552 nm and 561 nm/569–652 nm, respectively. Co-visualization was achieved through sequential scanning mode. For HS conditions, leaves were kept in water at 38°C for 30 min (1 h for LSM1-GFP) before imaging. Translational inhibition treatment was achieved by infiltrating young leaves with 200 µM CHX (Sigma-Aldrich), followed by incubation for 1 h before imaging. Images were processed with ZEN black software (Zeiss) and ImageJ version 1.53b. For quantification, Z-stacks were Brightness increased and a median filter of 2 pixels applied. Stomata were manually deleted from micrographs, and a mask was generated through thresholding. Foci were counted using the “Analyze Particles” tool.

### Data analysis and statistical methods

Statistical comparisons of two groups were performed by Student’s t test in Excel. One-way analysis of variance (ANOVA) followed by a post-hoc Tukey HSD test (α = 0.05) was performed with R version 4.0.02 and the R-package “agricolae” (version 1.3-3; https://cran.rproject.org/web/packages/agricolae/index.html). Test statistics are shown in Supplemental Data Set 6.

### Accession numbers

Sequence data from this article can be found in the EMBL/GenBank data libraries under the following accession numbers: DCP1 (AT1G08370), DCP2 (AT5G13570), LSM1a (AT1G19120), LSM1b (AT3G14080), DCP5 (AT1G26110), XRN4 (AT1G54490), VCS (AT3G13300), RDR2 (AT4G11130), RDR6 (AT3G49500), DCL2 (AT3G03300), DCL3 (AT3G43920), DCL4 (AT5G20320), UPF1 (AT5G47010), TAS1a (AT2G27400), TAS1b (AT1G50055), TAS1c (AT2G39675), TAS2 (AT2G39681), RPS6 (AT5G4670), and SMG7 (AT5G19400).

sRNA sequencing data from this study were deposited in the Gene Expression Omnibus database (https://www.ncbi.nlm.nih.gov/geo/) under accession number GSE194186, and raw data were deposited in the Sequencing Read Archive (https://www.ncbi.nlm.nih.gov/sra) under accession number SRP356192.

## Supplemental data

The following materials are available in the online version of this article.

**Supplemental Figure S1**. Description of PB markers during CaMV infection.

**Supplemental Figure S2**. PB double marker lines show co-assembly after HS and during CaMV infection.

**Supplemental Figure S3**. Co-expression of PB components with CaMV proteins in *N. benthamiana*.

**Supplemental Figure S4**. Co-localization of P6 with PB components under mock conditions.

**Supplemental Figure S5**. Viral DNA accumulation in additional PB mutants.

**Supplemental Figure S6.** *35s* RNA decay after transcriptional arrest with cordycepin.

**Supplemental Figure S7**. CaMV disease in combinatorial mutants with NMD and RNA silencing.

**Supplemental Figure S8**. Expression of NMD targets in *dcp5* and *upf1* during CaMV infection.

**Supplemental Figure S9**. sRNA profiles in *lsm1* and combinatorial mutants.

**Supplemental Figure S10**. Translational profiling during CaMV infection.

**Supplemental Data Set 1.** Total number of sRNA reads in each sample and their mapping to the 8,031-bp CaMV genome.

**Supplemental Data Set 2.** Plant material used and generated in this study.

**Supplemental Data Set 3.** DNA oligonucleotides used in this study for genotyping.

**Supplemental Data Set 4.** DNA oligonucleotides used in this study for molecular cloning.

**Supplemental Data Set 5.** DNA oligonucleotides used in this study for expression analysis.

**Supplemental Data Set 6.** ANOVA tables.

## Supplementary Material

koac132_Supplementary_Data

## Notes

G.H. and A.H. designed the experiments and wrote the manuscript. G.H. conducted the experiments, analyzed the data, and designed the figures. Polysome analysis was conducted and analyzed together with A.M. and J.H. H.B. advised on library preparation and analyzed the sRNA data together with G.H. D.G. provided the *upf1*, *upf1 dpc5*, and DCP5-GFP Arabidopsis lines. All authors edited the manuscript and approved the final version.

The author responsible for distribution of materials integral to the findings presented in this article in accordance with the policy described in the Instructions for Authors (https://academic.oup.com/plcell) is: Anders Hafrén (anders.hafren@slu.se).

## References

[koac132-B1] Allen E , Xie Z, Gustafson AM, Sung GH, Spatafora JW, Carrington JC (2004) Evolution of microRNA genes by inverted duplication of target gene sequences in Arabidopsis thaliana. Nat Genet 36: 1282–1290 15565108 10.1038/ng1478

[koac132-B2] Anderson P , Kedersha N (2009) RNA granules: post-transcriptional and epigenetic modulators of gene expression. Nat Rev Mol Cell Biol 10: 430–436 19461665 10.1038/nrm2694

[koac132-B3] Bente H , Foerster AM, Lettner N, Mittelsten Scheid O (2021) Polyploidy-associated paramutation in Arabidopsis is determined by small RNAs, temperature, and allele structure. PLoS Genet 17: e1009444 33690630 10.1371/journal.pgen.1009444 PMC7978347

[koac132-B4] Blevins T , Rajeswaran R, Aregger M, Borah BK, Schepetilnikov M, Baerlocher L, Farinelli L, Meins F Jr, Hohn T, Pooggin MM (2011) Massive production of small RNAs from a non-coding region of Cauliflower mosaic virus in plant defense and viral counter-defense. Nucleic Acids Res 39: 5003–5014 21378120 10.1093/nar/gkr119 PMC3130284

[koac132-B5] Bonneville JM , Sanfaçon H, Fütterer J, Hohn T (1989) Posttranscriptional trans-activation in cauliflower mosaic virus. Cell 59: 1135–1143 2598263 10.1016/0092-8674(89)90769-1

[koac132-B6] Branco-Price C , Kawaguchi R, Ferreira RB, Bailey-Serres J (2005) Genome-wide analysis of transcript abundance and translation in Arabidopsis seedlings subjected to oxygen deprivation. Ann Bot 96: 647–660 16081496 10.1093/aob/mci217 PMC4247032

[koac132-B7] Brodersen P , Sakvarelidze-Achard L, Bruun-Rasmussen M, Dunoyer P, Yamamoto YY, Sieburth L, Voinnet O (2008) Widespread translational inhibition by plant miRNAs and siRNAs. Science 320: 1185–1190 18483398 10.1126/science.1159151

[koac132-B8] Buchan JR , Parker R. (2009) Eukaryotic stress granules: the ins and outs of translation. Mol Cell 36: 932–941 20064460 10.1016/j.molcel.2009.11.020 PMC2813218

[koac132-B9] Champagne J , Benhamou N, Leclerc D (2004) Localization of the N-terminal domain of cauliflower mosaic virus coat protein precursor. Virology 324: 257–262 15207613 10.1016/j.virol.2004.04.014

[koac132-B10] Chantarachot T , Bailey-Serres J (2018) Polysomes, stress granules, and processing bodies: a dynamic triumvirate controlling cytoplasmic mRNA fate and function. Plant Physiol 176: 254–269 29158329 10.1104/pp.17.01468 PMC5761823

[koac132-B11] Chantarachot T , Sorenson RS, Hummel M, Ke H, Kettenburg AT, Chen D, Aiyetiwa K, Dehesh K, Eulgem T, Sieburth LE, et al (2020) DHH1/DDX6-like RNA helicases maintain ephemeral half-lives of stress-response mRNAs. Nat Plants 6: 675–685 32483330 10.1038/s41477-020-0681-8

[koac132-B12] Chicois C , Scheer H, Garcia S, Zuber H, Mutterer J, Chicher J, Hammann P, Gagliardi D, Garcia D (2018) The UPF1 interactome reveals interaction networks between RNA degradation and translation repression factors in Arabidopsis. Plant J 96: 119–132 29983000 10.1111/tpj.14022

[koac132-B13] Clough SJ , Bent AF (1998) Floral dip: a simplified method for Agrobacterium -mediated transformation of Arabidopsis thaliana. Plant J 16: 735–743 10069079 10.1046/j.1365-313x.1998.00343.x

[koac132-B14] Courel M , Clément Y, Bossevain C, Foretek D, Vidal Cruchez O, Yi Z, Bénard M, Benassy MN, Kress M, Vindry C, et al (2019) GC content shapes mRNA storage and decay in human cells. eLife 8: e49708 31855182 10.7554/eLife.49708 PMC6944446

[koac132-B15] Csorba T , Kontra L, Burgyán J (2015) viral silencing suppressors: tools forged to fine-tune host-pathogen coexistence. Virology 479–480: 85–103 10.1016/j.virol.2015.02.028 25766638

[koac132-B16] De S , Pollari M, Varjosalo M, Mäkinen K (2020) Association of host protein VARICOSE with HCPro within a multiprotein complex is crucial for RNA silencing suppression, translation, encapsidation and systemic spread of potato virus A infection. PLoS Pathog 16: e1008956 33045020 10.1371/journal.ppat.1008956 PMC7581364

[koac132-B17] Deleris A , Gallego-Bartolome J, Bao J, Kasschau KD, Carrington JC, Voinnet O (2006) Hierarchical action and inhibition of plant Dicer-like proteins in antiviral defense. Science 313: 68–71 16741077 10.1126/science.1128214

[koac132-B18] Drucker M , Froissart R, Hébrard E, Uzest M, Ravallec M, Espérandieu P, Mani JC, Pugniere M, Roquet F, Fereres A (2002) Intracellular distribution of viral gene products regulates a complex mechanism of cauliflower mosaic virus acquisition by its aphid vector. Proc Natl Acad Sci USA 99: 2422–2427 11842201 10.1073/pnas.042587799 PMC122380

[koac132-B19] Espinoza AM , Medina V, Hull R, Markham P (1991) Cauliflower mosaic virus gene II product forms distinct inclusion bodies in infected plant cells. Virology 185: 337–344 1656590 10.1016/0042-6822(91)90781-6

[koac132-B20] Garcia D , Garcia S, Voinnet O (2014) Nonsense-mediated decay serves as a general viral restriction mechanism in plants. Cell Host Microb 16: 391–402 10.1016/j.chom.2014.08.001 PMC7185767 25155460

[koac132-B21] Garcia-Ruiz H , Takeda A, Chapman EJ, Sullivan CM, Fahlgren N, Brempelis KJ, Carrington JC (2010) Arabidopsis RNA-dependent RNA polymerases and dicer-like proteins in antiviral defense and small interfering RNA biogenesis during Turnip Mosaic Virus infection. Plant Cell 22: 481–496 20190077 10.1105/tpc.109.073056 PMC2845422

[koac132-B22] Gazzani S , Lawrenson T, Woodward C, Headon D, Sablowski R (2004) A link between mRNA turnover and RNA interference in Arabidopsis. Science 306: 1046–1048 15528448 10.1126/science.1101092

[koac132-B23] Golisz A , Sikorski PJ, Kruszka K, Kufel J (2013) Arabidopsis thaliana LSM proteins function in mRNA splicing and degradation. Nucleic Acids Res 41: 6232–6249 23620288 10.1093/nar/gkt296 PMC3695525

[koac132-B24] Grefen C , Donald N, Hashimoto K, Kudla J, Schumacher K, Blatt MR (2010) A ubiquitin-10 promoter-based vector set for fluorescent protein tagging facilitates temporal stability and native protein distribution in transient and stable expression studies. Plant J 64: 355–365 20735773 10.1111/j.1365-313X.2010.04322.x

[koac132-B25] Guzikowski AR , Chen YS, Zid BM (2019) Stress-induced mRNP granules: form and function of processing bodies and stress granules. Wiley Interdiscip Rev RNA 10: e1524 30793528 10.1002/wrna.1524 PMC6500494

[koac132-B26] Haas G , Azevedo J, Moissiard G, Geldreich A, Himber C, Bureau M, Fukuhara T, Keller M, Voinnet O (2008) Nuclear import of CaMV P6 is required for infection and suppression of the RNA silencing factor DRB4. EMBO J 27: 2102–2112 18615098 10.1038/emboj.2008.129 PMC2516879

[koac132-B27] Hafrén A , Lõhmus A, Mäkinen K (2015) Formation of Potato virus A-induced RNA granules and viral translation are interrelated processes required for optimal virus accumulation. PLoS Pathog 11: e1005314 26641460 10.1371/journal.ppat.1005314 PMC4671561

[koac132-B28] Hafren A , Macia JL, Love AJ, Milner JJ, Drucker M, Hofius D (2017) Selective autophagy limits cauliflower mosaic virus infection by NBR1-mediated targeting of viral capsid protein and particles. Proc Natl Acad Sci USA 114: E2026–E2035 28223514 10.1073/pnas.1610687114 PMC5347569

[koac132-B29] Hohn T (2015) RNA based viral silencing suppression in plant pararetroviruses. Front Plant Sci 6: 398 26113850 10.3389/fpls.2015.00398 PMC4462095

[koac132-B30] Hubstenberger A , Courel M, Benard M, Souquere S, Ernoult-Lange M, Chouaib R, Yi Z, Morlot JB, Munier A, Fradet M, et al (2017) P-body purification reveals the condensation of repressed mRNA regulons. Mol Cell 68: 144–157 e5. 28965817 10.1016/j.molcel.2017.09.003

[koac132-B31] Hung YH , Slotkin RK (2021) The initiation of RNA interference (RNAi) in plants. Curr Opin Plant Biol 61: 102014 33657510 10.1016/j.pbi.2021.102014

[koac132-B32] Isken O , Kim YK, Hosoda N, Mayeur GL, Hershey JW, Maquat LE (2008) Upf1 phosphorylation triggers translational repression during nonsense-mediated mRNA decay. Cell 133: 314–327 18423202 10.1016/j.cell.2008.02.030 PMC4193665

[koac132-B33] Iwakawa HO , Lam AYW, Mine A, Fujita T, Kiyokawa K, Yoshikawa M, Takeda A, Iwasaki S, Tomari Y (2021) Ribosome stalling caused by the Argonaute-microRNA-SGS3 complex regulates the production of secondary siRNAs in plants. Cell Rep 35: 109300 34192539 10.1016/j.celrep.2021.109300

[koac132-B34] Jang GJ , Yang JY, Hsieh HL, Wu SH (2019) Processing bodies control the selective translation for optimal development of Arabidopsis young seedlings. Proc Natl Acad Sci USA 116: 6451–6456 30850529 10.1073/pnas.1900084116 PMC6442596

[koac132-B35] Jouannet V , Moreno AB, Elmayan T, Vaucheret H, Crespi MD, Maizel A (2012) Cytoplasmic Arabidopsis AGO7 accumulates in membrane-associated siRNA bodies and is required for ta-siRNA biogenesis. EMBO J 31: 1704–1713 22327216 10.1038/emboj.2012.20 PMC3321200

[koac132-B37] Kim EY , Wang L, Lei Z, Li H, Fan W, Cho J (2021) Ribosome stalling and SGS3 phase separation prime the epigenetic silencing of transposons. Nat Plants 7: 303–309 33649597 10.1038/s41477-021-00867-4

[koac132-B38] Krzyszton M , Kufel J (2022) Analysis of mRNA-derived siRNAs in mutants of mRNA maturation and surveillance pathways in Arabidopsis thaliana. Sci Rep 12: 1474 35087200 10.1038/s41598-022-05574-4 PMC8795450

[koac132-B39] Lanet E , Delannoy E, Sormani R, Floris M, Brodersen P, Crété P, Voinnet O, Robaglia C (2009) Biochemical evidence for translational repression by Arabidopsis microRNAs. Plant Cell 21: 1762–1768 19531599 10.1105/tpc.108.063412 PMC2714937

[koac132-B40] Langmead B , Salzberg SL (2012) Fast gapped-read alignment with Bowtie 2. Nat Methods 9: 357–359 22388286 10.1038/nmeth.1923 PMC3322381

[koac132-B41] Lejeune F , Li X, Maquat LE (2003) Nonsense-mediated mRNA decay in mammalian cells involves decapping, deadenylating, and exonucleolytic activities. Mol Cell 12: 675–687 14527413 10.1016/s1097-2765(03)00349-6

[koac132-B42] Li B , Ferreira MA, Huang M, Camargos LF, Yu X, Teixeira RM, Carpinetti PA, Mendes GC, Gouveia-Mageste BC, Liu C, et al (2019) The receptor-like kinase NIK1 targets FLS2/BAK1 immune complex and inversely modulates antiviral and antibacterial immunity. Nat Commun 10: 4996 31676803 10.1038/s41467-019-12847-6 PMC6825196

[koac132-B43] Li F , Wang A (2018) RNA decay is an antiviral defense in plants that is counteracted by viral RNA silencing suppressors. PLoS Pathog 14: e1007228 30075014 10.1371/journal.ppat.1007228 PMC6101400

[koac132-B44] Liao Y , Smyth GK, Shi W (2014) FeatureCounts: an efficient general purpose program for assigning sequence reads to genomic features. Bioinformatics 30: 923–930 24227677 10.1093/bioinformatics/btt656

[koac132-B45] Liu L , Chen X (2016) RNA quality control as a key to suppressing RNA silencing of endogenous genes in plants. Mol Plant 9: 826–836 27045817 10.1016/j.molp.2016.03.011 PMC5123867

[koac132-B46] Liu MJ , Wu SH, Wu JF, Lin WD, Wu YC, Tsai TY, Tsai HL, Wu SH (2013) Translational Landscape of Photomorphogenic Arabidopsis. Plant Cell 25: 3699 24179124 10.1105/tpc.113.114769 PMC3877810

[koac132-B47] Liu Y , Schiff M, Marathe R, Dinesh-Kumar SP (2002) Tobacco Rar1, EDS1 and NPR1/NIM1 like genes are required for N-mediated resistance to tobacco mosaic virus. Plant J 30: 415–429 12028572 10.1046/j.1365-313x.2002.01297.x

[koac132-B48] Livak KJ , Schmittgen TD (2001) Analysis of relative gene expression data using real-time quantitative PCR and the 2(-Delta Delta C(T)) Method. Methods 25: 402–408 11846609 10.1006/meth.2001.1262

[koac132-B49] Lukhovitskaya N , Ryabova LA (2019) Cauliflower mosaic virus transactivator protein (TAV) can suppress nonsense-mediated decay by targeting VARICOSE, a scaffold protein of the decapping complex. Sci Rep 9: 7042 31065034 10.1038/s41598-019-43414-0 PMC6504953

[koac132-B50] Ma X , Nicole MC, Meteignier LV, Hong N, Wang G, Moffett P (2015) Different roles for RNA silencing and RNA processing components in virus recovery and virus-induced gene silencing in plants. J Exp Bot 66: 919–932 25385769 10.1093/jxb/eru447

[koac132-B51] Ma X , Zhou Y, Moffett P (2019) Alterations in cellular RNA decapping dynamics affect tomato spotted wilt virus cap snatching and infection in Arabidopsis. New Phytol 224: 789–803 31292958 10.1111/nph.16049

[koac132-B52] Martelli G , Castellano MA (1971) Light and electron microscopy of the intracellular inclusions of cauliflower mosaic virus. J Gen Virol 13: 133–140 4108668 10.1099/0022-1317-13-1-133

[koac132-B53] Martin M (2011) Cutadapt removes adapter sequences from high-throughput sequencing reads. EMBnet J 17: 10–12.

[koac132-B54] Martinez de Alba AE , Moreno AB, Gabriel M, Mallory AC, Christ A, Bounon R, Balzergue S, Aubourg S, Gautheret D, Crespi MD, et al (2015) In plants, decapping prevents RDR6-dependent production of small interfering RNAs from endogenous mRNAs. Nucleic Acids Res 43: 2902–2913 25694514 10.1093/nar/gkv119 PMC4357720

[koac132-B55] Martinière A , Gargani D, Uzest M, Lautredou N, Blanc S, Drucker M (2009) A role for plant microtubules in the formation of transmission-specific inclusion bodies of Cauliflower mosaic virus. Plant J 58: 135–146 19077170 10.1111/j.1365-313X.2008.03768.x

[koac132-B56] Meteignier LV , Zhou J, Cohen M, Bhattacharjee S, Brosseau C, Chan MG, Robatzek S, Moffett P (2016) NB-LRR signaling induces translational repression of viral transcripts and the formation of RNA processing bodies through mechanisms differing from those activated by UV stress and RNAi. J Exp Bot 67: 2353–2366 26889008 10.1093/jxb/erw042

[koac132-B57] Morel JB , Godon C, Mourrain P, Béclin C, Boutet SP, Feuerbach F, Proux F, Vaucheret H (2002) Fertile hypomorphic ARGONAUTE (ago1) mutants impaired in post-transcriptional gene silencing and virus resistance. Plant Cell 14: 629–639 11910010 10.1105/tpc.010358 PMC150585

[koac132-B58] Moreno AB , Martinez de Alba AE, Bardou F, Crespi MD, Vaucheret H, Maizel A, Mallory AC (2013) Cytoplasmic and nuclear quality control and turnover of single-stranded RNA modulate post-transcriptional gene silencing in plants. Nucleic Acids Res 41: 4699–4708 23482394 10.1093/nar/gkt152 PMC3632135

[koac132-B59] Motomura K , Le QT, Hamada T, Kutsuna N, Mano S, Nishimura M, Watanabe Y (2015) Diffuse decapping enzyme DCP2 accumulates in DCP1 foci under heat stress in Arabidopsis thaliana. Plant Cell Physiol 56: 107–115 25339350 10.1093/pcp/pcu151

[koac132-B60] Motomura K , Le QT, Kumakura N, Fukaya T, Takeda A, Watanabe Y (2012) The role of decapping proteins in the miRNA accumulation in Arabidopsis thaliana. RNA Biol 9: 644–152 22614834 10.4161/rna.19877

[koac132-B61] Mustroph A , Juntawong P, Bailey-Serres J (2009) Isolation of plant polysomal mRNA by differential centrifugation and ribosome immunopurification methods. Methods Mol Biol 553: 109–126 19588103 10.1007/978-1-60327-563-7_6

[koac132-B62] Nagarajan VK , Kukulich PM, von Hagel B, Green PJ (2019) RNA degradomes reveal substrates and importance for dark and nitrogen stress responses of Arabidopsis XRN4. Nucleic Acids Res 47: 9216–9230 31428786 10.1093/nar/gkz712 PMC6755094

[koac132-B63] Nakagawa T , Suzuki T, Murata S, Nakamura S, Hino T, Maeo K, Tabata R, Kawai T, Tanaka K, Niwa Y, et al (2007) Improved gateway binary vectors: high-performance vectors for creation of fusion constructs in transgenic analysis of plants. Biosci Biotechnol Biochem 71: 2095–2100 17690442 10.1271/bbb.70216

[koac132-B64] Park HS , Browning KS, Hohn T, Ryabova LA (2004) Eucaryotic initiation factor 4B controls eIF3‐mediated ribosomal entry of viral reinitiation factor. EMBO J 23: 1381–1391 14988734 10.1038/sj.emboj.7600140 PMC381412

[koac132-B65] Park HS , Himmelbach A, Browning KS, Hohn T, Ryabova LA (2001) A plant viral “reinitiation” factor interacts with the host translational machinery. Cell 106: 723–733 11572778 10.1016/s0092-8674(01)00487-1

[koac132-B66] Peltz SW , Brown AH, Jacobson A (1993) mRNA destabilization triggered by premature translational termination depends on at least three cis-acting sequence elements and one trans-acting factor. Genes Dev 7: 1737–1754 8370523 10.1101/gad.7.9.1737

[koac132-B67] Perea-Resa C , Carrasco-Lopez C, Catala R, Tureckova V, Novak O, Zhang W, Sieburth L, Jimenez-Gomez JM, Salinas J (2016) The LSM1-7 complex differentially regulates Arabidopsis tolerance to abiotic stress conditions by promoting selective mRNA decapping. Plant Cell 28: 505–520 26764377 10.1105/tpc.15.00867 PMC4790874

[koac132-B68] Perea-Resa C , Hernandez-Verdeja T, Lopez-Cobollo R, del Mar Castellano M, Salinas J (2012) LSM proteins provide accurate splicing and decay of selected transcripts to ensure normal Arabidopsis development. Plant Cell 24: 4930–4947 23221597 10.1105/tpc.112.103697 PMC3556967

[koac132-B69] Pooggin MM , Ryabova LA (2018) Ribosome shunting, polycistronic translation, and evasion of antiviral defenses in plant pararetroviruses and beyond. Front Microbiol 9: 644 29692761 10.3389/fmicb.2018.00644 PMC5902531

[koac132-B71] Raxwal VK , Simpson CG, Gloggnitzer J, Entinze JC, Guo W, Zhang R, Brown JWS, Riha K (2020) Nonsense-mediated RNA decay factor UPF1 is critical for posttranscriptional and translational gene regulation in Arabidopsis. Plant Cell 32: 2725–2741 32665305 10.1105/tpc.20.00244 PMC7474300

[koac132-B72] Roehr JT , Dieterich C, Reinert K (2017) Flexbar 3.0 - SIMD and multicore parallelization. Bioinformatics 33: 2941–2942 28541403 10.1093/bioinformatics/btx330

[koac132-B73] Roux ME , Rasmussen MW, Palma K, Lolle S, Regue AM, Bethke G, Glazebrook J, Zhang W, Sieburth L, Larsen MR, et al (2015) The mRNA decay factor PAT1 functions in a pathway including MAP kinase 4 and immune receptor SUMM2. EMBO J 34: 593–608 25603932 10.15252/embj.201488645 PMC4365030

[koac132-B74] Schepetilnikov M , Kobayashi K, Geldreich A, Caranta C, Robaglia C, Keller M, Ryabova LA (2011) Viral factor TAV recruits TOR/S6K1 signalling to activate reinitiation after long ORF translation. EMBO J 30: 1343–1356 21343906 10.1038/emboj.2011.39 PMC3094109

[koac132-B75] Schneider CA , Rasband WS, Eliceiri KW (2012) NIH Image to ImageJ: 25 years of image analysis. Nat Methods 9: 671–675 22930834 10.1038/nmeth.2089 PMC5554542

[koac132-B76] Schoelz JE , Goldberg KB, Kiernan J (1991) Expression of cauliflower mosaic virus (CaMV) gene VI in transgenic Nicotiana bigelovii complements a strain of CaMV defective in long-distance movement in nontransformed N. bigelovii. Mol Plant-Microbe Interact 4: 350–355

[koac132-B77] Schoelz JE , Leisner S (2017) Setting up shop: the formation and function of the viral factories of cauliflower mosaic virus. Front Plant Sci 8: 1832 29163571 10.3389/fpls.2017.01832 PMC5670102

[koac132-B78] Shivaprasad PV , Rajeswaran R, Blevins T, Schoelz J, Meins F Jr, Hohn T, Pooggin MM (2008) The CaMV transactivator/viroplasmin interferes with RDR6-dependent trans-acting and secondary siRNA pathways in Arabidopsis. Nucleic Acids Res 36: 5896–5909 18801846 10.1093/nar/gkn590 PMC2566869

[koac132-B79] Sorenson RS , Deshotel MJ, Johnson K, Adler FR, Sieburth LE (2018) Arabidopsis mRNA decay landscape arises from specialized RNA decay substrates, decapping-mediated feedback, and redundancy. Proc Natl Acad Sci USA 115: E1485–E1494 29386391 10.1073/pnas.1712312115 PMC5816150

[koac132-B80] Souret FF , Kastenmayer JP, Green PJ (2004) AtXRN4 degrades mRNA in Arabidopsis and its substrates include selected miRNA targets. Mol Cell 15: 173–183 15260969 10.1016/j.molcel.2004.06.006

[koac132-B81] Tani H , Mizutani R, Salam KA, Tano K, Ijiri K, Wakamatsu A, Isogai T, Suzuki Y, Akimitsu N (2012) Genome-wide determination of RNA stability reveals hundreds of short-lived noncoding transcripts in mammals. Genome Res 22: 947–956 22369889 10.1101/gr.130559.111 PMC3337439

[koac132-B82] Tebaldi T , Re A, Viero G, Pegoretti I, Passerini A, Blanzieri E, Quattrone A (2012) Widespread uncoupling between transcriptome and translatome variations after a stimulus in mammalian cells. BMC Genom 13: 220 10.1186/1471-2164-13-220 PMC3441405 22672192

[koac132-B83] Teixeira D , Sheth U, Valencia-Sanchez MA, Brengues M, Parker R (2005) Processing bodies require RNA for assembly and contain nontranslating mRNAs. RNA (New York, NY) 11: 371–382 10.1261/rna.7258505 PMC1370727 15703442

[koac132-B84] Thran M , Link K, Sonnewald U (2012) The Arabidopsis DCP2 gene is required for proper mRNA turnover and prevents transgene silencing in Arabidopsis. Plant J 72: 368–377 22639932 10.1111/j.1365-313X.2012.05066.x

[koac132-B85] Walsh D , Mathews MB, Mohr I (2013) Tinkering with translation: protein synthesis in virus-infected cells. Cold Spring Harbor Perspect Biol 5: a012351–a012351 10.1101/cshperspect.a012351 PMC3579402 23209131

[koac132-B86] Wang C , Schmich F, Srivatsa S, Weidner J, Beerenwinkel N, Spang A (2018) Context-dependent deposition and regulation of mRNAs in P-bodies. eLife 7: e29815 29297464 10.7554/eLife.29815 PMC5752201

[koac132-B87] Wu H , Li B, Iwakawa HO, Pan Y, Tang X, Ling-hu Q, Liu Y, Sheng S, Feng L, Zhang H, et al (2020) Plant 22-nt siRNAs mediate translational repression and stress adaptation. Nature 581: 89–93 32376953 10.1038/s41586-020-2231-y

[koac132-B88] Xie Z , Johansen LK, Gustafson AM, Kasschau KD, Lellis AD, Zilberman D, Jacobsen SE, Carrington JC (2004) Genetic and functional diversification of small RNA pathways in plants. PLoS Biol 2: e104 15024409 10.1371/journal.pbio.0020104 PMC350667

[koac132-B89] Xing W , Muhlrad D, Parker R, Rosen MK (2020) A quantitative inventory of yeast P body proteins reveals principles of composition and specificity. Elife 9: e56525 32553117 10.7554/eLife.56525 PMC7373430

[koac132-B90] Xu G , Greene GH, Yoo H, Liu L, Marqués J, Motley J, Dong X (2017) Global translational reprogramming is a fundamental layer of immune regulation in plants. Nature 545: 487–490 28514447 10.1038/nature22371 PMC5485861

[koac132-B91] Xu J , Chua NH (2009) Arabidopsis decapping 5 is required for mRNA decapping, P-body formation, and translational repression during postembryonic development. Plant Cell 21: 3270–3279 19855049 10.1105/tpc.109.070078 PMC2782270

[koac132-B92] Xu J , Chua NH (2012) Dehydration stress activates Arabidopsis MPK6 to signal DCP1 phosphorylation. EMBO J 31: 1975–1984 22407295 10.1038/emboj.2012.56 PMC3343339

[koac132-B93] Xu J , Yang JY, Niu QW, Chua NH (2006) Arabidopsis DCP2, DCP1, and VARICOSE form a decapping complex required for postembryonic development. Plant Cell 18: 3386–3128 3147 17158604 10.1105/tpc.106.047605 PMC1785416

[koac132-B94] Ye J , Yang J, Sun Y, Zhao P, Gao S, Jung C, Qu J, Fang R, Chua NH (2015) Geminivirus activates ASYMMETRIC LEAVES 2 to accelerate cytoplasmic DCP2-mediated mRNA turnover and weakens RNA silencing in Arabidopsis. PLoS Pathog 11: e1005196 26431425 10.1371/journal.ppat.1005196 PMC4592220

[koac132-B95] Yu X , Li B, Jang GJ, Jiang S, Jiang D, Jang JC, Wu SH, Shan L, He P (2019) Orchestration of processing body dynamics and mRNA decay in Arabidopsis immunity. Cell Rep 28: 2194–2205 e6 31433992 10.1016/j.celrep.2019.07.054 PMC6716526

[koac132-B96] Zhang ZJ , Gao Q, Fang XD, Ding ZH, Gao DM, Xu WY, Cao Q, Qiao JH, Yang YZ, Han C, et al (2020) CCR4, a RNA decay factor, is hijacked by a plant cytorhabdovirus phosphoprotein to facilitate virus replication. Elife 9: e53753 32207684 10.7554/eLife.53753 PMC7105381

[koac132-B97] Zid BM , O'Shea EK (2014) Promoter sequences direct cytoplasmic localization and translation of mRNAs during starvation in yeast. Nature 514: 117–121 25119046 10.1038/nature13578 PMC4184922

[koac132-B98] Zorzatto C , Machado JP, Lopes KV, Nascimento KJ, Pereira WA, Brustolini OJ, Reis PA, Calil IP, Deguchi M, Sachetto-Martins G, et al (2015) NIK1-mediated translation suppression functions as a plant antiviral immunity mechanism. Nature 520: 679–682 25707794 10.1038/nature14171 PMC4779052

[koac132-B99] Zuo Z , Roux M, Rodriguez E, Petersen M (2022) mRNA decapping factors LSM1 and PAT paralogs are involved in turnip mosaic virus viral infection. Mol Plant Microbe Interact 5: 125–130 10.1094/MPMI-09-21-0220-SC 35100808

