# Supplementary material for: Arabidopsis RNA processing body components LSM1 and DCP5 aid in the evasion of translational repression during Cauliflower mosaic virus infection
Source: Plant Cell. 2022 May 2;34(8):3128–47. doi: 10.1093/plcell/koac132 (PMC9338796; doi:10.1093/plcell/koac132)
Supplement: koac132_Supplementary_Data [file koac132_supplementary_data.zip › koac132-suppl_data/tpc.22.00174_SupplementalFigures.pdf]

Supplemental Data. Hoffmann et al. (2022). Arabidopsis RNA processing body components LSM1 and DCP5 aid in the evasion of translational repression during *Cauliflower mosaic virus* infection. Plant Cell.

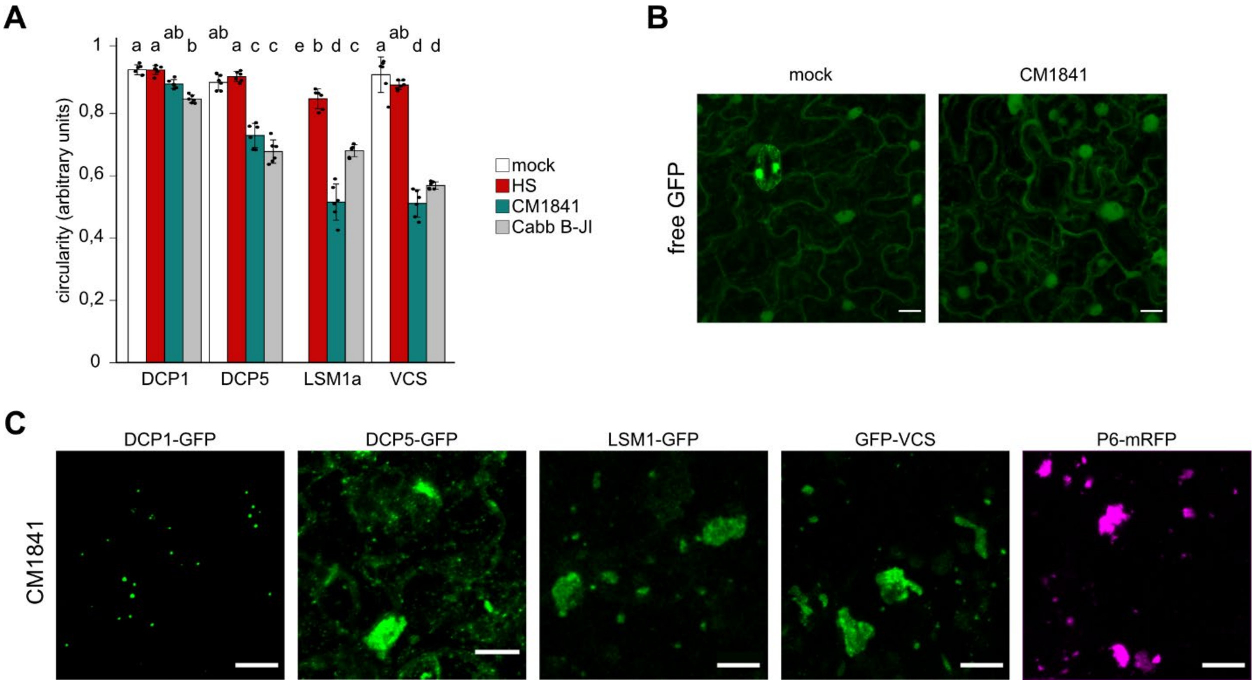

Supplemental Figure S1. Description of PB markers during CaMV infection (Supports Figure 1.)
(A) Average circularity of detected foci under each condition at 21 dpi determined by ImageJ circularity masking. Error bars represent standard deviation. Significant differences were determined by one-way ANOVA coupled with Tukey's HSD test (a= 0.05), letters indicate statistical groups. Values calculated from six independent replicates. **(B)** Confocal images composed of confocal Z-stacks of free GFP under mock conditions and 21 dpi CM1841 infection (Scale bars = 10 µm). **(C)** Confocal images composed of confocal Z-stacks of PB markers and CaMV P6 five to seven weeks after infection, imaged genotypes are indicated above the micrographs (Scale bars = 10 µm).

Supplemental Data. Hoffmann et al. (2022). Arabidopsis RNA processing body components LSM1 and DCP5 aid in the evasion of translational repression during *Cauliflower mosaic virus* infection. Plant Cell.

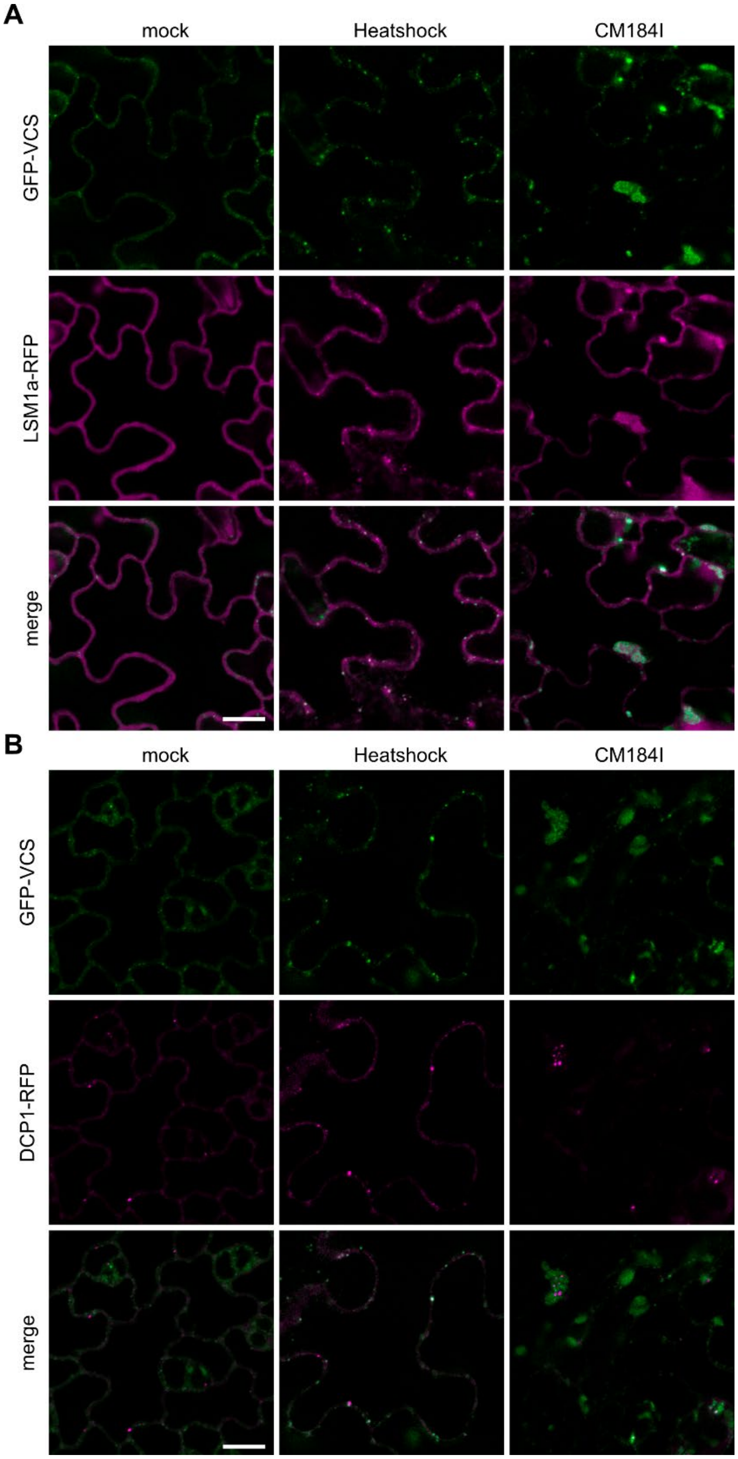

Supplemental Figure S2. PB double marker lines show co-assembly after heat shock and during CaMV infection (Supports Figure 1. and 2.)
Co-localization of GFP-VCS with LSM1-RFP (upper panel) and DCP1-RFP (lower panel) in transgenic Arabidopsis 21 days after mock, heat shock, or CaMV infection. Images represent single plane micrographs (Scale bars = 10 µm).

Supplemental Data. Hoffmann et al. (2022). Arabidopsis RNA processing body components LSM1 and DCP5 aid in the evasion of translational repression during *Cauliflower mosaic virus* infection. Plant Cell.

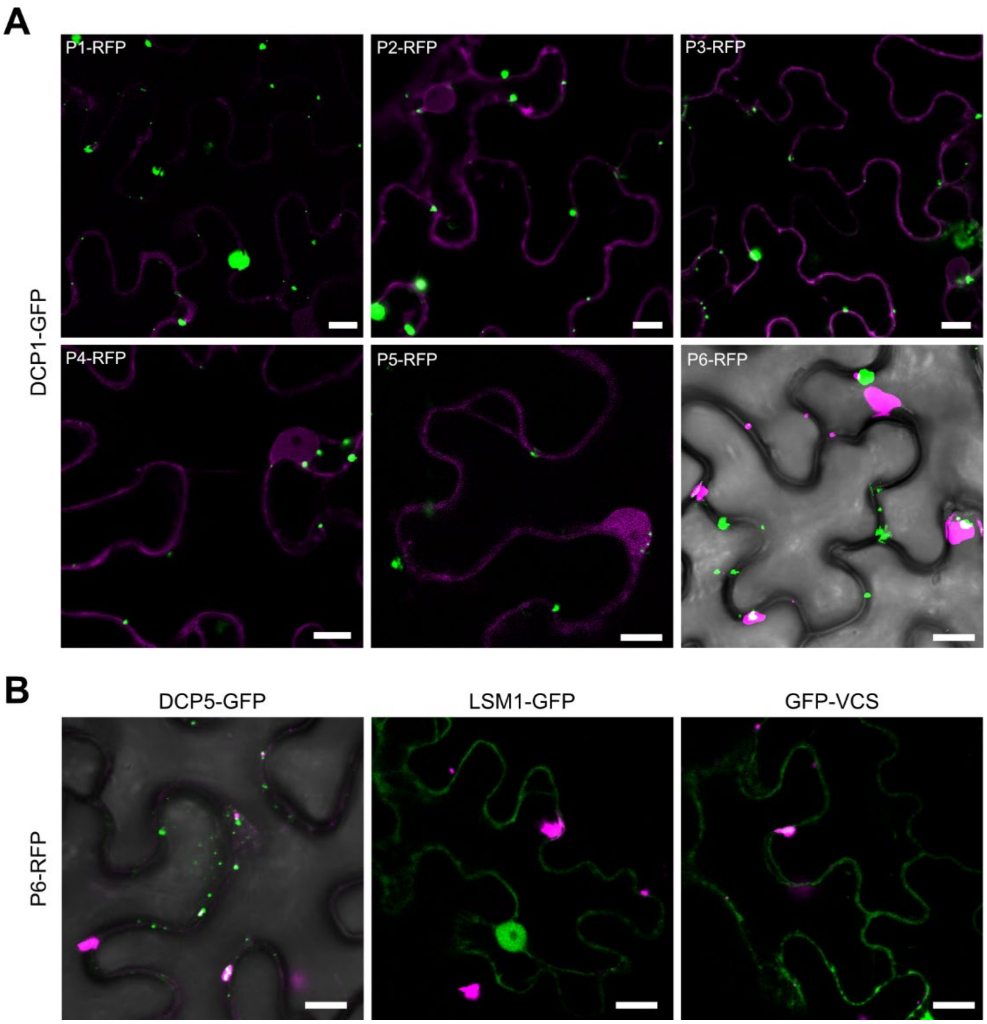

Supplemental Figure S3. Co-expression of PB components with CaMV proteins in *Nicotiana benthamiana* (Supports Figure 2.)
(A) Viral proteins fused to tagRFP were co-expressed with AtDCP1-GFP in *Nicotiana benthamiana* leaves. Single plane images were taken two days after co-infiltration. Brightfield channel is included when neither of the expressed proteins are soluble enough to outline the cell contour. (Scale bars = 10 µm). **(B)** PB components were co-expressed with BJI P6-tag-mRFP. Single plane images were taken two days after co-infiltration in *Nicotiana benthamiana* leaves. (Scale bars = 10 µm). Brightfield channel as in (A).

Supplemental Data. Hoffmann et al. (2022). Arabidopsis RNA processing body components LSM1 and DCP5 aid in the evasion of translational repression during *Cauliflower mosaic virus* infection. Plant Cell.

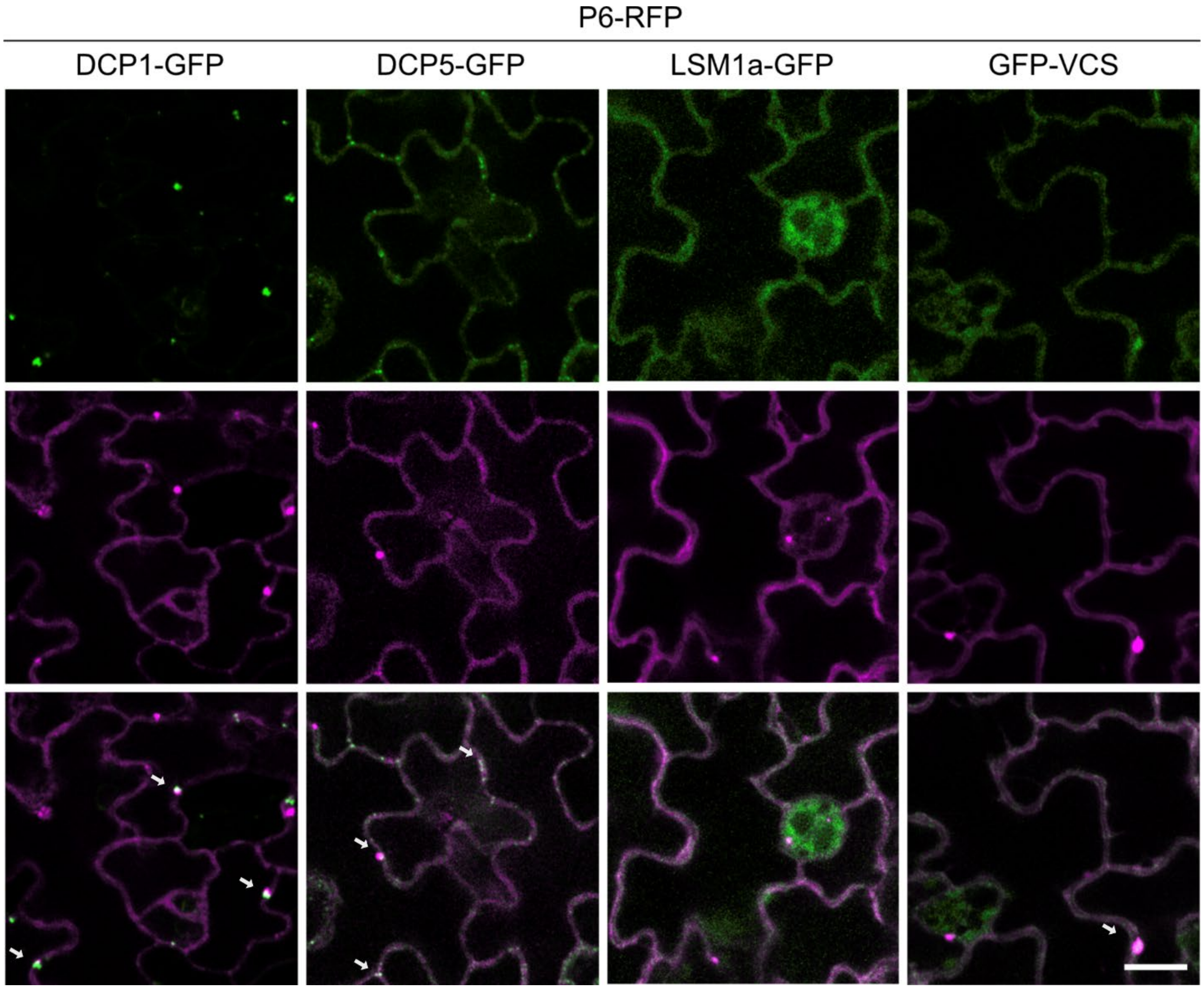

Supplemental Figure S4. Co-localization of P6 with PB components under mock conditions (Supports Figure 2.)
Co-localization of P6-mRFP with PB markers in transgenic Arabidopsis 21 days after mock infection. White arrows point to co-localizations. Representative single plane images are shown (Scale bars = 10 µm). The experiments were replicated at least three times with independent transformants.

Supplemental Data. Hoffmann et al. (2022). Arabidopsis RNA processing body components LSM1 and DCP5 aid in the evasion of translational repression during *Cauliflower mosaic virus* infection. Plant Cell.

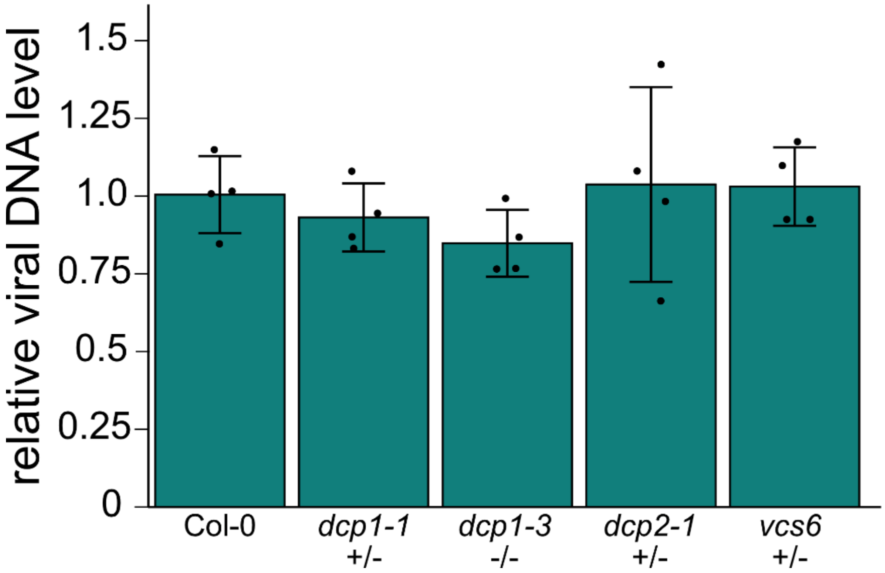

Supplemental Figure S5. Viral DNA accumulation in additional PB mutants (Supports Figure 3.)
Viral DNA accumulation in systemic leaves at 21 dpi in the indicated genotypes, determined by qRT-PCR. Values represent means ± SD (n = 4) relative to Col-0 plants and normalized to *18S* ribosomal DNA as the internal reference. Individual plants were genotyped before harvest in heterozygous lines.

Supplemental Data. Hoffmann et al. (2022). Arabidopsis RNA processing body components LSM1 and DCP5 aid in the evasion of translational repression during *Cauliflower mosaic virus* infection. Plant Cell.

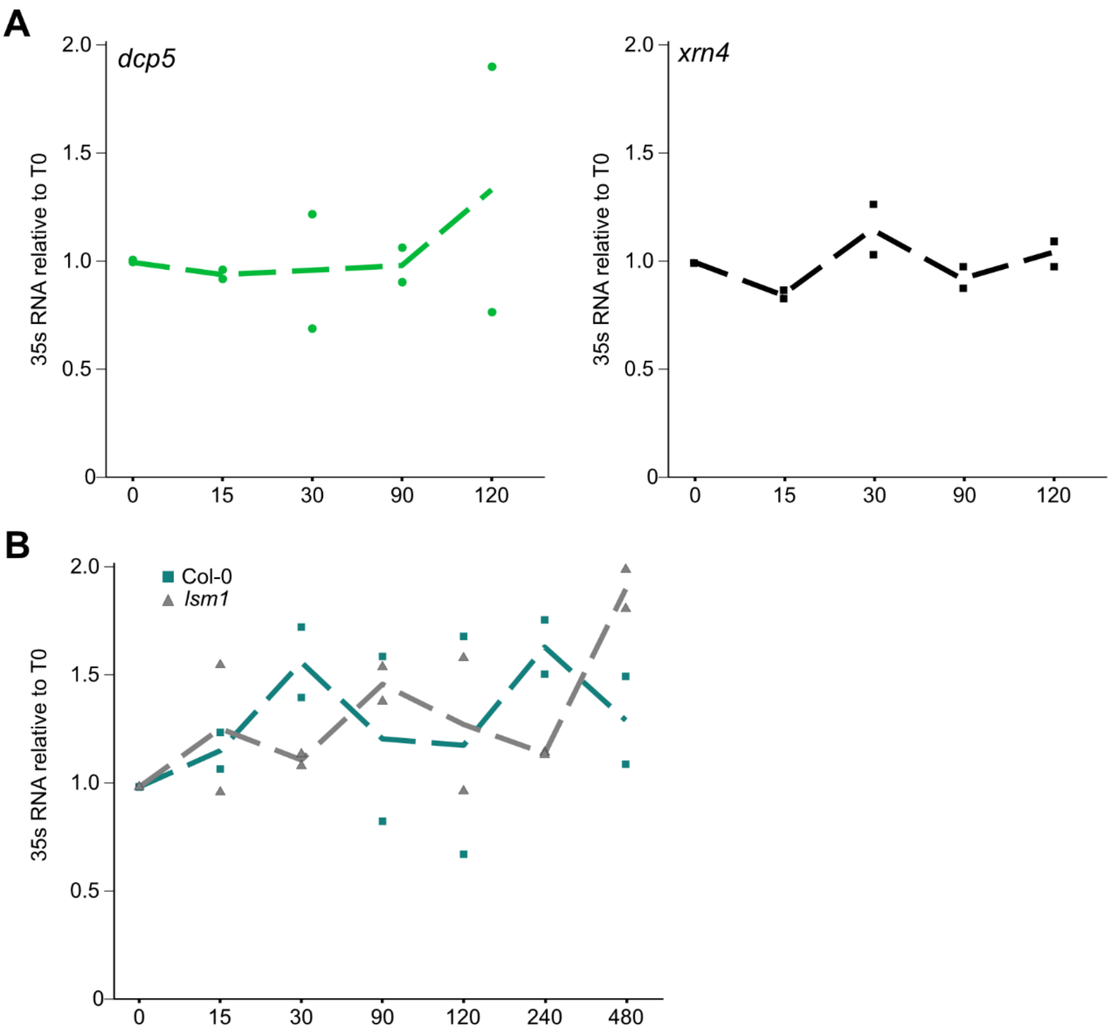

Supplemental Figure S6. *35s* RNA decay after transcriptional arrest with cordycepin (Supports Figure 4.)
(A) Dotted line represents the average of two biological replicates, single experiments are shown by circles (*dcp5*) and squares (*xrn4*). Sampling timepoints (min after treatment) are indicated on the x-axis.**(B)** Long-term transcript decay profiles for viral *35s* RNA. Dotted line represents the average of two biological replicates, single experiments are shown by circles (Col-0) and triangles (*lsm1*). Sampling timepoints (min after treatment) are indicated on the x-axis.

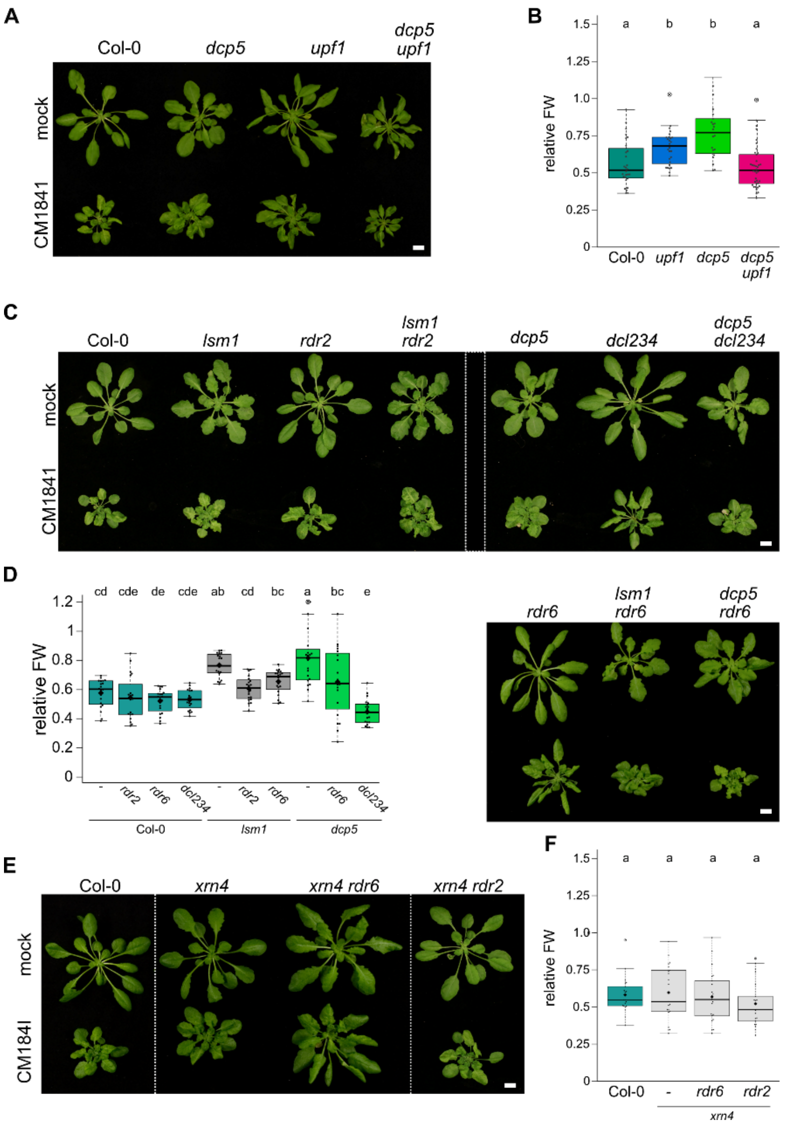

Supplemental Figure S7. CaMV disease in combinatorial mutants with NMD and RNA silencing (Supports Figure 5.)

(A) Infection phenotypes of the indicated genotypes at 21 dpi with CM1841 (lower panel) and mock (upper panel) (scale bar = 1 cm). **(B)** Relative fresh weight of infected/control plants (n>20) corresponding to (A). The box represents the interquartile range (IQR), the solid lines represent the median, diamonds the average. The whiskers extend to a maximum of 1.5 × IQR beyond the box.Letters indicate statistical groups determined by one-way ANOVA followed by Tukey's HSD test (a= 0.05) **(C)** Infection phenotypes of the indicated genotypes at 21 dpi with CM1841 (lower panel) and mock (upper panel) (scale bar = 1 cm). **(D)** Relative fresh weight of infected/control plants (n=20) corresponding to (C). The box represents the interquartile range (IQR), the solid lines represent the median, diamonds the average. The whiskers extend to a maximum of 1.5 × IQR beyond the box. Letters indicate statistical groups determined by one-way ANOVA followed by Tukey's HSD test (a= 0.05) **(E)** Infection phenotypes of the indicated genotypes at 21 dpi with CM1841 (lower panel) and mock (upper panel) (scale bar = 1 cm). **(F)** Relative fresh weight of infected/control plants (n=20) corresponding to (E). The box represents the interquartile range (IQR), the solid lines represent the median, diamonds the average. The whiskers extend to a maximum of 1.5 × IQR beyond the box. Letters indicate statistical groups determined by one-way ANOVA followed by Tukey's HSD test (a= 0.05).

Supplemental Data. Hoffmann et al. (2022). Arabidopsis RNA processing body components LSM1 and DCP5 aid in the evasion of translational repression during *Cauliflower mosaic virus* infection. Plant Cell.

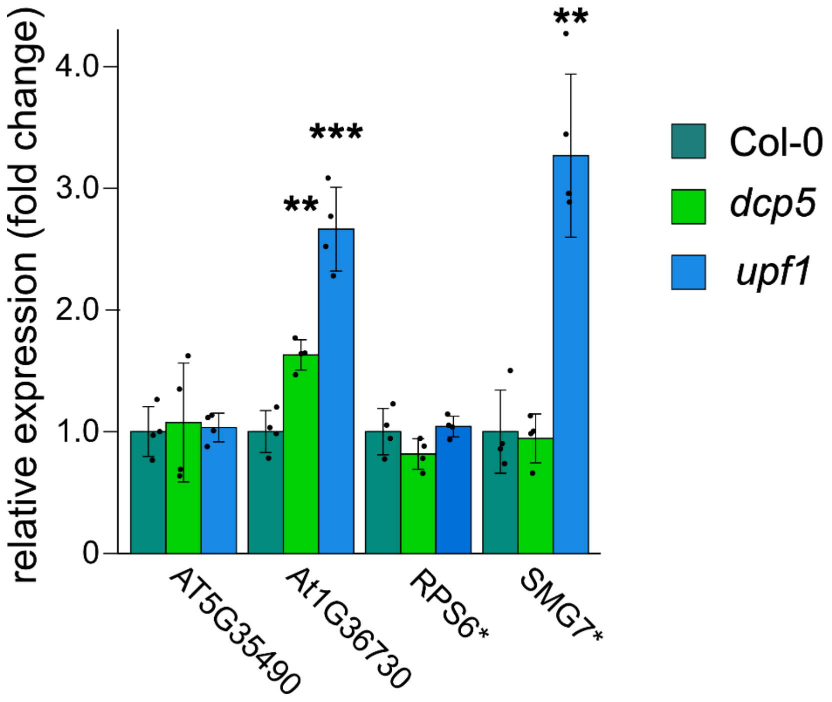

Supplemental Figure S8. Expression of NMD targets in *dcp5* and *upf1* during CaMV infection (Supports Figure 5.)
Relative expression of NMD targets in CM1841-infected Col-0, *dcp5* and *upf1* plants determined by qRT-PCR. Values represent means ± SD (n = 4) relative to infected Col-0 plants and normalized to *PP2a* as the internal reference. Statistical significance was calculated by two-sided Student's t-tests (**PÒ0.01; ***PÒ0.001). Inf
replicated at least three times independently. Supports Figure 5.

Supplemental Data. Hoffmann et al. (2022). Arabidopsis RNA processing body components LSM1 and DCP5 aid in the evasion of translational repression during *Cauliflower mosaic virus* infection. Plant Cell.

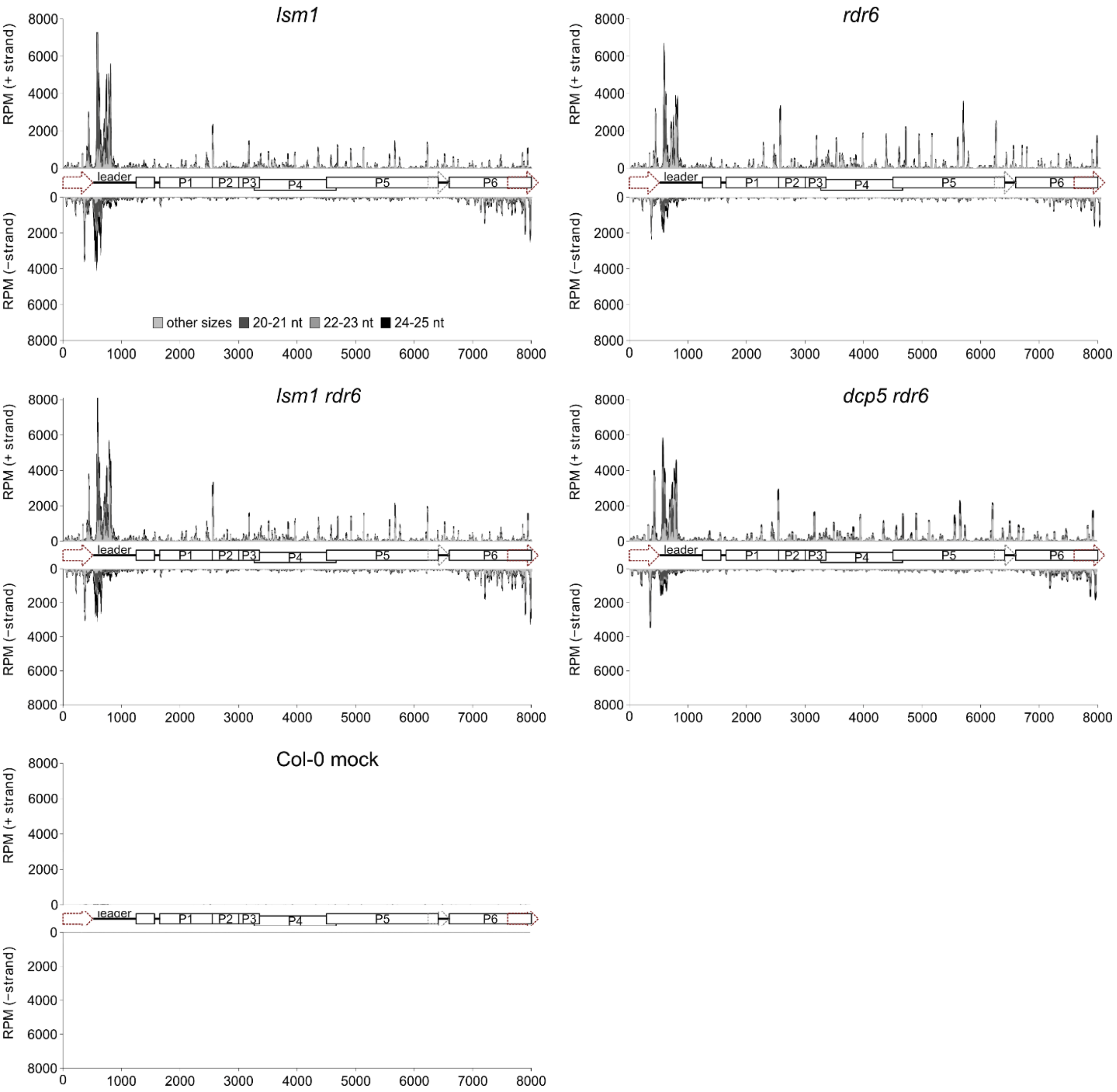

Supplemental Figure S9. sRNA profiles in *lsm1* and combinatorial mutants (Supports Figure 6.)
Coverage plots of 18-26 nt sRNA profiles along the 8031 bp viral genome in the indicated genotypes at 21 dpi with CMI184I. The starting position was set to the beginning of the *35s* promoter (genomic position 7090 bp). Genomic features are annotated as depicted in Figure 5C.

Supplemental Data. Hoffmann et al. (2022). Arabidopsis RNA processing body components LSM1 and DCP5 aid in the evasion of translational repression during *Cauliflower mosaic virus* infection. Plant Cell.

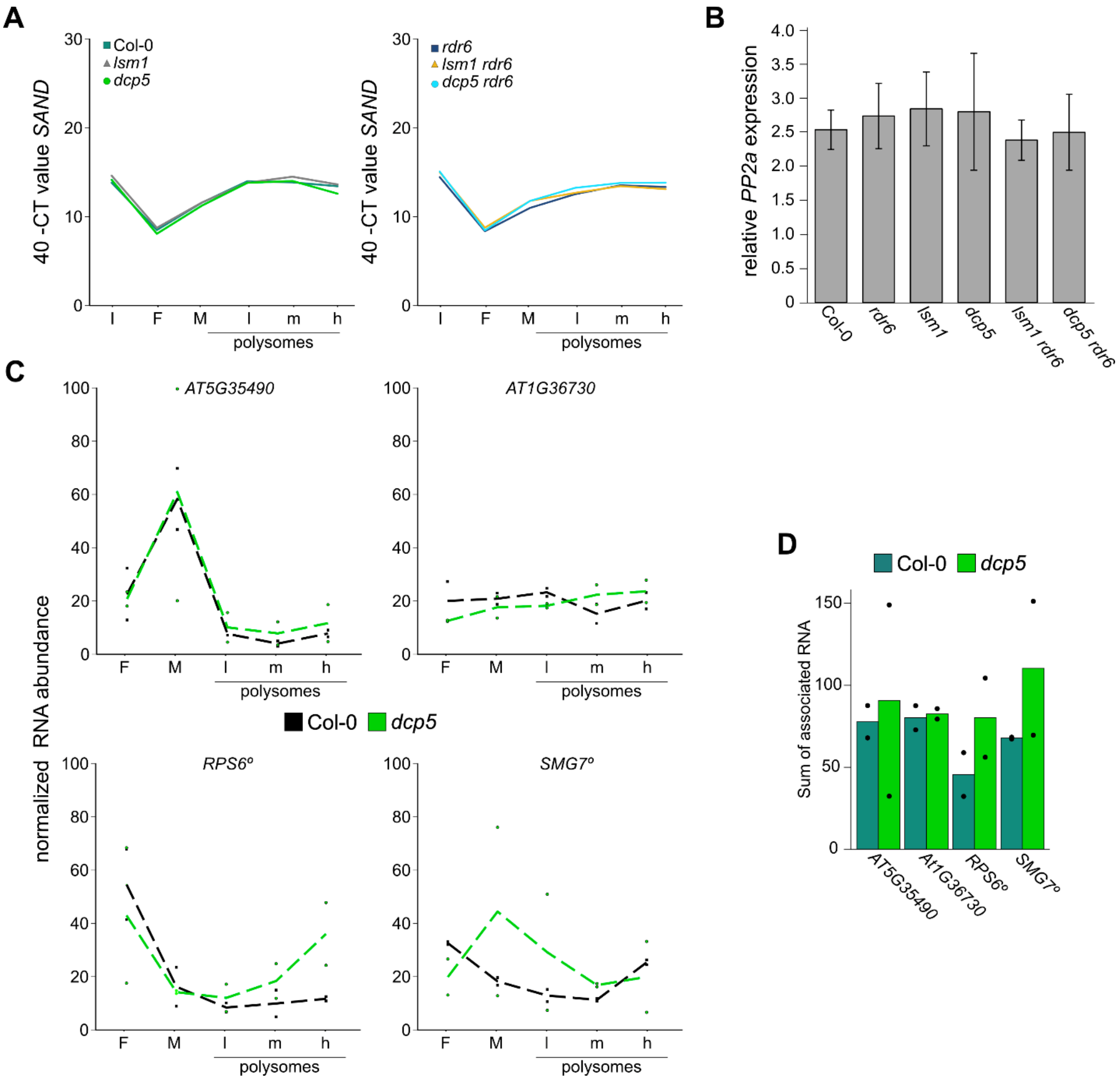

Supplemental Figure 10: Translational profiling during CaMV infection (Supports Figure 7.)

(A) Distribution of *SAND* (40-CT value) in collected fractions. Solid lines represent the average of three replicates in the indicated genotypes. Fractions represent input (I), free (F), monosome (M), light (l), medium (m), and heavy (h) polysome-associated RNA. **(B**) Relative expression of *PP2a* in input samples. Values represent means ± SD (n = 3) normalized to *SAND.* **(C)** RNA abundance of the indicated transcripts in Col-0 and *dcp5*. The experiment was performed two times using material from independent infections. Fractionated RNA was normalized to *SAND* and depicted as fractions of total RNA abundance. Dashed lines represent the average of biological replicates, dots represent single experiments of the indicated genotypes. Measured fractions represent free (F), monosome (M), light (l), medium (m) and heavy (h) polysome-associated RNA. **(D)** Sum of ribosome-associated RNA of the indicated transcripts in Col-0 and *dcp5* from (C). Dots represent single experiments.
