# Supplementary material for: Arabidopsis RNA processing body components LSM1 and DCP5 aid in the evasion of translational repression during Cauliflower mosaic virus infection
Source: Plant Cell. 2022 May 2;34(8):3128–47. doi: 10.1093/plcell/koac132 (PMC9338796; doi:10.1093/plcell/koac132)
Supplement: koac132_Supplementary_Data [file tpc22.00174.PeerReview.pdf › koac132-suppl_data/tpc22.00174.PeerReview.pdf]

Arabidopsis RNA processing body components LSM1 and DCP5 aid in the evasion of translational repression during *Cauliflower mosaic virus* infection

Gesa Hoffmann, Amir Mahboubi, Heinrich Bente, Damien Garcia, Johannes Hanson, and Anders Hafrén

Corresponding author: Anders Hafrén anders.hafren@slu.se.

Review timeline:

**TPC2021-RA-00485**	Submission received:	June 9, 2021
	1st Decision:	July 21, 2021 *manuscript declined*
**TPC2022-RA-00174**	Submission received:	Feb. 15, 2022
	1st Decision:	March 26, 2022 *revision requested*
**TPC2022-RA-00174R1**	1st Revision received:	April 8, 2022
	2nd Decision:	April 14, 2022 *acceptance pending, sent to science editor*
	Final acceptance:	April 24, 2022

REPORT: (The report shows the major requests for revision and author responses. Minor comments for revision and miscellaneous correspondence are not included. The original format may not be reflected in this compilation, but the reviewer comments and author responses are not edited, except to correct minor typographical or spelling errors that could be a source of ambiguity.)

TPC2021-RA-00485 1st Editorial decision – *declined* July 21, 2021

As you will see in the reviewers comments, all three reviewers had positive comments regarding the potential of the results presented. However, they also agreed that additional experiments were required to support the conclusions made in the manuscript. In particular, it was suggested that some of the biochemical analyses should be repeated in certain mutant backgrounds (most of which are already described in the manuscript) in order to validate the predictions resulting from the results presented.

-- Reviewer comments:

Reviewer #1 (Comments for the Author):
The manuscript by Hoffmann et al described an interesting role of processing body components, especially DCP5 and LSM1, in facilitating the association of 35S viral RNAs with viral factories (VFs). By assisting the association of viral RNA with VFs upon viral infection, these p-body components help to triage the viral RNAs preferably to translation by reducing their susceptibility of RDR6-mediated translation repression. The authors made these claims first by the observations of PB component proteins with the irregular-sized/shaped VFs. The comprehensive survey of viral RNA, DNA and proteins in combinatorial mutants defective in PB, NMD and VIGS pathways led the authors to conclude that the viral RNAs are not subjected to RNA decay via PB's decapping functions, nor NMD. Rather, PB proteins are essential to direct viral RNAs to VFs where they can escape from RDR6-mediated translation repression and more prone to be translated, as a way to promote viral replication and propagation. The finding is innovative to some extent. Before it's considered for publication, a few specific points should be addressed.

1. The title "Cauliflower Mosaic Virus Utilizes Processing Bodies to Escape Translational Repression in Arabidopsis" is somewhat misleading. In this study, PBs are not the site for sequestering the viral RNAs, nor for active translation.
2. The authors claimed that RDR6-mediated translation repression can act on viral RNAs (Figure 8 and in Discussion). This statement was probably made by prior observation that 35S viral RNA is resistant to gene silencing and no viral RNA changes were seen in this study (Figure 3, S6). It is confusion to the readers that, instead of checking the translation repression roles, the authors were investigating the contribution of RDR6-mediated gene silencing in Figure 6. Whether RDR6 really renders the translation capacity of 35S viral RNAs could at least be tested/compared via the polysome association in mutants defective in both PB component and in RDR6/DCLs. Further increased polysome association will likely be seen in dcp5 rdr6 mutant, compared to the single mutants? And, to rule out the RDR6-mediated gene silencing, there should be no increased 35S-derived siRNAs in PB

mutants?
3. The biological significance of the PB component facilitated viral RNA association with VFs could be strengthened by the reduced association of viral RNAs with VF in PB mutants?
4. Some data presentation will benefit from the perspective of quantification. For example, in Figure 5 the differential association of 35S RNA with polysomes in wild type than in PB mutants was used to infer a higher translation rate of viral RNA in the presence of PB components. Instead of profiling the fractions 1-12, which cover monosome to high-order polysome fractions, the free viral RNAs or those under small subunit scanning were not included (except the total amount was measured in Figure 5C). It may be helpful to compare the viral RNA association with different sucrose gradient fractions for free, subunits/monosomes and polysomes in different genetic backgrounds to show the differential distributions. Spike-in RNAs should be used to ensure the differences were not a results of RNA extraction efficiencies among fractions. Similarly, in Figure 6, RSP6 and SMG7 have increased association with mono-polyribosomes. Is this due to enhanced translation in dcp5 or simply because their mRNAs levels are increased in dcp5? Also, Figure 7D needs biological replicates or quantitative data to reinforce the conclusion.
5. Lines 163-166. One cannot rule out translational or post-translational (increased protein stability) regulation of PB mRNAs upon virus infections.
6. Lines 174-176. Data in Supplemental Figures 3 and 4 do not support PB association with VFs.
Minor points
1. Is it RPS6 or RSP6?
2. Some of Figures label lsm1 +, dcp5 +... This can be confused as heterozygous.
3. Line 159, DCP1 is not always exclusive from the virus-induced clusters.

Reviewer #2 (Comments for the Author):
RNA silencing is the most important antiviral system in plants. Therefore, for efficient infection, viruses should suppress or evade silencing-mediated defense. Indeed, plant viruses have evolved different strategies to overcome or evade antiviral silencing. The DNA virus CaMV expresses P6 protein, which inhibits silencing in the nucleus presumably by binding DRB4 DICER co-factor. Moreover, it inhibits (exhausts) the silencing effector complexes as the 35S RNS leader of CaMV forms a strong hairpin, from which large amount of silencing inefficient siRNAs are generated.

In this manuscript (entitled "Cauliflower Mosaic Virus Utilizes Processing Bodies to Escape Translational Repression in Arabidopsis"), the authors provide evidence that CaMV also utilizes certain host encoded P-body (PB) components to evade the translational repression branch of silencing mediated defense.

They have shown that in CaMV-infected Arabidopsis, the LSM, VCS, DCP5 PB components (but not the DCP1 PB factor) relocalize into the viral factories (VF), which are essential for infection and are mainly composed of P6 viral protein and viral RNAs. They also demonstrate that in the absence of LSM1 or DCP5 proteins the CaMV replication/infection is less efficient, the viral DNA and protein levels are massively reduced, while the viral RNA accumulation is only slightly modified. To explain it, they provide evidence that in the lsm1 and dcp5 mutants viral RNAs are less efficiently translated. Finally, they show that lsm1/rdr6, dcp5/rdr6 and dcp5/dcr234 complex mutants the CaMV replication/infection is restored, the viral DNA and protein levels are normal. Thus they concluded that during infection, the CaMV recruits certain PB components of the host, which enhances viral translation/replication and help the virus to circumvent translational repression branch of silencing defense.

It is an interesting study that significantly contributes to our understanding about the molecular details of host-virus interactions, showing a novel mechanism how viruses can utilize host factors to evade silencing. On the other hand, the study should be improved, it suffers from relatively easily correctable weaknesses.

Major points
1, The study is not complete. The final and most important result is that, in the absence of P-body components silencing machinery represses translation of viral RNA. Unfortunately it is not analyzed in proper details. To prove that normal translation is restored in the double mutants, polysome profile assays should be also conducted using the PB-silencing double mutant plants (lsm1/rdr6, dcp5/rdr6, dcp5/dcr234). As the lines are there and the methodology is in hand, it can be conducted easily.

I think that comparative small RNA seq assays should also be conducted to unravel whether silencing based translational repression is associated with accumulation of specific viral sRNAs or not.
2, The manuscript is not carefully written, the authors did not use the definitions coherently, which is misleading at many points. They should very carefully re-read their own manuscripts and use the terminology coherently. For instance:
- The title is misleading, overstating. It says that CaMV "utilizes processing bodies to escape translational repression". I think it is not correct, they found that certain P-body components are relocalized into VFs (while DCP1 not) and that two PB factors stimulates viral translation/replication. But they did not show evidence that PB is directly used for evasion of translational repression. They should say instead that CaMV utilizes certain processing body components to escape translational repression. Actually at the end of abstract they wrote more correctly: "PB components are co-opted by the virus to maintain efficient translation". Please correct it in the title and throughout the manuscript.
- DCP1 is not recruited to the VF and its role in CaMV infection was not studied. Despite in their model (FIG8) they show it as part of PB that is recruited to the viral RNA and P6. It is a very speculative element (which is actually not supported by the results). So the figure should be modified or they should note that it is highly a speculative element.
- The text is confusing at certain points. For instance, in the abstract they write viral replication factories, later they write Viral Factories (VF) and emphasize that "These VFs are structurally different from the membranous RNA virus replication complexes" (Page 13). It is confusing.
- Additional little inaccuracies in the abstract: “we could establish PB components as essential helpers in viral RNA translation" (These factors were not essential, they stimulate translation but even in their absence infection was efficient).
- The introduction section should be modified make it easier for non-virologist to understand. At least the CaMV, P6 and VF should be shortly introduced.
3, Most of the conclusions are nicely supported by the results. However, in certain figures I see some discrepancies.
-Page 6, Fig 1E: "The transcript levels were elevated for DCP1 and more strongly for VCS with both CaMV strains but not for the other components (Figure 1E)," DCP2 was also significantly enhanced according to the figure 1E.
-P.8, Fig3: I think that description, Fig3E and F slightly different. "viral inclusion protein P6, the coat protein P4 and the virion-associated protein P3 accumulated less in both lsm1 and dcp5 but not xrn4 (Figure 3E)."I think Fig 3E shows that P6 and especially P4 were also reduced in xrn4 mutant (left panel). "Both viral strains were similarly impaired in DNA and protein accumulation," Fig3E supports the conclusion, while 3F show differently. At Fig3F CM1841 P3 and P6 levels are significantly reduced in both lsm1 and dcp5 mutants, whereas at Cabb infection only the P3 in lsm1 mutant is significantly reduced.

Minor points
5, I propose to change the order of the three sections in the Discussion, the NMD part should be the last one. It interrupts the flow, the main story is the PB factor, translation repression, silencing and the NMD is only a side results.
6, NMD is only superficially (and not very correctly) described. NMD does not generally measure integrity of RNA and identify in general aberrant RNAs, NMD has characteristic targets. It identifies an mRNA as an aberrant one if its translation termination is inefficient because the 3' UTR is very long or contains EJCs. SMG7 does not contain a PTC, it is an NMD factor, whose normal mRNA contain NMD inducing features (its 3'UTR is long and harbors introns). Thus it is targeted by NMD.
7, In the lsm1 and dcp5 mutants, viral translation is impaired. It would be informative to test whether the VF formation is normal in these lines (and only the translation is inefficient) or the VF formation is already inefficient-aberrant.
8, VCS, DCP1 and DCP2 are the core decapping factors and they are all components of PBs. In CaMV infected plants VCS is recruited to VFs, while DCP1 is not (DCP2 was not studied). VSC, DCP1 and DCP2 null mutant are not viably in the Col. background, thus their role in CaMV replication/infection could not be analyzed. However, hypomorph DCP mutants are available (and vcs is viable in the Ler. background). It would be informative to see whether they also contribute to the CaMV infection or not.
9, Ribosome profiling subtitle (in the Methods section) is misleading, it is more frequently used for a different assay.

Reviewer #3 (Comments for the Author):
In this manuscript, authors found that several PB components are co-opted into viral replication factories to support virus multiplication. The pro-viral role of PB components as essential helpers in viral RNA translation was firstly identified. PB dysfunctions expose the virus to RNA silencing, while CaMV RNA that undergo RNA Quality Control dependent RNA degradation remains stable, but becomes translationally repressed through decreased ribosome association. These results reflect a unique dependence between PBs, RNA silencing and translational repression. Authors claim that PB components are co-opted by the virus to maintain efficient translation, a mechanism not associated with canonical PB functions.

Generally speaking, many conclusions in this manuscript are quite novel. However, many data are missing that make these results not be solid. A substantial revision is required if this is accepted in Plant Cell. Please see the detailed comments.

Major Comments:
(1) Figure 1: The conclusion from Line160-162 is conflicted with Fig 1E; The aggregates of these PB proteins may be undergoing the degradation, so the protein accumulations of these PB components should be analyzed by WB;
(2) Figure 2: Marker of cytoplasmic ribonucleo-protein (RNP) complexes should be applied in this image to analyze the co-localizations; 'Viral infection' should be added to Figure 2A;
(3) Figure 3-4,6-7 : The confirmation of all single mutants, double mutants of Arabidopsis plants should be provided by PCR and RT-qPCR; There exist some mistakes in the information of these mutants: dcp1 mutation is not lethal to plants [editor's note: SALK_014408C, described in Li and Wang, 2018], and the xrn4 mutant showed mild serrate phenotype which is not observed in this picture; It is hard to see the viral symptoms, authors should provide detailed viral symptoms images; Why authors used short day conditions for infection experiments, which is a stress condition for Arabidopsis plants that live in long-day condition naturally. These results should be performed in Arabidopsis plants with long-day condition. 'Arabidopsis and Nicotiana *benthamiana* plants were grown in walk-in chambers in standard long day conditions (16h light / 8h dark cycle) at 22°C and 65% relative humidity for crossing, propagation, and transient expression assays. For infection experiments, plants were grown in short day conditions (120 mE, 10h light / 14h dark cycle) at 22°C and 65% relative humidity.'
(4) Why the results from Line213-218 conflicted with 'Hafren et al., 2017', the explanation is not reasonable;
(5) In the whole manuscript, the mutant of xrn4 showed susceptibility to viral infection, indicating that RNA decay is an anti-viral role. If the PB components were co-opted in VFs for viral translation, while how XRN4 targets and degrades viral RNA, authors should provide more evidences to confirm XRN4-mediated viral RNA decay independent of these PB components.
(6) Figure 6: Attenuation of CaMV titers in dcp5 mutants is not mediated through NMD. This conclusion could not be obtained based on the presenting evidences: upf1 had no obvious role in CaMV titers, so the double mutation of dcp5 /upf1 displayed similar phenotype to the single mutation of dcp5 or upf1. These results indicated that UPF1-dependent NMD has no role in viral infection, how about other mutation of NMD factors to viral infection? Authors should provide more evidences on NMD pathway.
(7) PBs constituent a site that suppresses translation in plants, then how it is possible that PB components are co-opted into viral factories in complexes through viral RNA binding? (to protect Viral RNA ? Model I); XRN4 is also component of PBs, then why only XRN4 is left in the P-body without being co-opted into viral factories? the co-localization of XRN4-YFP with VFs should be analyzed in viral infection.
(8) Fig 7, 'Defects in PB integrity expose CAMV to RNA silencing but not NMD', this part does not help in the study of PB components in facilitating CAMV translation.
(9) Paradoxically, if PBs could suppress RNA silencing (RDR6)-mediated anti-viral role, so the mutation of PBs and RDR6 is supposed to affect viral RNA accumulation to some extent, while the results from Fig 7C (Viral RNA not DNA should be analyzed) and Fig S6 did not show this; Viral RNA analysis is missing in many results;
(10) The defect of viral protein production in lsm1 and dcp5 mutants is mediated through the cytoplasmic PTGS pathway governed by RDR6 and DCL proteins. Therefore, PB components facilitate CaMV infection by directly antagonizing RNA silencing, which could not reflect the title 'Cauliflower Mosaic Virus Utilizes Processing Bodies to Escape Translational Repression in Arabidopsis'; the localization of RDR6-GFP should be also observed under viral infection.

Minor comments:
(1) Figure S1： bar fonts are different,
(2) Figure S2, S4： bars are missing in some panels;
(3) Figure S3： bright filed was only shown in two co-focal images?
(4) Figure 4E: the differences in these mutants are obvious, the authors should provide early VIGS symptoms and PDS silencing efficiency.

TPC2022-RA-00174 Submission received **Feb. 15, 2022**

Reviewer comments on previously declined manuscript and **author responses:**

Reviewer #1:

Point 1. The title "Cauliflower Mosaic Virus Utilizes Processing Bodies to Escape Translational Repression in Arabidopsis" is somewhat misleading. In this study, PBs are not the site for sequestering the viral RNAs, nor for active translation.

> **RESPONSE: We have changed the title to remove the incorrectly used PB term to: *Arabidopsis* LSM1 and DCP5 proteins aid evasion of translational repression during *Cauliflower Mosaic Virus* infection**

Point 2. The authors claimed that RDR6-mediated translation repression can act on viral RNAs (Figure 8 and in Discussion). This statement was probably made by prior observation that 35S viral RNA is resistant to gene silencing and no viral RNA changes were seen in this study (Figure 3, S6). It is confusion to the readers that, instead of checking the translation repression roles, the authors were investigating the contribution of RDR6-mediated gene silencing in Figure 6. Whether RDR6 really renders the translation capacity of 35S viral RNAs could at least be tested/compared via the polysome association in mutants defective in both PB component and in RDR6/DCLs. Further increased polysome association will likely be seen in dcp5 rdr6 mutant, compared to the single mutants? And, to rule out the RDR6-mediated gene silencing, there should be no increased 35S-derived siRNAs in PB mutants?

> **RESPONSE: *lsm1/rdr6* and *rdr6* mutants, showing that the 35s RNA - polysome association defect in *lsm1* and *dcp5* is restored by *rdr6* in Figure 7. In addition, we profiled small RNAs in all genetic backgrounds and found no apparent difference between Col-0 and single or double mutants (Figure 6).**

Point 3. The biological significance of the PB component facilitated viral RNA association with VFs could be strengthened by the reduced association of viral RNAs with VF in PB mutants?

> **RESPONSE: Indeed, we have over the years made several attempts to obtain pure VF isolations, but this is challenging. However, using differential centrifugation, we can enrich and separate VFs from the cytoplasm but not from e.g. nuclei, aggregates, starch and so forth. Using this approach, we do not see any difference between WT and dcp5 for viral RNA (or P6 protein) partitioning between the sediment and soluble fractions, but we are not confident in the interpretation of this data and have not included it. It could be added upon request. We have also tried many times to obtain P6-mRFP transgenic lines in *lsm1* and *dcp5* backgrounds to evaluate VF morphology and potential alterations, but these lines do not survive to set seed and thus have prevented valid infection experiments.**

Point 4. Some data presentation will benefit from the perspective of quantification. For example, in Figure 5 the differential association of 35S RNA with polysomes in wild type than in PB mutants was used to infer a higher translation rate of viral RNA in the presence of PB components. Instead of profiling the fractions 1-12 which cover monosome to high order polysome fractions, **the free viral RNAs or those under small subunit scanning were not included (except the total amount was measured in Figure 5C)**. It may be helpful to compare the viral RNA association with different sucrose gradient fractions for free, subunits/monosomes and polysomes in different genetic backgrounds to show the differential distributions.

> **RESPONSE: We agree and have now included five fractions ranging from free RNA to heavy polysomes.**

Point 5. Spike-in RNAs should be used to ensure the differences were not a results of RNA extraction efficiencies among fractions.

RESPONSE: When designing the experiments, different normalization strategies were discussed including spike ins, but we decided at that point not to use this. We believe that the highly consistent differences between WT and PB mutants as well as the additional consistency with *rdr6* mutants rule out random extraction possibilities, as well as the *PP2a* reference that has behaved similar over altogether 12 fractionations (Figure 7C) and NMD targets.

Point 6. Similarly, in Figure 6, RSP6 and SMG7 have increased association with mono-polyribosomes. Is this due to enhanced translation in dcp5 or simply because their mRNAs levels are increased in dcp5? Also, Figure 7D needs biological replicates or quantitative data to reinforce the conclusion.

RESPONSE: The fractions are normalized to input RNA accounting for differences in mRNA levels. We have added a quantification of the Western blots from 3-4 biological replicates (Figure 7).

Point 7. Lines 163-166. One cannot rule out translational or post-translational (increased protein stability) regulation of PB mRNAs upon virus infections.

RESPONSE: "...showing that the dynamic shifts in foci counts and morphology are likely a re-distribution of previously soluble protein rather than transcription-driven *de novo* protein synthesis" was taken out and protein blots of DCP1 and VCS have been added to show protein accumulation.

Point 8. Lines 174-176. Data in Supplemental Figures 3 and 4 do not support PB association with VFs.

RESPONSE: It does in a sense as P6-foci association indicates VFs during infection. We have clarified the main text.

Reviewer #2:

Point 1. The study is not complete. The final and most important result is that, in the absence of P-body components silencing machinery represses translation of viral RNA. Unfortunately it is not analyzed in proper details. To prove that normal translation is restored in the double mutants, polysome profile assays should be also conducted using the PB-silencing double mutant plants (lsm1/rdr6, dcp5/rdr6, dcp5/dcr234). As the lines are there and the methodology is in hand, it can be conducted easily.

RESPONSE: We have complemented the polysome profiling with *rdr6* and *dcp5/rdr6* and *lsm1/rdr6*, indeed showing the expected restoration of viral RNA at polysomes by rdr6 in the *dcp5* and *lsm1* backgrounds.

Point 2. I think that comparative small RNA seq assays should also be conducted to unravel whether silencing based translational repression is associated with accumulation of specific viral sRNAs or not.

RESPONSE: We have included small RNA seq analysis showing that both quantity and genomic distribution patterns of viral small RNAs are unaltered by *dcp5*, *lsm1* and their combinatorial mutants with *rdr6*.

Point 3. The manuscript is not carefully written, the authors did not use the definitions coherently, which is misleading at many points. They should very carefully re-read their own manuscripts and use the terminology coherently. For instance, the title is misleading, overstating. It says that CaMV "utilizes processing bodies to escape translational repression". I think it is not correct, they found that certain P-body components are relocalized into VFs (while DCP1 not) and that two PB factors stimulates viral translation/replication. But they did not show evidence that PB is directly used for evasion of translational repression. They should say instead that CaMV utilizes certain processing body components to escape translational repression. Actually at the end of abstract they wrote more correctly: "PB components are co-opted by the virus to maintain efficient translation". Please correct it in the title and throughout the manuscript.

RESPONSE: We agree with this comment and have revised the manuscript accordingly.

Point 4. DCP1 is not recruited to the VF and its role in CaMV infection was not studied. Despite in their model (FIG8) they show it as part of PB that is recruited to the viral RNA and P6. It is a very speculative element (which is actually

not supported by the results). So the figure should be modified or they should note that it is highly a speculative element.

RESPONSE: DCP1 is still recruited adjacently to VFs, but not in the same manner as the other markers. We have modified the figure so that DCP1 surrounds the VFs adjacently but not inside.

Point 5. The text is confusing at certain points. For instance, in the abstract they write viral replication factories, later they write Viral Factories (VF) and emphasize that "These VFs are structurally different from the membranous RNA virus replication complexes" (Page 13). It is confusing.

RESPONSE: We have streamlined the manuscript and now only use "viral factory" and not "viral replication factory".

Reviewer #3:

Point 1. Figure 1: The conclusion from Line160-162 is conflicted with Fig 1E; The aggregates of these PB proteins may be undergoing the degradation, so the protein accumulations of these PB components should be analyzed by WB.

RESPONSE: We have included a Western blot of DCP1 and VCS in Figure 1.

Point 2. Figure 2: Marker of cytoplasmic ribonucleo-protein (RNP) complexes should be applied in this image to analyze the co-localizations; 'Viral infection' should be added to Figure 2A;

RESPONSE: We apologize for not exactly understanding this comment, as Figure 2A is a co-localization analysis between VF P6 marker (magenta) and different components of the PB RNP (DCP5, VCS...). In the figure legend, it is clearly stated that Figure 2A images are from infected tissue. We have now added the term "viral infection" to the image for clarity.

Point 3. Figure 3-4,6-7: The confirmation of all single mutants, double mutants of Arabidopsis plants should be provided by PCR and RT-qPCR; There exist some mistakes in the information of these mutants: dcp1 mutation is not lethal to plants [editor's note: SALK_014408C, described in Li and Wang, 2018], and the xrn4 mutant showed mild serrate phenotype which is not observed in this picture; It is hard to see the viral symptoms, authors should provide detailed viral symptoms images.

RESPONSE: Mutants have all been described before, are distinguishable by phenotype and all RDR6 mutants have been genotyped by small RNA sequencing in addition to conventional PCR genotyping. The described line (SALK_014408C) is a hypomorph not a complete knock-out, we have included two dcp1 alleles in the paper now (homozygous dcp1-3 and heterozygous dcp1-1).

Point 4. Why authors used short day conditions for infection experiments, which is a stress condition for Arabidopsis plants that live in long-day condition naturally. These results should be performed in Arabidopsis plants with long-day condition. 'Arabidopsis and *Nicotiana benthamiana* plants were grown in walk-in chambers in standard long day conditions (16h light / 8h dark cycle) at 22°C and 65% relative humidity for crossing, propagation, and transient expression assays. For infection experiments, plants were grown in short day conditions (120 mE, 10h light / 14h dark cycle) at 22°C and 65% relative humidity.'

RESPONSE: It is an equally common practice to do infection experiments in long- and short-day conditions, and many times developmental phenotypes of mutants are even attenuated in short-day as e.g. xrn4 lacks serration in short-day. We use short day conditions to avoid the flowering transition that might be altered in some mutants and leads to unwanted variation in disease. It is interesting to say that Arabidopsis Col-0 is stressed in our short-day but otherwise optimal conditions compared to living outdoors naturally, as our uninfected plants look very healthy and infections are carried out at an early time-point. It is unreasonable and perhaps even questionable scientific practice to request us to repeat the whole study in a different light regime that feels closer to those the reviewer prefers.

Point 5. Why the results from Line213-218 conflicted with 'Hafren et al., 2017', the explanation is not reasonable.

RESPONSE: We apologize for the unclarity, we referred to Hafren as it used the same way of measuring infection-caused biomass loss, but the referencing here is actually not necessary and we removed now the reference. There

is no conflict with previous results on a fully different topic but the same virus system.

Point 6. In the whole manuscript, the mutant of xrn4 showed susceptibility to viral infection, indicating that RNA decay is an anti-viral role. If the PB components were co-opted in VFs for viral translation, while how XRN4 targets and degrades viral RNA, authors should provide more evidences to confirm XRN4-mediated viral RNA decay independent of these PB components.

RESPONSE: We do not agree with *xrn4* showing susceptibility to virus infection throughout the manuscript. In fact, xrn4 only seems to accumulate more P6 protein, and also 35S RNA when combined with rdr6. Viral genomic DNA is the essential component of this virus particle and this readout never showed a quantitative effect in any *xrn4* context, questioning the biological/immune relevance of the few other *xrn4* effects. But, we pursued DCP5 and LSM1 in this manuscript and omit any deeper analysis of xrn4 as we have clearly established that it does not behave like the others, and quite Col-0 WT-like.

Point 7. Figure 6: Attenuation of CaMV titers in dcp5 mutants is not mediated through NMD. This conclusion could not be obtained based on the presenting evidences: upf1 had no obvious role in CaMV titers, so the double mutation of dcp5 /upf1 displayed similar phenotype to the single mutation of dcp5 or upf1. These results indicated that UPF1-dependent NMD has no role in viral infection, how about other mutation of NMD factors to viral infection? Authors should provide more evidences on NMD pathway.

RESPONSE: We addressed whether NMD becomes activated and suppress CaMV accumulation when dcp5 is disrupted. Indeed, as pointed out UPF1 does not suppress CaMV either in WT (upf1) or in the dcp5 background (upf1dcp5). We chose not to pursue the NMD direction further after our parallel finding that rather the RNA silencing pathway and specifically rdr6 and dcl2,3,4 could revert the dcp5 and lsm1 defect in virus accumulation. We believe that already upf1 is good evidence to exclude NMD as a likely component of the RDR6 and DCL activated suppression in dcp5 background.

Point 8. PBs constituent a site that suppresses translation in plants, then how it is possible that PB components are co-opted into viral factories in complexes through viral RNA binding? (to protect Viral RNA ? Model I); XRN4 is also component of PBs, then why only XRN4 is left in the P-body without being co-opted into viral factories? the co-localization of XRN4-YFP with VFs should be analyzed in viral infection.

RESPONSE: PBs are large phase-separated RNP granules, but this condition is not the only one for PB components. Our present work and studies referred to within our manuscript clearly show that VCS, DCP5 and LSM1a all also have a clear presumably cytoplasmic distribution outside of PBs, while the majority of DCP1 seems to be in constitutive PB granules. PBs are most likely functionally diverse and heterogenic even though our current knowledge of this is very limited. The VF is not at least a canonical PB, and we have tested four canonical PB markers of which three localizes. We believe this is sufficient for our conclusions and whether XRN4 localizes in VFs or not, would not progress our mechanistic understanding of DCP5 and LSM1 components in VFs. Nevertheless, we would have gladly analysed an XRN4-YFP line if we had one available.

Point 9. Fig 7, 'Defects in PB integrity expose CAMV to RNA silencing but not NMD', this part does not help in the study of PB components in facilitating CAMV translation.

RESPONSE: We think the existing literature with observations suggesting links between NMD and PBs, even for CaMV P6 (Lukhovitskaya *et al*., 2019), makes it important to ask whether our PB phenotype on CaMV infection is NMD associated.

Point 10. Paradoxically, if PBs could suppress RNA silencing (RDR6)-mediated anti-viral role, so the mutation of PBs and RDR6 is supposed to affect viral RNA accumulation to some extent, while the results from Fig 7C (Viral RNA not DNA should be analyzed) and Fig S6 did not show this; **Viral RNA analysis is missing in many results**.

RESPONSE: We have included the requested viral RNA levels (Figure 5E in the new manuscript structure), showing no difference except for a trend of increased RNA in xrn4 rdr6. Viral RNA is missing from the upf1 (Figure 5A) and the additional mutant analyses that were requested for (Figure S5). Though this comment is valid to some extent, we have clearly established that it is not RNA levels but eventually how it is being used in translation that matters and causes a quantitative decrease in viral DNA accumulation. Thus, viral DNA is the primary readout of virus accumulation defects in this phenotype, while of course RNA assays for the mechanisms are important as done.

TPC2022-RA-00174 1st Editorial decision – *revision requested* **March 26, 2022**

The reviewers agree that the current submission has been substantially improved, but that there are still elements of the manuscript that need to be addressed. As you will note in the comments, only one reviewer (#3) has requested three experimental additions. However, following the post-review discussion, we feel that while any data added in these respects would strengthen the manuscript, it is not essential for ultimate acceptance of the current manuscript.

Nonetheless, the manuscript requires considerable revisions. The reviewers have brought up multiple points regarding apparent contradictions in the presentation and interpretation of several results. As with the original submission, the authors have concerns about xrn4. For example, there are apparent contradictions in the accumulation of viral proteins in Figures 3 and 5 (see below) and in the involvement of RDR6 (see comment 2, reviewer #1). Likewise, there are multiple comments about the presentation of the data in Figure 7. A revised version of the manuscript will need to address these issues either with new data or with explanations and/or toning down of certain conclusions.

-- Reviewer comments:

Reviewer #1 (Comments for the Author):
This reviewer appreciated the authors for their efforts in revising the title which much better reflect the key discoveries made. The authors also included additional experiments to address concerns previously raised, as well as attempted to enhance the conclusions via re-organization of the figures and text. However, there are some inconsistencies between the new data and the descriptions, demanding further clarifications. More specifically:
1. The description and discussion should be focusing on "PB components" instead of "PBs" throughout the manuscript to correctly reflect the present title, the study conducted and conclusions drawn.
2. Small RNA sequencing data in Figure 6 confirmed that CaMV-sRNA is independent of RDR6 as previously reported, but also generally not affected both in size distribution and in abundance in lsm1 and dcp5 mutants (this part of data is new). This is somewhat contradictory to the model in Figure 8 that, without LSM1 or DCP5, the viral RNAs will be more vulnerable to gene silencing therefore more CaMV-sRNA should be produced? If not, is this something to do with the reduced viral DNA as measured in Figure 5C? Also, if the CaMV-sRNA is mostly RDR6-independent, how important is the RDR6 silencing machinery contributing to the silencing of viral RNAs as depicted in Figure 8?
3. Results shown in Figure 6F-I further supported TAS-mediated gene silencing suppressed by CaMV is largely independent of LSM1 and DCP5. And together with the viral DNA rescue shown in Figure 5C, the authors concluded "...RDR6-dependent suppression in lsm1 and dcp5 backgrounds does not involve viral RNA degradation or transcriptional silencing" (lines 335-336). This again argues against the relevance of the RNA silencing machinery presented in Figure 8. The authors did discuss this may not necessarily be a discrepancy. However, the results shown only supported the involvement of RDR6, but the conclusion was stretching to the RDR6-mediated gene silencing which was not supported by the data. If DCP5 and LSM1 have functions in VF (not in PBs) as discussed in this study, perhaps RDR6 also have roles in addition to gene silencing upon CaMV infection?
4. For Figure 7B, the legend indicated PP2a RNA abundance was normalized to the input RNA (also in response letter) and the housekeeping SAND gene, but the Methods described the RNA levels were normalized to SAND in each fraction and depicted as % of total RNA (lines 628-629). There are a few points to be clarified. For example, what is the distribution of SAND in polysome gradient? Would it be more reasonable to normalize the relative % to total input or total SAND mRNA? Whether the PP2a levels are comparable among all genotypes should be described for Figure 7B. If the Y-axis represents relative % of abundance, numbers for PP2a do not add up to 100% in Figure 7B. Also, for viral RNA levels in lsm1 and dcp5 mutants, when adding up from all fractions, are much lower than that in Col-0 (Fig 7D), inconsistent with the results in Fig 5D. Also, some of the dots for biological replicates are likely missing.
5. The new Figure 5 C supported the lower viral DNA accumulation in lsm1 or dcp5 mutant could be rescued by introducing the rdr6 mutation into the single mutant, consistent with the conclusion that CaMV is more vulnerable to gene silencing in lsm1 and dcp5 mutants. Figure 5D showed, on the other hand, viral RNA levels are all comparable in all genetic background tested. It would be helpful if the authors could at least discuss what might be the cause of

the differences between the results for the DNA and RNA levels. Did the "level" in lines 286 to 288 "...comparable CaMV RNA accumulation between rdr6...... excludes compensation at this level as source of viral DNA rescue" mean the RNA level? In Figure 5D, if no significant difference was concluded based on statistical test, it is inappropriate to conclude both XRN4 and RDR6 restrict viral RNA accumulation in a compensatory manner.

Minor points:

1. Line 350, Figure 7D?
2. Lines 346-348, should be referring to Fig. 7D?
3. Line 392, should be components.

Reviewer #2 (Comments for the Author):

In the resubmitted version of their manuscript (titled: Arabidopsis LSM1 and DCP5 proteins aid evasion of translational repression during Cauliflower Mosaic Virus infection) the authors studied host-virus interaction in Arabidopsis-CaMV model system. They provide evidence that P-body (PB) factors of the host facilitate CaMV infection by aiding to evade the translational repression branch of RNA silencing mediated defense.

They have found that in CaMV infected Arabidopsis the LSM, VCS, DCP5 PB components accumulate in the viral factories (VF), while DCP1 is relocalized close to the VFs. They analyzed in details the role of these PB components in CaMV infection. In the lsm1 or dcp5 null mutants the infection is less efficient, the viral DNA and protein levels are severely reduced. By contrast, viral RNAs accumulate to comparable levels in the mutants and the wild-type plants. Using double mutants (lsm1/rdr6, dcp5/rdr6, dcp5/dcr234 etc.) they demonstrate that the translation repression RNA silencing (but not the RNA degradation silencing or NMD) antiviral pathway is more efficient in the absence of LSM1 and DCP5. Polysome fractionation assays prove that viral translation is inefficient in the lsm1 or dcp5 mutants (clearly explaining the low viral DNA and protein levels), while it is fully restored in the lsm1/rdr6, dcp5/rdr6 double mutants. They propose a model that CaMV recruits certain PB components to protect the virus from RNA silencing mediated translational repression.

The results are novel, interesting and convincing. In my opinion only few little issues should be addressed before publication.

Major point

1, The most important figure is Figure 7. Unfortunately, this figure is not well organized and it is poorly described in the main text, thus it is not easy to follow.

First: in the legend should present which strain was used for this assay.

More importantly, at line 347 "The reduction of viral RNA at polysomes was striking in both lsm1 and dcp5, and specific when compared to the unaltered patterns of the control PP2a (Figure 7B)". If I understood well the text, the lsm1 and dcp5 results are shown at Figure 7D middle panel. As the Fig.7 contains many elements from A to F and some of them have left middle and right panels, the figure indications should be very precise. If we should compare Fig7B left panel to 7D middle panel, please mark it.

Line 349-351 „In accordance with ELISA and Immunoblot (Figure 3E and F), the ribosome-associated viral RNA was reduced by ~50% in lsm1 and dcp5 mutants (Figure D), indicating that lower translation levels and not protein degradation is responsible for this phenomenon." Figure 7 D middle panel is missing.

Finally, the NMD part (Suppl. 10) disrupts the description. The role of silencing pathway in translational repression should be finished first and only after to describe the NMD experiment.

As this is the most relevant part of the paper, the authors should try to describe the results more clearly.

Minor points

-Line 70: presence of exon junction complex induces NMD if it present in the 3'UTR more than 50-60 nt from the stop independently whether it is result of faulty splicing or it is result of normal splicing (see SMG7). Many erroneous splicing lead to EJC deposition but if they are not present in the 3'UTR, they will not activate NMD.

- Line 87: "around PB" citations are missing
- Line 110: Mention here, which promoter were used to express the reporters.

Line 139-140: A bit confusing sentence: "Both RFP-tagged markers showed the same localization patterns as the GFP-tagged constructs in mock and HS conditions." Make it clear that "GFP-tagged" refers to the corresponding DCP1-GFP and LSM1-GFP lines and not to the co-expressed GFP-VCS.

Line 146: "The increased GFP signal in virus infected plants," which plant is referred?
Line 148-149 "The transcript levels were consistently elevated for DCP1, DCP2 and more strongly for VCS with both CaMV strains but not for the other components (Figure 1D)." Fig 1D shows that DCP5 mRNA level was significantly enhanced in CABB infected plants.
Line 185:" we next investigated the role of PBs" PB components
Line 201-202 "The relative fresh weight of CaMV infected compared to mock inoculated plants was taken as a measure for disease severity." At upf1 mutant relative fresh weight was also modified. Does it mean that disease severity is changed?
Line 254 "Our results support that viral RNAs are not major targets of LSM1-dependent". CaMV viral RNA should be written, the original sentence is too general.
Line 302: Although it is logical that only the viral sRNAs are described in details, a sentence should be added whether Arabidopsis sRNA patterns are altered or not (or was not studied).
Line 435: As NMD inhibition was not studied in details here, thus I think that this paragraph is not relevant, it might be shortened or deleted. The discussion would be easier to follow if it were separated into sections. The NMD section should be definitely discussed in a separated section.
The title is OK but not perfect, does not refer to that LSM1 and DCP5 protects from RNA silencing mediated translational repression.

Reviewer #3 (Comments for the Author):
The revised manuscript is much improved. However, some concerns need to be solved before its final acceptance in this journal.
Major comments:
1 How P6 recruits DCP5 or Lsm1 to VFs, if authors can provide the interaction evidence between P6 and components of PBs would clarify this, and make result 1 and 2 more solid and reasonable;
2 How VFs with DCP5 or Lsm1 inhibit RDR6-mediated RNA silencing?
3 How the antiviral RDR6-mediated RNA silencing mediated the translation suppression of CaMV?
These three logical evidences in the manuscript are not sufficient, or at least 1, 2 should be solved.
Other comments:
1 All results on xrn4 need to be explained better. The role of xrn4 in CaMV infection is misleading. 'Interestingly, xrn4/rdr6 exhibited a slight but consistent elevation of 35s RNA, suggesting that both proteins can restrict viral RNA accumulation in a compensatory manner' This result is conflicted with the results in Fig 3 and Fig 5. In other words, authors gave a vague conclusion on the role of xrn4 in CaMV infection. (Even if all results on xrn4 mutants are deleted, which did not affect this manuscript)
2 Fig 4, if authors claim that 'PBs do not regulate viral RNA stability ' then other mutants of PB components should be analyzed in these experiments, or at least dcp5 should be analyzed; In addition, authors should avoid overstating the results or conclusions;
Minor comments:
1 Cauliflower Mosaic Virus (CaMV) should be cauliflower mosaic virus (CaMV) according to the latest rule for naming a viral species (Not italic, not capitalized, capitalized as long as the first letter is at the beginning of the sentence), all other viral names should be checked and changed in the main text. e.g. 'Barley yellow striate mosaic virus', Cabbage leaf curl virus Potato virus A (PVA) Turnip Mosaic Virus
2 Many descriptions in the manuscript are vague, and many sentences need to be written better:
e.g. 'similar to previous observations on transgenes' in the abstract. Readers do not know what are 'previous observations on transgenes'.
'this pathway was shown to suppress infections of PTC carrying RNA viruses (Garcia et al., 2014)' RNA viruses carrying PTCs. Taken together, lsm1 and dcp5 mutants 1) do not alter viral sRNAs quantities or profiles 2), do not show elevated VIGS and 3) do not compromise the capacity of the viral silencing suppressor P6 to target the RDR6-dependent tasiRNA pathway,
The insertions of 1) 2) 3) are off-standard
3 The empty RFP or X-RFP should be used as a negative control for Fig 2A.
4 Fig 1E : a molecular scale should be provided, and the WB bands are strange in these two blots: two bands for

DCP1-GFP (please explain it), and obscure bands for GFP-VCS;
5 Fig 2E: The viral protein accumulations on xrn4 mutants are contradictory to the DNA/RNA accumulations. My suggestion is to delete all related results on xrn4. It is possible that xrn4 has a different role in the infections of two CaMV strains, authors can separate it out for further study.
-Authors show that a picture like Fig 2E in which the P6 and p4 proteins accumulations are significantly more than other lanes (We can see them obviously), but tell readers that there is no significant difference because of a large p-value, please see Fig 2F. It is not acceptable.
6 More samples need to be used for making a histogram in qPCR or RT-qPCR assays. E.g. Fig 3C 3F Fig 4A Fig 5A. The averages look very different, but the p-value is also large. However, reads would doubt the result and the conclusion when only 3 samples were used for analyses.
7 Fig8 The model is not precise, the PB components should be put in the circle (P- body); RDR6 silencing machinery is too vague.

Reviewing Editor comments:
Line 288 (related to other comment 1 of reviewer #3): "Interestingly, xrn4/rdr6 exhibited a slight but consistent elevation of 35s RNA, suggesting that both proteins can restrict viral RNA accumulation in a compensatory manner (Figure 5D)." This is indicated as being not significant and it would be advisable not to conclude too much from this.
Methods: please indicate which TRV vectors were used in this study and use proper plasmid names. The Baulcombe system is referenced in the text, but the names given in the materials and methods sound more like the Dinesh-Kumar vectors. These two vectors can behave differently.
Figure 7D: the various dots and squares are not always centered with their corresponding text.
Figure 7. in the text, the data is not presented in the same order as it is in the figure. For example, the authors reference 7A, then 7B, then D (not 7D), then 7C.
Also, in figure 7D, it would make more sense to present the lsm1 and dcp5 data before rdr6, as it is described in the text.
Also, the text refers to ribosome-associated viral RNA, but there are four different ribosome-containing fractions and the same % reduction does not seem to apply to both mutants in all fractions. Please specify which fractions you are talking about.

TPC2022-RA-00174R1 1st Revision received April 8, 2022

Reviewer comments on previous submission and **author responses**:

<u>Reviewer #1</u>

Point 1. **R1MaP1:** The description and discussion should be focusing on "PB components" instead of "PBs" throughout the manuscript to correctly reflect the present title, the study conducted and conclusions drawn.

> **RESPONSE: We agree with the reviewer and have changed our descriptions in the text accordingly. Both lsm1 and dcp5 mutants were previously shown to assemble less (DCP1-marked) PBs and thus we thought to reflect this fact in our writing, but we understand that it is not a concise description, and we cannot distinguish this with our Arabidopsis lines. We have changed "PBs" to "PB components" throughout the manuscript when applicable.**

Point 2. **R1MaP2:** Small RNA sequencing data in Figure 6 confirmed that CaMV-sRNA is independent of RDR6 as previously reported, but also generally not affected both in size distribution and in abundance in lsm1 and dcp5 mutants (this part of data is new). This is somewhat contradictory to the model in Figure 8 that, without LSM1 or DCP5, the viral RNAs will be more vulnerable to gene silencing therefore more CaMV-sRNA should be produced? If not, is this something to do with the reduced viral DNA as measured in Figure 5C? Also, if the CaMV-sRNA is mostly RDR6-independent, how important is the RDR6 silencing machinery contributing to the silencing of viral RNAs as depicted in Figure 8?

> **RESPONSE: We have revised the model in Figure 8 for clarity. Indeed, we performed the viral sRNA analysis to see whether their abundances or profiles changed. But this was not the case, and accordingly we hypothesize in the final section of the Discussion that it is not the abundance that changes but rather the already abundant viral**

sRNAs are sufficient to drive suppression in the *dcp5* background. It is noteworthy that in Col WT, viral sRNAs are produced abundantly, but they do not affect viral DNA levels, as these levels are unaltered in *dcl234* plants. Thus, we believe it is functionality rather than quantity. See next comment as well.

Point 3. **R1MaP3:** Results shown in Figure 6F-I further supported TAS-mediated gene silencing suppressed by CaMV is largely independent of LSM1 and DCP5. And together with the viral DNA rescue shown in Figure 5C, the authors concluded "...RDR6-dependent suppression in lsm1 and dcp5 backgrounds does not involve viral RNA degradation or transcriptional silencing" (lines 335-336). This again argues against the relevance of the RNA silencing machinery presented in Figure 8. The authors did discuss this may not necessarily be a discrepancy. However, the results shown only supported the involvement of RDR6, but the conclusion was stretching to the RDR6-mediated gene silencing which was not supported by the data. If DCP5 and LSM1 have functions in VF (not in PBs) as discussed in this study, perhaps RDR6 also have roles in addition to gene silencing upon CaMV infection?

RESPONSE: We have revised the model in Figure 8B to better visualize our working concept. With our current data and analysis, we cannot conclude that the translational repression in *dcp5 / lsm1* is mediated by increased sRNA abundance. The main reason why we still extend the RDR6-mediated suppression towards the RNA silencing process is the equal rescue of viral DNA and protein accumulation in *dcp5* when DCL2,3,4 are removed, strongly arguing that sRNAs are part of the suppression mechanism. However, the reviewer is right that RDR6 may well be working outside of its canonical role as we see no quantitative increase in viral sRNAs in dcp5/lsm1, thus presenting us with a likely new mechanism we hope to further refine through additional future work. Again, we like to emphasize that viral sRNAs are very abundant already in WT plants and it is possible that these sRNA populations are differently used in the P-body component mutants. We hope the new Figure 8B better reflects our results and shows the four different scenarios that CaMV maneuvers in our study.

Point 4. **R1MaP4:** For Figure 7B, the legend indicated PP2a RNA abundance was normalized to the input RNA (also in response letter) and the housekeeping SAND gene, but the Methods described the RNA levels were normalized to SAND in each fraction and depicted as % of total RNA (lines 628-629).

RESPONSE: We realized that the Figure legend did not match with the text written in the M&M section. That was our mistake, as we failed to update the new normalization after revision in the M&M section. We have updated the text to clarify the method in the results section, figure legend and M&M section. We have also re-arranged the Figure 7 for more clarity.

Point 5. **R1MaP5.1:** The new Figure 5 C supported the lower viral DNA accumulation in lsm1 or dcp5 mutant could be rescued by introducing the rdr6 mutation into the single mutant, consistent with the conclusion that CaMV is more vulnerable to gene silencing in lsm1 and dcp5 mutants. Figure 5D showed, on the other hand, viral RNA levels are all comparable in all genetic background tested. It would be helpful if the authors could at least discuss what might be the cause of the differences between the results for the DNA and RNA levels. Did the "level" in lines 286 to 288 "...comparable CaMV RNA accumulation between rdr6 excludes compensation at this level as source of viral RNA.

RESPONSE: We have updated the Results section to clarify that the decreased DNA accumulation is likely a consequence of reduced protein translation, leading to reduced particle production. The transcription of viral RNA however is not affected and thus we do not detect any differences here.

Point 6. **R1MaP5.2:** In Figure 5D, if no significant difference was concluded based on statistical test, it is inappropriate to conclude both XRN4 and RDR6 restrict viral RNA accumulation in a compensatory manner.

RESPONSE: We agree that the conclusion drawn for xrn4/rdr6 is not supported by the data and have removed the sentence completely.

<u>Reviewer #2:</u>

Point 1. **R2MaP1:** The most important figure is Figure 7. Unfortunately, this figure is not well organized and it is poorly described in the main text, thus it is not easy to follow. First: in the legend should present which strain was used for this assay.

RESPONSE: We agree and thank the reviewer for these points to help clarify our work. We have updated the Figure legend to include that this analysis was performed with CaMV strain CM1841.

Point 2. More importantly, in line 347 "The reduction of viral RNA at polysomes was striking in both lsm1 and dcp5, and specific when compared to the unaltered patterns of the control PP2a (Figure 7B)". If I understood well the text, the lsm1 and dcp5 results are shown at Figure 7D middle panel. As the Fig.7 contains many elements from A to F and some of them have left middle and right panels, the figure indications should be very precise. If we should compare Fig7B left panel to 7D middle panel, please mark it. Line 349-351 "In accordance with ELISA and Immunoblot (Figure 3E and F), the ribosome-associated viral RNA was reduced by ~50% in lsm1 and dcp5 mutants (Figure D), indicating that lower translation levels and not protein degradation is responsible for this phenomenon." Figure 7 D middle panel is missing.

RESPONSE: We have re-arranged Figure 7 for more clarity and have given every panel an identifier. We have also updated and clarified the results section.

Point 3. Finally, the NMD part (Suppl. 10) disrupts the description. The role of silencing pathway in translational repression should be finished first and only after to describe the NMD experiment. As this is the most relevant part of the paper, the authors should try to describe the results more clearly.

RESPONSE: We have re-arranged the results section to more clearly distinguish between the RNA silencing results and the NMD results. We have moved NMD to the beginning to strengthen methodology and to be able to end on the main findings of this publication.

Reviewer #3:

Point 1. How P6 recruits DCP5 or Lsm1 to VFs, if authors can provide the interaction evidence between P6 and components of PBs would clarify this, and make result 1 and 2 more solid and reasonable.

RESPONSE: While this is an interesting question, we have no reason to believe that there would be an additional interaction needed than P6/VCS for PB components to be transported to VFs (Lukhovitskaya & Ryabova, 2019). As we did not look into this or other interactions between P6 and PB components in detail, we do not want to further discuss it for this publication. It is nevertheless an interesting angle for future work.

Point 2. **R3MaP2:** How VFs with DCP5 or Lsm1 inhibit RDR6-mediated RNA silencing?

RESPONSE: Again, that is a very valuable question but not trivial to answer and would certainly require extensive experimentation to address. At the moment we can only speculate on the nature of VFs with and without PB components, but we are looking forward to investigating it more deeply in the coming years.

Point 3. **R3MaP3:** How the antiviral RDR6-mediated RNA silencing mediated the translation suppression of CaMV?

RESPONSE: Again, that is a very valuable question that would require extensive experimentation to address and we cannot answer this at this stage. We have summarized our current understanding in the new Figure 8.

Point 4. **R3OP1.1** All results on xrn4 need to be explained better. The role of xrn4 in CaMV infection is misleading. 'Interestingly, xrn4/rdr6 exhibited a slight but consistent elevation of 35s RNA, suggesting that both proteins can restrict viral RNA accumulation in a compensatory manner' This result is conflicted with the results in Fig 3 and Fig 5.

RESPONSE: We would like the reviewer to clarify where our conclusions on *xrn4* are misleading in the manuscript. *xrn4* clearly differs from *lsm1/dcp5* in its effect on CaMV, which makes sense considering that XRN4 acts downstream of decapping on RNA, but viral RNA is not a target of *lsm1* decapping. The inclusion of *xrn4* thus strengthens the specificity of *lsm1/dcp5* and in addition provides more evidence that the degradation of viral RNA is not impaired. The sentence also doesn't conflict with Figure 3, as here we have looked at the double mutant with *rdr6* while Figure 3 addresses the single mutant. We do however agree that the conclusion drawn for xrn4/rdr6 is not supported by the data and have removed the sentence completely.

Point 5. **R3OP1.2** In other words, authors gave a vague conclusion on the role of xrn4 in CaMV infection. (Even if all results on xrn4 mutants are deleted, which did not affect this manuscript).

RESPONSE: Again, we cannot state the role of XRN4 during CaMV infection, as we have not found indications that XRN4 plays a major role during it and we are quite clear in the conclusion that *xrn4* is different from *lsm1/dcp5* helping us to uncouple the translational repression from RNA degradation. We disagree that deleting all *xrn4* results would not affect the study and argue that this would actually weaken our context.

Point 6. **R3OP**2: Fig 4, if authors claim that 'PBs do not regulate viral RNA stability ' then other mutants of PB components should be analyzed in these experiments, or at least dcp5 should be analyzed; In addition, authors should avoid overstating the results or conclusions.

> **RESPONSE: Although in the Result section, we specifically discuss the role of LSM1 in the regulation of viral RNA stability, we agree that the figure 4 title is not fitting and have changed it "LSM1 does not regulate viral stability". In addition, we have added Cordycepin treatments from two infection experiments in *dcp5* and *xrn4* to supplemental Figure 6A.**

TPC2022-RA-00174R1 2nd Editorial decision – *acceptance pending* **April 14, 2022**

We are pleased to inform you that your paper entitled "Arabidopsis LSM1 and DCP5 proteins aid evasion of translational repression during Cauliflower mosaic virus infection" has been accepted for publication in The Plant Cell, pending a final minor editorial review by journal staff. At this stage, your manuscript will be evaluated by a Science Editor with respect to its presentation of scientific content, compliance with journal policies, and presentation for a broad readership.

Final acceptance from Science Editor **April 24, 2022**
